# Time-resolved circular dichroism of excitonic systems: theory and experiment on an exemplary squaraine polymer†

**DOI:** 10.1039/d3sc01674a

**Published:** 2023-07-28

**Authors:** Lea Ress, Pavel Malý, Jann B. Landgraf, Dominik Lindorfer, Michael Hofer, Joshua Selby, Christoph Lambert, Thomas Renger, Tobias Brixner

**Affiliations:** a Institut für Physikalische und Theoretische Chemie, Universität Würzburg Am Hubland 97074 Würzburg Germany; b Faculty of Mathematics and Physics, Charles University Ke Karlovu 5 121 16 Praha 2 Czech Republic; c Institut für Theoretische Physik, Johannes Kepler Universität Linz Altenberger Str. 69 4040 Linz Austria thomas.renger@jku.at; d Institut für Organische Chemie, Universität Würzburg Am Hubland 97074 Würzburg Germany; e Center for Nanosystems Chemistry (CNC), Universität Würzburg Theodor-Boveri-Weg 97074 Würzburg Germany brixner@uni-wuerzburg.de; f Freiburg Center for Interactive Materials and Bioinspired Technologies (FIT), Universität Freiburg Georges-Köhler-Allee 105 79110 Freiburg Germany

## Abstract

Experimental and theoretical foundations for femtosecond time-resolved circular dichroism (TRCD) spectroscopy of excitonic systems are presented. In this method, the system is pumped with linearly polarized light and the signal is defined as the difference between the transient absorption spectrum probed with left and with right circularly polarized light. We present a new experimental setup with a polarization grating as key element to generate circularly polarized pulses. Herein the positive (negative) first order of the diffracted light is left-(right-)circularly polarized and serves as a probe pulse in a TRCD experiment. The grating is capable of transferring ultrashort broadband pulses ranging from 470 nm to 720 nm into two separate beams with opposite ellipticity. By applying a specific chopping scheme we can switch between left and right circular polarizations and detect transient absorption (TA) and TRCD spectra on a shot-to-shot basis simultaneously. We perform experiments on a squaraine polymer, investigating excitonic dynamics, and we develop a general theory for TRCD experiments of excitonically coupled systems that we then apply to describe the experimental data in this particular example. At a magic angle of 54.7° between the pump-pulse polarization and the propagation direction of the probe pulse, the TRCD and TA signals become particularly simple to analyze, since the orientational average over random orientations of complexes factorizes into that of the interaction with the pump and the probe pulse, and the intrinsic electric quadrupole contributions to the TRCD signal average to zero for isotropic samples. Application of exciton theory to linear absorption and to linear circular dichroism spectra of squaraine polymers reveals the presence of two fractions of polymer conformations, a dominant helical conformation with close interpigment distances that are suggested to lead to short-range contributions to site energy shifts and excitonic couplings of the squaraine molecules, and a fraction of unfolded random coils. Theory demonstrates that TRCD spectra of selectively excited helices can resolve state populations that are practically invisible in TA spectroscopy due to the small dipole strength of these states. A qualitative interpretation of TRCD and TA spectra in the spectral window investigated experimentally is offered. The 1 ps time component found in these spectra is related to the slow part of exciton relaxation obtained between states of the helix in the low-energy half of the exciton manifold. The dominant 140 ps time constant reflects the decay of excited states to the electronic ground state.

## Abstract 2

A polarization grating is used to create circularly polarized pulses for the study of exciton relaxation in a squaraine polymer. Theory shows that these pulses are able to resolve populations of exciton states with a small dipole strength.

## Graphical abstract

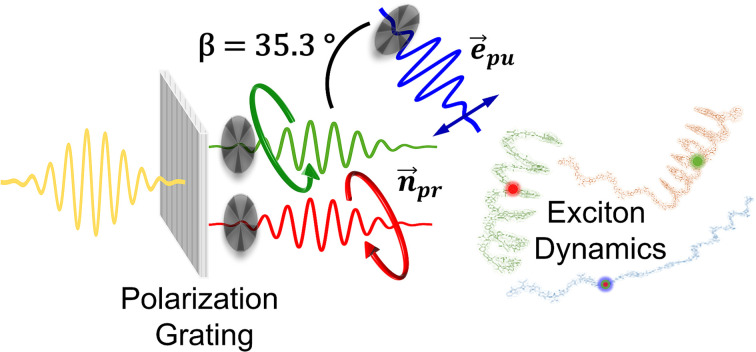

## 1 Introduction

Circular dichroism (CD) has been a valuable tool to characterize the excited states of molecular aggregates^1–8^ and polymers.^9,10^ It provides complementary information to linear absorption, since exciton states that have a small dipole strength are well resolved in CD spectra. Often, the isolated chromophore exhibits only a small intrinsic CD signal and the CD spectrum of the excitonic system reflects the chirality of the excitonic wavefunction and, hence, of the molecular aggregate or polymer. So far CD spectra of these systems have been measured in the frequency domain probing the transitions between the electronic ground state and the singly excited states. If one wants to monitor the time-dependent populations of excited states and, thereby, energy transfer, non-linear optical spectroscopy in the time domain is needed.^11,12^ Pioneering theoretical works by Cho^13^ and by Abramavicius and Mukamel^14^ predict chiral signals in 2D electronic spectra of a model dimer and photosystem I, respectively, that help to resolve overlapping optical bands. Here, we report a first experimental investigation of an excitonic system with circularly polarized pulses. A simple variant of non-linear time-resolved chiral spectroscopy is established theoretically and experimentally in the present work. In our time-resolved circular dichroism (TRCD) setup the sample is excited by a linearly polarized pump pulse and probed by left- and right-circularly polarized probe pulses. As will be shown here, with such a setup it will be possible to monitor exciton state populations that are hardly visible in an ordinary transient absorption (TA) experiment with linearly polarized pump and probe pulses.

Within time-resolved photoelectron circular dichroism (TRPECD) spectroscopy^15,16^ it is common to use vacuum-ultraviolet (VUV) pulses that pump and probe chiral molecules in the gas phase. The group of Wörner probes the chirality of photochemical dynamics in the photodissociation of CHBrFI and 2-iodobutane.^17,18^ High-harmonic generation processes are also used to investigate chiral dynamics on a sub-femtosecond timescale.^19,20^ Additional interesting TRPECD experiments were done by the groups of Blanchet,^21,22^ Baumert,^23,24^ and Heinzmann^25,26^ that show the importance of this special kind of chiral spectroscopy.

TRCD spectroscopy of dissolved molecules probed in the UV and visible wavelength region is a relatively rare technique only performed by a few groups in the world. Likely reasons for this are artifacts such as, *e.g.*, linear dichroism as well as that the signal strength is typically several orders of magnitude lower than in ordinary TA spectroscopy, and thus reaching an adequate signal-to-noise ratio is challenging. First experiments were done by David S. Kliger and co-workers with ns time resolution and single-wavelength detection.^27,28^ The same idea was followed by X. Xie and J. D. Simon with ps time resolution investigating the photolysis of carbonmonoxymyoglobin (MbCO).^29^ As third, F. Hache's group measured TRCD with single-wavelength detection^30–32^ and a time resolution of sub-picoseconds.^33,34^ Further improvements of their technique led to an experimental setup allowing them to probe conformational dynamics of several molecules in a broad ultraviolet region with fs time resolution.^35–40^ Performing TRCD with broadband visible pulses is an additional challenge typically realized in either of the following measurement modalities:^41–45^ (i) an ellipsometric measurement detects the change in ellipticity of the probe beam after it passed the chiral sample; (ii) the differential absorption method detects the difference in absorption of left- and right-circularly polarized light. Generating circularly polarized broadband probe pulses is possible either using a Pockels cell or a photoelastic modulator^46–50^ with the options to polarize a single-wavelength seed pulse which is then transformed into a white-light continuum^51–53^ or to create circular polarization of white-light continuum itself. In our own work, we developed a technique to perform polarization mirroring^54^ in order to get a mirror image of one initial circularly polarized white-light pulse.

In this study, we present a new TRCD experiment on a small footprint (40 × 60 cm^2^) that does not require active electronic polarization-modulating elements. The key element of our setup is a polarization grating which transforms a linearly polarized white-light beam into a left- and a right-circularly polarized beam within the first positive or negative diffraction order, respectively, of the grating. The temporal resolution of the experiment is determined by the pump-pulse duration which is on the order of a few tens of femtoseconds. In contrast to TRCD literature studies that generally focus on conformational transitions, here we want to explore what one can learn from TRCD concerning exciton dynamics in general. Then, exemplarily, we apply TRCD to a chiral squaraine polymer,^9^ which mainly forms a helical structure when dissolved in acetone. Due to this conformation it shows strong linear CD signals.

Polymers of squaraine chromophores with enantiomerically pure side chains are known to form helices and random coils in a certain fraction that depends on the Hansen solubility dispersion index of the solvent, the type of side group and temperature.^9^ Time-resolved pump–probe experiments were interpreted in terms of sub-ps and ps energy transfer times between helical and random coil sections of squaraine polymers in DMF (dimethylformamide) and DCM (dichloromethane) solutions and a 30–40 ps time constant for the transition between the excited and the ground electronic states.^55^ The random coil configurations of squaraine oligomers have been investigated using a Frenkel exciton model, revealing an average delocalization length of three chromophores in squaraine nonamers, which explains the exchange narrowing of the fluorescence spectrum.^56^

Exciton theory of circular dichroism has been proven to provide valuable insights into the conformation and chirality of excited states of dye aggregates,^5^ pigment–protein complexes^6^ as well as proteins.^10^ Recently, the NMR structural model^57^ of the baseplate of green sulfur bacteria has been refined by a Frenkel exciton theory of anisotropic circular dichroism (ACD).^7^ Here, we extend the exciton theory of circular dichroism by introducing transitions between the one- and the two-exciton manifold. This extension is used to describe non-linear pump–probe spectra, pumped with linearly polarized light and probed with left and right circularly polarized light. The difference in the pump–probe signal measured with the latter two circular polarizations (left minus right) defines the TRCD signal. As will be shown here, TRCD spectroscopy has the potential to monitor exciton state populations that are practically invisible in ordinary TA spectroscopy. Section 2 explains the theoretical model, Section 3 the experimental setup, and Section 4 as well as Section 5 the data in comparison between experiment and theory, respectively. We discuss the TRCD application potential and future developments in Section 6 and conclude in Section 7.

## 2 Theory

### 2.1 Exciton Hamiltonian

The excitonic system is described by the Frenkel-exciton Hamiltonian

1 *H*_exc_ = *H*^(1)^_exc_ + *H*^(2)^_exc_.

The singly excited part reads2
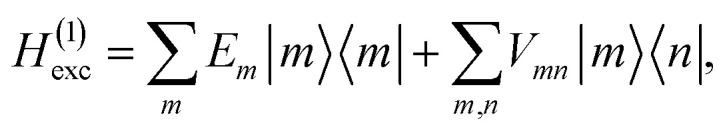
where |*m*〉 is a local excited state of the excitonic system in which pigment *m* is excited and all other pigments are in their electronic ground state. Here, *E*_*m*_ is the local excitation energy of site *m* (termed site energy),

3 *E*_*m*_ = *E*_0_ + *δ*_*m*_,

which is obtained from a reference energy *E*_0_ that is site-independent and a site-dependent energy shift *δ*_*m*_ that takes into account the electrostatic interaction between the charge densities of the ground and excited states of pigment *m* with the ground-state charge density of the remaining pigments,4
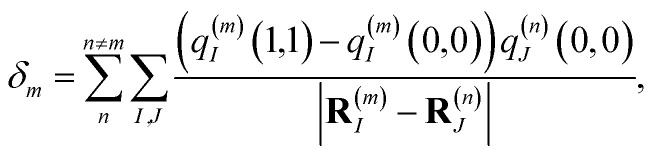
where *q*^(*m*)^_*I*_(1,1) and *q*^(*m*)^_*I*_(0,0) are atomic partial charges that are obtained from a fit of the electrostatic potential (ESP) of the charge densities of the electronic excited and ground state, respectively, of the isolated chromophore, which are placed at position **R**^(*m*)^_*I*_ of the *I*th atom of chromophore *m* and *q*^(*n*)^_*J*_(0,0) is the ground-state partial charge of the *J*th atom of chromophore *n* in the polymer. *V*_*mn*_ is the Coulomb coupling between transition densities of sites *m* and *n*, termed excitonic coupling, that is also approximated by the Coulomb coupling between atomic partial charges representing the transition densities of the pigments,5
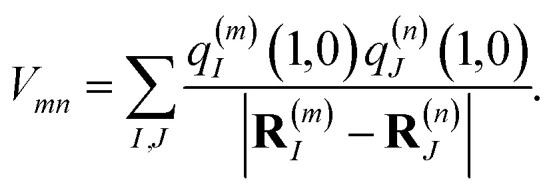


These approximations of the charge density and the excitonic couplings are known in the literature as CDC (charge-density coupling) method^58^ and TrEsp (transition charges from electrostatic potential) method.^59^ The reference energy *E*_0_ in eqn (3) takes into account the influence of the solvent environment and all non-electrostatic interchromophore interactions in an effective way. The contribution from the doubly excited states in eqn (1) reads^60^6
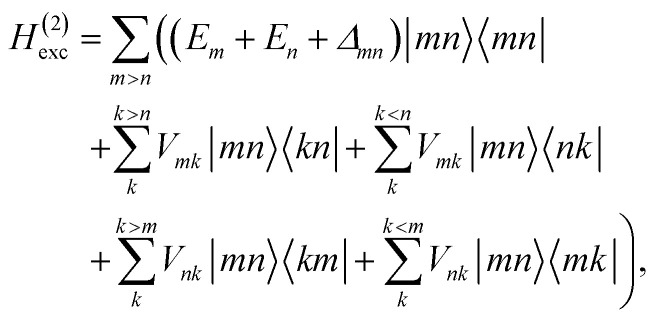
where the first term on the right-hand side contains the local transition energy of state |*mn*〉, in which chromophores *m* and *n* are excited and the remaining chromophores are in their electronic ground state and the second through fifth terms contain the excitonic coupling between the localized two-exciton states. The transition energy of a two-exciton state |*mn*〉 can be written as a sum of the local excitation energies *E*_*m*_ + *E*_*n*_ of pigments *m* and *n* plus a two-exciton shift *Δ*_*mn*_ that reads,‡‡Please note that the two-exciton shift in eqn (3) of ref. 60, due to a misprint, does not contain the term 
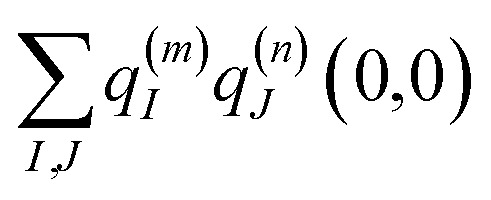
. The correct expression is given here.^60^7
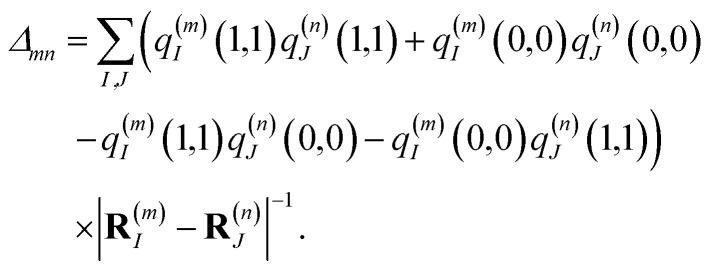


The eigenstates of *H*^(1)^_exc_ and *H*^(2)^_exc_ are the one- and two-exciton states8
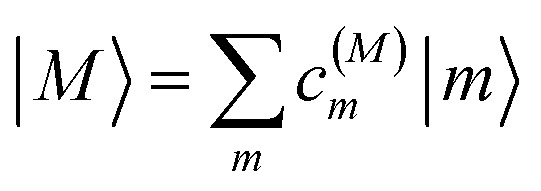
and9
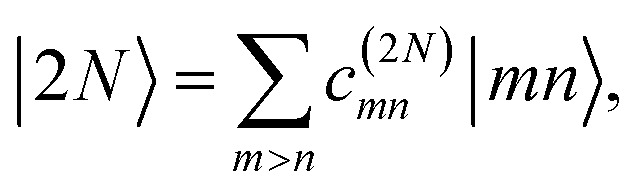
respectively. The coefficients *c*^(*M*)^_*m*_ and *c*^(2*N*)^_*mn*_ and the eigenenergies ℏ*ω*_*M*_ and ℏ*ω*_2*N*_ result from the diagonalization of the respective exciton matrix.

### 2.2 Time-dependent spectra

#### 2.2.1 Basic quantities

The pump–probe spectra are described in the spirit of third-order perturbation theory in the interaction between the radiation field and the molecular system. The pump pulse is described as10
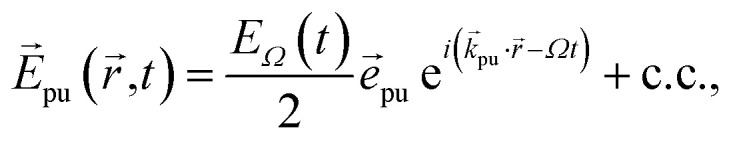
where c.c. signifies the complex conjugate of the previous term, with central frequency *Ω*, polarization unit vector *e⃑*_pu_, wave vector *k⃑*_pu_ and pulse envelope11
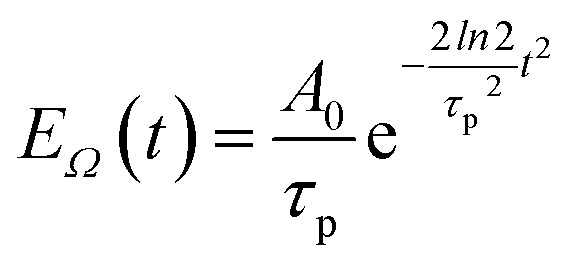
with the constant *A*_0_ and the pulse width *τ*_p_ that corresponds to the FWHM (full width at half maximum) of the intensity (∼*E*_*Ω*_^2^(*t*)) of the pulse. The pump pulse generates an initial population of one-exciton states^61^

12 *P*_*M*_(0) = |*μ*^(pu)^_*M*_|^2^*U*_*M*_,

where

13 *μ*^(pu)^_M_ = *μ⃗*_M_·*e⃑*_pu_

is the scalar product between the transition dipole moment of exciton state |*M*〉,14
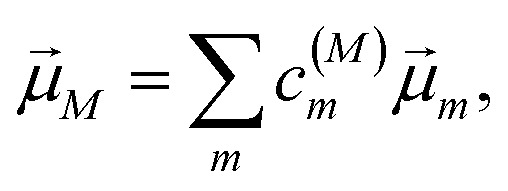
resulting from a linear combination of the transition dipole moments *μ⃗*_*m*_ of the pigments, and the polarization (unit) vector *e⃑*_pu_ of the pump field. The function *U*_*M*_ in eqn (12) takes into account the width of the pump pulse and the lineshape function of the optical transition from the electronic ground state to the one-exciton state |*M*〉 that has a maximum at roughly the exciton eigenenergy ℏ*ω*_*M*_ of this state. Details are given in Section 2.4 below. Note that we neglect the creation of an initial coherence between different exciton states by the pump pulse, because we assume that the dephasing is fast as compared to the delay time between the pump and the probe pulse.

A delay time *τ* after the pump pulse, a probe pulse with broadband white-light spectrum measures the absorption of linearly (TA) or circularly (TRCD) polarized light. The probe pulse will be approximated here by a *δ*-shaped pulse,15
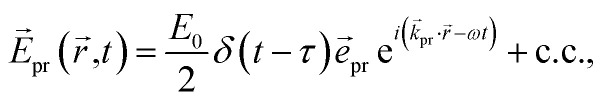
with polarization vector *e⃑*_pr_, wave vector *k⃑*_pr_, field amplitude *E*_0_, envelope *δ*(*t* − *τ*), and central frequency *ω*, where c.c. represents the complex conjugate of the previous term.

The frequency-resolved TA signal Δ*α*(*ω*,*τ*) is defined as the difference in absorbance of the probe field with and without the pump-pulse excitation,

16 Δ*α*(*ω*,*τ*) = *α*(*ω*,*τ*) − *α*_0_(*ω*),

where *α*_0_ is the linear absorbance spectrum of the molecular system and *α*(*ω*,*τ*) is the absorbance of the probe pulse interacting with the system at a delay time *τ* after the pump pulse. Similarly, the TRCD signal ΔCD(*ω*,*τ*) is defined as

17 ΔCD(*ω*,*τ*) = CD(*ω*,*τ*) − CD_0_(*ω*),

where CD(*ω*,*τ*) = *α*_l_(*ω*,*τ*) − *α*_r_(*ω*,*τ*) is the difference in absorbance between left and right circularly polarized probe pulses at a delay time *τ* after the pump pulse, and CD_0_(*ω*) is the same difference but without the pump pulse.

The TA and TRCD signals Δ*S*(*ω*,*τ*) with *S* = *α* and *S* = CD can be decomposed into three contributions:

18 Δ*S*(*ω*,*τ*) ∝ GSB_*S*_(*ω*,*τ*) + SE_*S*_(*ω*,*τ*) + ESA_*S*_(*ω*,*τ*).

The ground-state bleaching (GSB)19
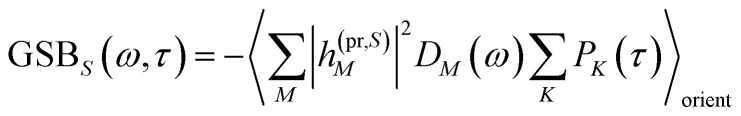
takes into account the decrease of the probe pulse absorption caused by the partial depopulation of the electronic ground state of the molecular system by its interaction with the pump pulse. Here, *h*^(pr,*S*)^_*M*_ is the matrix element of the interaction of the probe pulse with the system, to be specified further below. *D*_*M*_(*ω*) is the lineshape function of the optical transition between the ground state and one-exciton state |*M*〉. Note that the sum over one-exciton state populations *P*_*K*_(*τ*) depends on *τ*, since radiative and non-radiative transitions to the electronic ground state depopulate the one-exciton state manifold. The 〈…〉_orient_ denotes an orientational average with respect to randomly oriented molecules in the sample. The stimulated emission SE_*S*_(*ω*,*τ*) is given as20
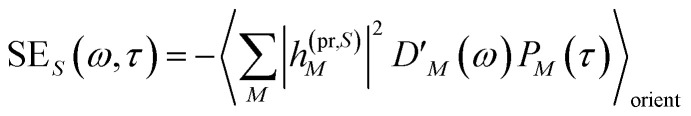
with the fluorescence lineshape function 
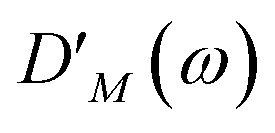
 for the optical transition between the *M*th exciton state and the electronic ground state and the population of the *M*th exciton state *P*_*M*_(*τ*). Finally, the excited-state absorption (ESA) reads21
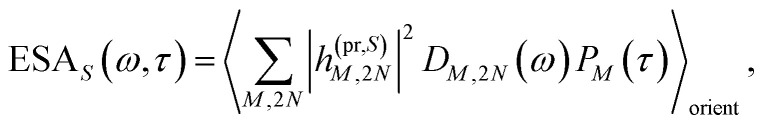
where *h*^(pr,*S*)^_*M*,2*N*_ denotes the matrix element between one-exciton state |*M*〉 and two-exciton state |2*N*〉 of the interaction of the system with the probe pulse, and *D*_*M*,2*N*_(*ω*) is the respective optical lineshape function.

In case of TA (*S* = *α*) we can apply a dipole approximation for the coupling between the external field and the excitonic system, and the matrix elements read22
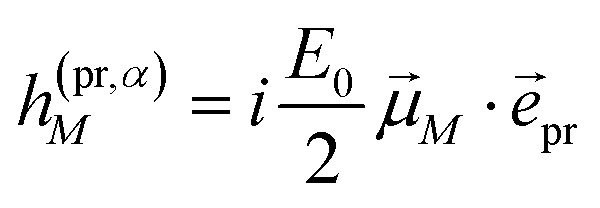
and23
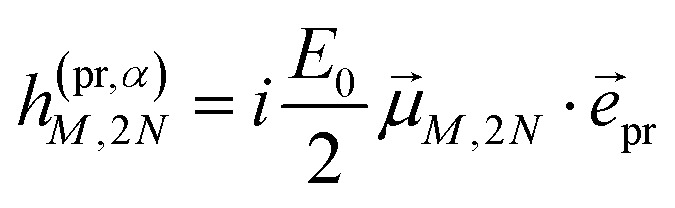
with the amplitude *E*_0_ of the probe field, the probe pulse polarization (unit) vector *e⃑*_pr_, the one-exciton transition dipole moment *μ⃗*_*M*_ from eqn (14), and the transition dipole moment *μ⃗*_*M*,2*N*_ between one-exciton state |*M*〉 and two-exciton state |2*N*〉, obtained as24
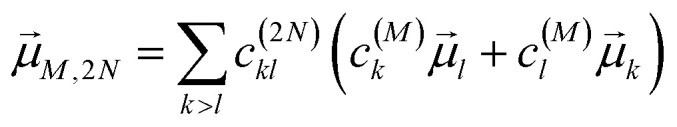
with the coefficient *c*^(2*N*)^_*kl*_ of the two-exciton state |2*N*〉, the coefficients *c*^(*M*)^_*k*_ and *c*^(*M*)^_*l*_ of one-exciton state |*M*〉, and the local transition dipole moments *μ⃗*_*l*_ and *μ⃗*_*k*_ of sites *l* and *k*, respectively. Note that we neglect intra-site ESA, that is, we treat the local sites as electronic two-level systems.

The square of the one-exciton matrix element for TRCD (*S* = CD) is obtained as^7^25
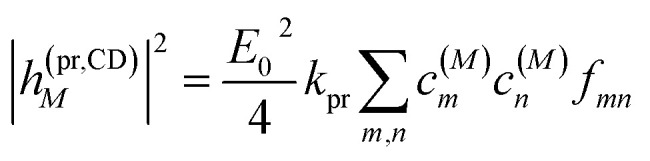
with the field amplitude and magnitude of wave vector magnitude of the probe field, *E*_0_ and *k*_pr_, respectively, and

26 *f*_*mn*_ = (*n⃑*_pr_·*R⃑*_*mn*_)(*n⃑*_pr_·(*μ⃗*_*m*_ × *μ⃗*_*n*_)) + *n⃑*_pr_·((*Q̂*_*n*_*n⃑*_pr_) × *μ⃗*_*m*_)

containing the vector *R⃑*_*mn*_ = *R⃑*_*m*_ − *R⃑*_*n*_ connecting the centers of chromophores *n* and *m*, the propagation (unit) vector *n⃑*_pr_ of the probe field and the intrinsic electric quadrupole transition moment *Q̂*_*n*_ of chromophore *n*, defined with respect to the center of the chromophore.^7^

Along the same lines, the square of the matrix element for the excitation of a two-exciton state is obtained as (details of the derivation are given in the ESI, Section S7†)27
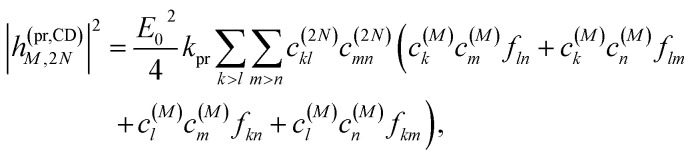
where the functions *f*_*ln*_, *f*_*lm*_, *f*_*kn*_ and *f*_*km*_ are given in eqn (26) by replacing the chromophore indices *m* and *n* accordingly.

#### 2.2.2 Population dynamics

The population dynamics of one-exciton states are governed by the master equation28
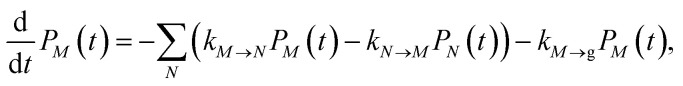
with the population *P*_*M*_(*t*) at time *t* after excitation of exciton state *M*, the rate constant *k*_*M*→*N*_ of exciton relaxation between states |*M*〉 and |*N*〉, and the rate constant *k*_*M*→g_ for irreversible radiative and non-radiative transitions to the electronic ground state. In matrix notation, the master equation is written as29
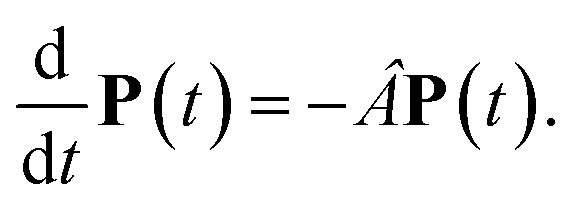


Note that in the experiment, time zero is set by the pump pulse and, hence, the time *t* at which the population enters the pump–probe signal corresponds to the delay time *τ* between pump and probe pulse. The matrix elements of *Â* are given as30
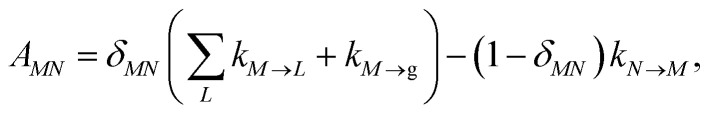
where the rate constant *k*_*M*→*N*_ of exciton relaxation between states |*M*〉 and |*N*〉 is described by using Redfield theory giving

31 *k*_*M*→*N*_ = 2π*γ*_*MN*_*ω*_*MN*_^2^{(1 + *n*(*ω*_*MN*_))*J*(*ω*_*MN*_) + *n*(*ω*_*NM*_)*J*(*ω*_*NM*_)}

with the *γ*_*MN*_ in eqn (62), the Bose–Einstein distribution function of vibrational quanta32
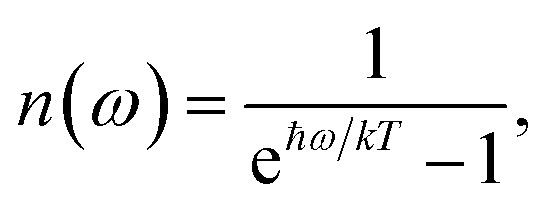
and the spectral density *J*(*ω*) that enters the rate constant at the transition frequencies between exciton states, *ω*_*KL*_ = (*E*_*K*_ − *E*_*L*_)/ℏ. Note that it holds that *J*(*ω*) = 0 for *ω* < 0. The rate constant *k*_*M*→g_ describes the decay of the exciton populations to the ground state and will be treated as a free parameter, obtained from a fit of the transient spectra.

The solution of eqn (29) is given as33
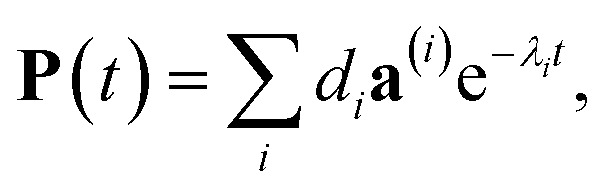
where **a**^(*i*)^ is the (right) eigenvector of *Â*, *λ*_*i*_ is the corresponding eigenvalue and the coefficients *d*_*i*_ are determined by the initial conditions **P**(0), that is, the exciton populations *P*_*M*_(0) created by the pump pulse. The exciton relaxation rate constants fulfill the principle of detailed balance,

34 *k*_*M*→*N*_*P*^(eq)^_*M*_ = *k*_*N*→*M*_*P*^(eq)^_*N*_,

with the (quasi)equilibrium populations of exciton states35
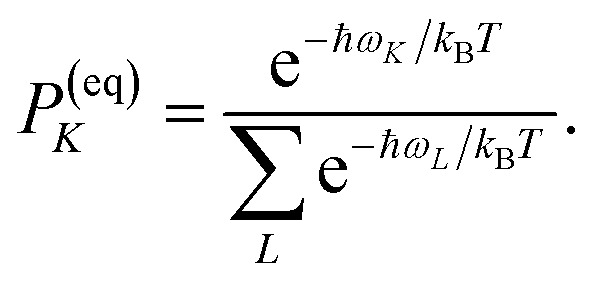


This property is used in ESI, Section S8,† to express the coefficient *d*_*i*_ as36
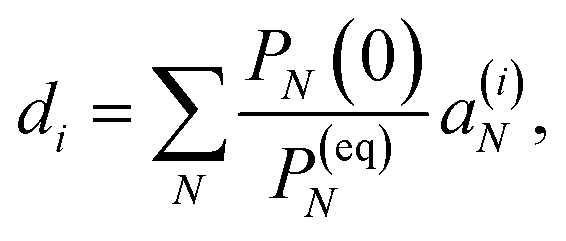
where *a*^(*i*)^_*N*_ is the *N*th component of the eigenvector **a**^(*i*)^ of the kinetic matrix *Â*. Using eqn (12), (33) and (36), the exciton population *P*_*M*_(*t*) can be expressed as37
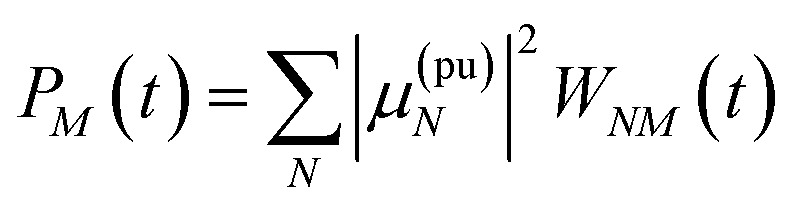
with the auxiliary function *W*_*NM*_(*t*) reading38
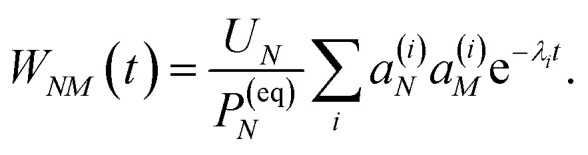


#### 2.2.3 Transient absorption

The different contributions to the TA spectrum then follow as39
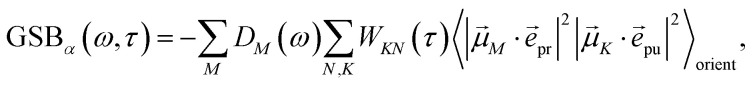
40
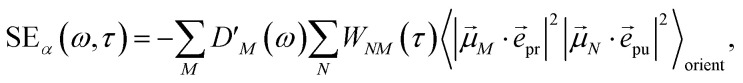
and41
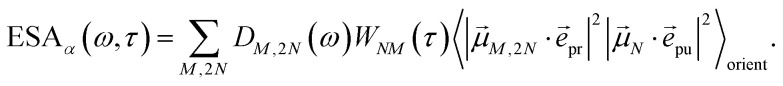


The polarization vectors *e⃑*_pu_ and *e⃑*_pr_ of the pump and probe fields, respectively, are fixed in the laboratory frame and the orientational average has to be performed with respect to the orientations of molecular transition dipole moments. Using Euler angles, the latter can be transformed from the molecular into the laboratory frame and the orientational average calculated,^62,63^ resulting in42
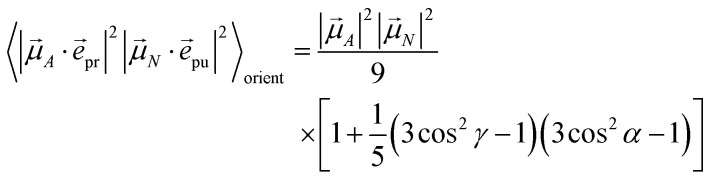
with the angle *γ* between *μ⃗*_*A*_ and *μ⃗*_*N*_ and the angle *α* between the polarization vectors *e⃑*_pu_ and *e⃑*_pr_. Note that *μ⃗*_*A*_ stands for *μ⃗*_*M*_ (eqn (39) and (40)) or *μ⃗*_*M*,2*N*_ (eqn (41)). For the magic angle *α* = 54.7° it holds that cos^2^ *α* = 1/3 and, hence, the orientational average simplifies to43
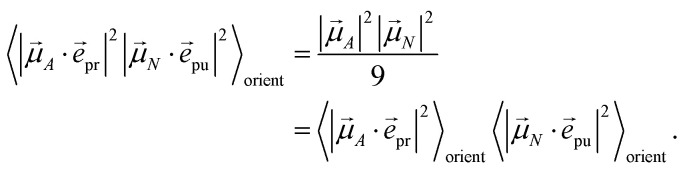


In other words, for magic-angle polarization direction of pump and probe pulse, formally the orientational average can be taken into account by replacing |*μ⃗*_*A*_·*e⃑*_pr_|^2^ in the equations of motion by its orientational average |*μ⃗*_*A*_|^2^/3 and similarly replacing |*μ⃗*_*N*_·*e⃑*_pu_|^2^ by |*μ⃗*_*N*_|^2^/3. We note that a magic-angle polarization between pump and probe fields is possible for collinear as well as non-collinear propagation directions of these fields.^64^ The situation changes for the TRCD signal discussed in the following.

#### 2.2.4 Time-resolved circular dichroism

The different contributions to the TRCD spectrum are obtained as44
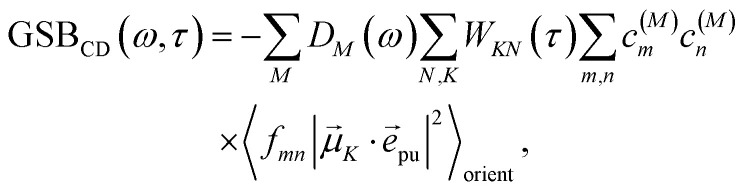
45
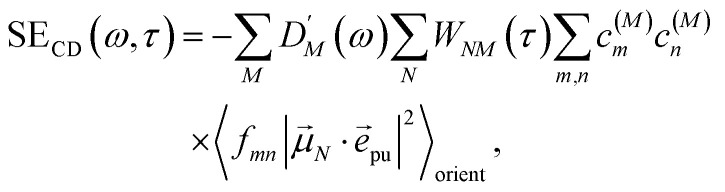
and46
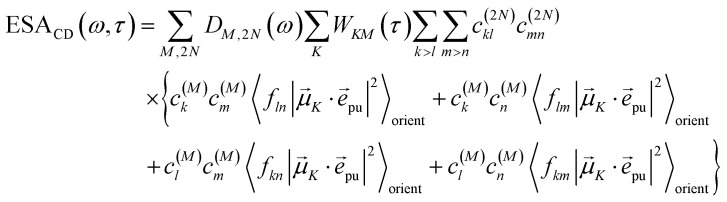
with the function *f*_*mn*_ in eqn (26). The orientational average 〈*f*_*mn*_|*μ⃗*_*K*_·*e⃑*_pu_|^2^〉_orient_ is evaluated in the ESI, Section S6† resulting in47
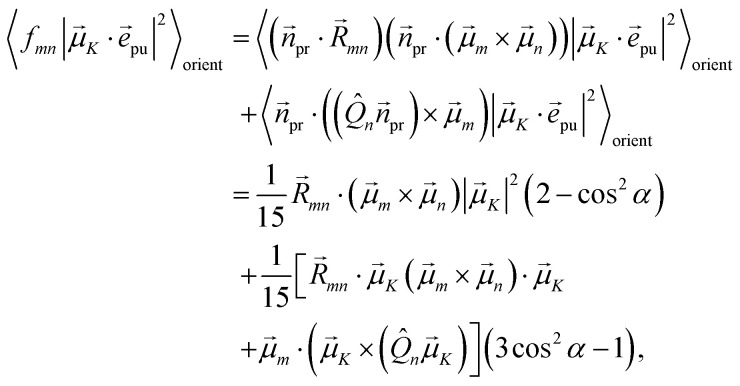
where *α* is the angle between the pump pulse polarization vector *e⃑*_pu_ and the propagation direction (unit) vector *n⃑*_pr_. Again, the magic angle *α* = 54.7° between these vectors leads to a factorization of the orientational average,48
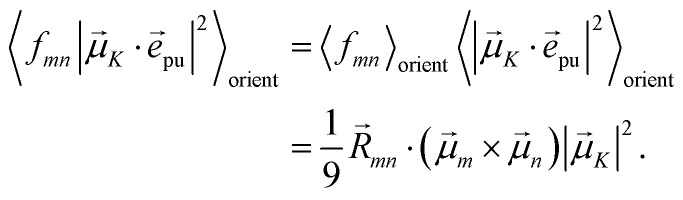


**Fig. 1:**
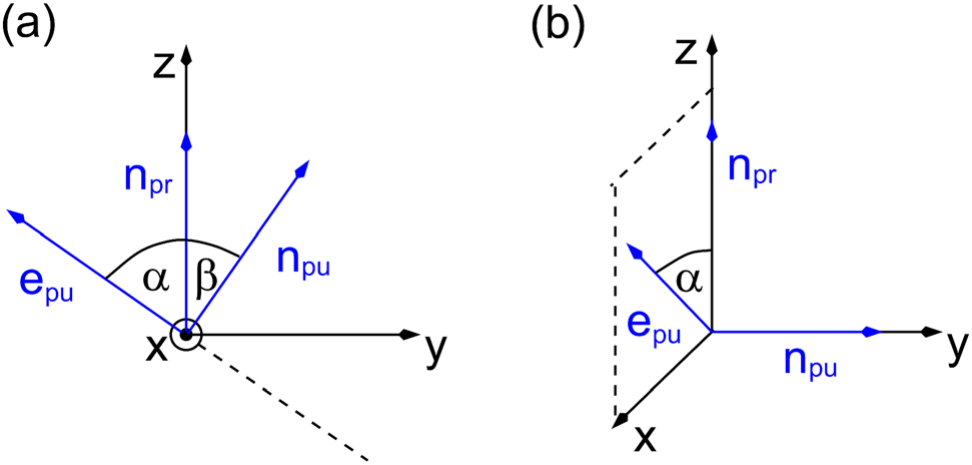
Two possible setups for the simultaneous anisotropy-free measurement of TRCD and TA spectra. (a) The pump- and probe pulse propagate at an angle of *β* = 35.3°, as described by the propagation vectors **n**_pu_ and **n**_pr_, respectively. The pump pulse is linearly polarized in the plane (here *y*–*z*) spanned by **n**_pu_ and **n**_pr_ resulting in a magic angle *α* = 54.7° between **n**_pr_ and the pump-pulse polarization vector **e**_pu_. (b) Pump- and probe pulse propagate in vertical directions (here along the *y*- and *z*-axis, respectively), and the pump pulse is polarized at a magic angle with respect to **n**_pr_ in a plane (here *x*–*z*) normal to **n**_pu_.

As a consequence of this factorization, there is no contribution from the intrinsic electric quadrupole moments of the chromophores to the TRCD signal.^13^ Note, however, that for collinear propagation directions of the two pulses, *α* = 90° results, because of the transversal character of the optical fields. Hence, in order to take advantage of the simplifications obtained for *α* = 54.7°, a non-collinear experimental setup has to be chosen for the pump and probe fields in TRCD spectroscopy.^13,64^ Two possible scenarios are depicted in Fig. 1. In setup (a), used in the present experiments, the pump-pulse polarization is in the plane spanned by the propagation vectors of the two pulses at a magic angle *α* = 54.7° with respect to the propagation vector of the probe pulse, corresponding to an angle *β* = 90° − *α* = 35.3° between the propagation vectors of the two pulses. In setup (b) the two pulses propagate in orthogonal directions and the pump-pulse polarization is in a plane orthogonal to the pump pulse polarization vector at a magic angle *α* = 54.7° with respect to the propagation vector of the probe pulse. Because of the magic angle, the orientational average relevant for the TRCD signal factorizes, as described in eqn (48).

With the same setups, an isotropic TA signal can be measured with the circularly polarized probe pulses as discussed in the following. Using either a left- or a right-circularly polarized probe field, 
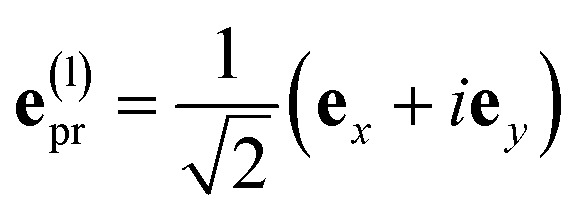
 and **e**^(r)^_pr_ = (**e**^(l)^_pr_)*, respectively, the orientational average relevant for TA (eqn (43)) is obtained as49
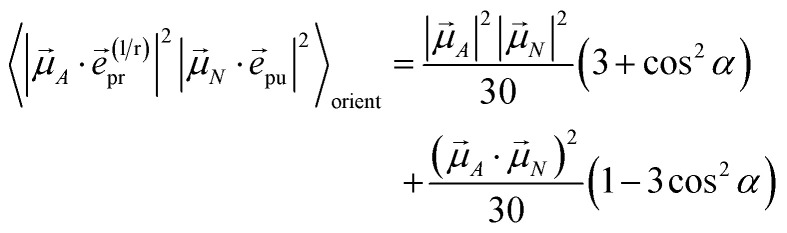
which for *α* = 54.7° (cos^2^ *α* = 1/3) becomes50
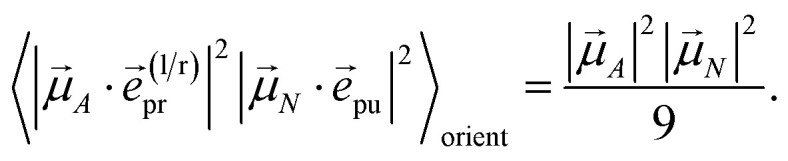


Hence, the same isotropic TA signal is measured as with a linearly polarized probe field (eqn (43)). Please find a detailed derivation of the orientational averages discussed above in the ESI, Section S6.† Note that in the present experiments, the isotropic TA signal is measured as (TA(*e⃑*^(l)^_pr_) + TA(*e⃑*^(r)^_pr_))/2, in order to reduce the signal-to-noise ratio.

### 2.3 Magic-angle TA and TRCD signals

As discussed above, for the magic angle *α* = 54.7° between pump-pulse polarization vector *e⃑*_pu_ and propagation direction vector of the probe pulse *n⃑*_pr_, the orientational average in the TA and TRCD signals factorizes into pump- and probe-pulse averages.

In this case the contributions to the time-resolved signal Δ*S*(*ω*,*τ*) (eqn (18)) become51
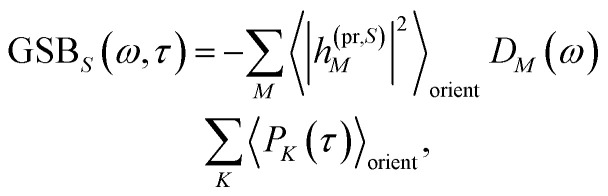
52
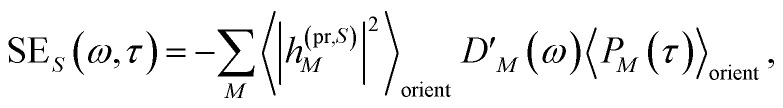
and53
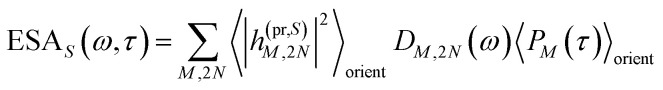
with the orientational average populations of exciton states 〈*P*_*M*_(*τ*)〉_orient_ (or *M* = *K*)54
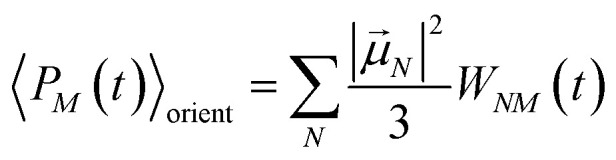
containing the auxiliary function *W*_*NM*_(*t*) (eqn (38)).

The orientational averages containing the matrix elements of the interaction with the probe pulse in the case of TA are given as55
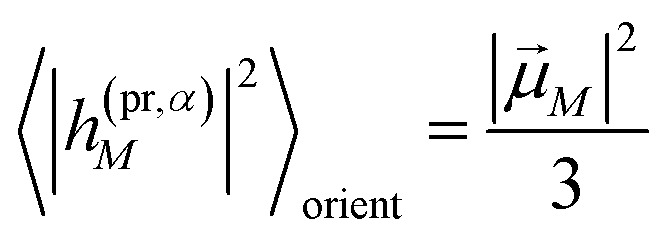
with the one-exciton transition dipole moments *μ⃗*_*M*_ (eqn (14)) and56
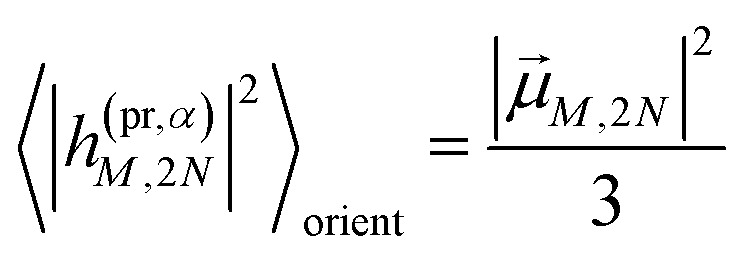
containing the transition dipole moments between one- and two-exciton states *μ⃗*_*M*,2*N*_ (eqn (24)).

The respective matrix elements for TRCD are obtained as57
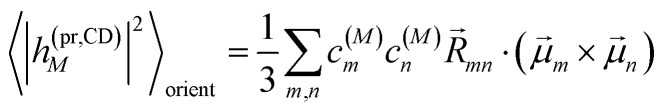
containing the coefficients *c*^(*M*)^_*m*_ and *c*^(*M*)^_*n*_ of one-exciton state |*M*〉, the vector *R⃑*_*mn*_ connecting the centers of chromophores *m* and *n*, and the local transition dipole moments *μ⃗*_*m*_ and *μ⃗*_*n*_ of chromophores *m* and *n*, respectively. The matrix element entering the ESA contribution to TRCD reads58
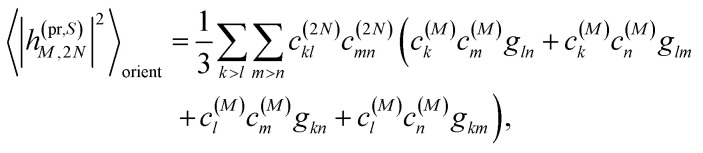
where we introduced the function

59 *g*_*kl*_ = *R⃑*_*kl*_·(*μ⃗*_*k*_ × *μ⃗*_*l*_),

and besides the coefficients *c*^(*M*)^_*k*_ of one-exciton state |*M*〉 also the coefficients *c*^(2*N*)^_*kl*_ of two-exciton state |2*N*〉.

### 2.4 Lineshape function and initial population of exciton states

We apply a lineshape theory derived earlier^65^ using a partial ordering prescription cumulant expansion and a secular and Markov approximation for the off-diagonal elements of the exciton-vibrational coupling in the exciton basis. The lineshape function *D*_*M*_(*ω*) of absorption reads^65^60
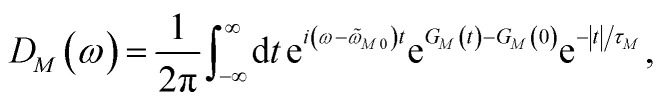
where the transition energy *ω̃*_*M*0_ contains a renormalization by the diagonal elements of the exciton-vibrational coupling

61 *ω̃*_*M*0_ = *ω*_*M*0_ − *γ*_*MM*_*E*_*λ*_/ℏ,

where *γ*_*MM*_ is the inverse participation ratio of exciton state |*M*〉 obtained from the diagonal part (*M* = *N*) of62
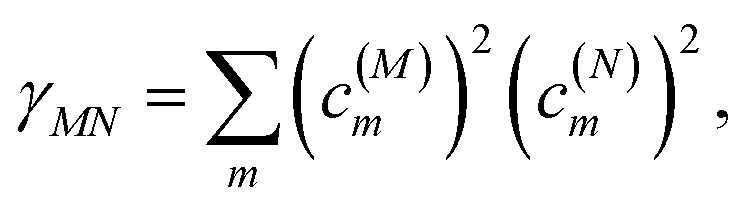
and63
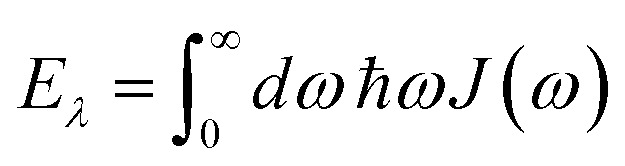
is the reorganization energy of the local optical excitation of the pigments that is obtained from the spectral density of the local exciton-vibrational coupling *J*(*ω*). Note that the original lineshape theory^65^ contains an additional shift of the transition energy by the off-diagonal elements of the exciton-vibrational coupling, which in the presence of static disorder is small compared to the diagonal part. These small off-diagonal elements are neglected here for simplicity. The inverse dephasing time *τ*_*M*_^−1^ describes the lifetime broadening of exciton state |*M*〉 and is obtained from the Redfield theory rate constants of exciton relaxation to the other exciton states,64
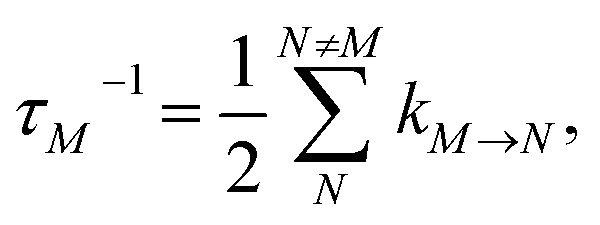
with the Redfield rate constant *k*_*M*→*N*_ in eqn (31). The function *G*_*M*_(*t*) on the right-hand side of eqn (60) reads

65 *G*_*M*_(*t*) = *γ*_*MM*_*G*(*t*)

with the *γ*_*MM*_ in eqn (62) and66
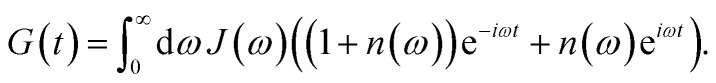


Assuming that after optical excitation the nuclei relax in the potential energy surface of exciton states, the lineshape function of ESA from one-exciton state |*M*〉 to the two-exciton state |2*N*〉 within the above approximations is obtained as^65^67
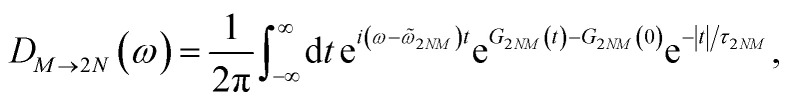
where

68 *ω̃*_2*NM*_ = *ω*_2*NM*_ − *E*_*λ*_/ℏ(*γ*_2*N*2*N*_ + *γ*_*MM*_ − 2*γ*_2*NM*_)

with the *γ*_*MM*_ in eqn (62). The function *γ*_2*N*2*N*_ is obtained from the diagonal part (2*K* = 2*N*) of69
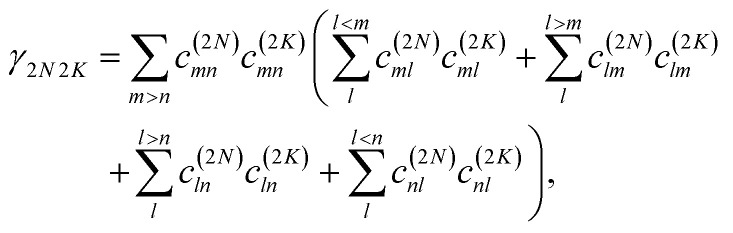
and *γ*_2*NM*_ is given as70
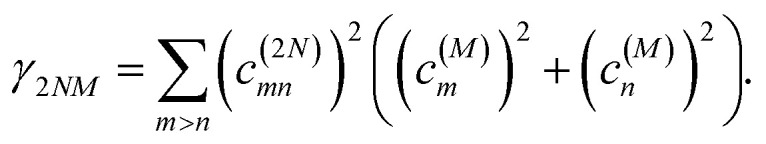


The function *G*_2*NM*_(*t*) in eqn (67) follows as

71 *G*_2*NM*_(*t*) = (*γ*_2*N*2*N*_ + *γ*_*MM*_ − 2*γ*_2*NM*_)*G*(*t*),

where *γ*_2*N*2*N*_, *γ*_*MM*_ and *γ*_2*NM*_ are given in eqn (69), (62) and (70), respectively. The inverse dephasing time *τ*_2*NM*_^−1^ is given by the inverse dephasing times of one-exciton states *τ*_*M*_^−1^ in eqn (64) and that of the two-exciton state *τ*_2*N*_^−1^,

72 *τ*_2*NM*_^−1^ = *τ*_*M*_^−1^ + *τ*_2*N*_^−1^,

with73
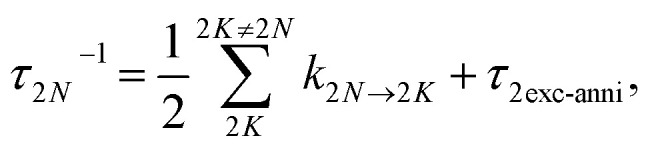
where *k*_2*N*→2*K*_ is the rate constant of exciton relaxation between |2*N*〉 and |2*K*〉,

74 *k*_2*N*→2*K*_ = 2π*γ*_2*N*2*K*_*ω*_2*N*2*K*_^2^{(1 + *n*(*ω*_2*N*2*K*_))*J*(*ω*_2*N*2*K*_) + *n*(*ω*_2*K*2*N*_)*J*(*ω*_2*K*2*N*_)},

with *ω*_2*N*2*K*_ = (*E*_2*N*_ − *E*_2*K*_)/ℏ. In addition to exciton relaxation processes, the lifetime of the two-exciton states is limited by exciton–exciton annihilation processes. A microscopic description of the latter would require to include a higher-excited electronic state of the chromophores. Here we take into account these processes implicitly by assigning a single additional dephasing constant *τ*_2exc-anni_ for all two-exciton states.

The lineshape function of stimulated emission,^65^75
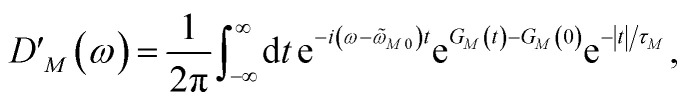
differs from that of linear absorption and GSB, *G*_*M*_(*t*), in eqn (60) just by a minus sign in the first exponent, reflecting the Stokes shift of the emission with respect to the absorption (GSB) lineshape function.

In order to obtain the initial population of exciton states created by the pump pulse, we use second-order perturbation theory in the system-field interaction, take into account the diagonal elements of the exciton-vibrational coupling in the exciton basis and neglect exciton relaxation during the action of the pump pulse. The function *U*_*M*_ for the (orientationally averaged) initial population of exciton states *P*_*M*_(0) in eqn (12) then follows as^61^76
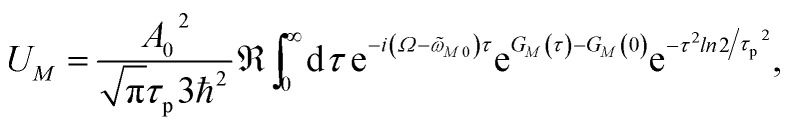
where *A*_0_ and *τ*_p_ are the amplitude and the width, respectively, of the pump pulse envelope (eqn (11)), *μ*_*M*_ is the magnitude of the transition dipole moment of the *M*th exciton state (eqn (14)), *Ω* is the central frequency of the pump pulse (eqn (10)), *ω̃*_*M*0_ is the transition frequency between the minima of the potential energy surfaces of the electronic ground state and the *M*th one-exciton state (eqn (61)), and the function *G*_*M*_(*τ*) describes the excitation of the vibrational sideband of the *M*th exciton state (eqn (65)). Comparing the above expression with the lineshape function of linear absorption, *D*_*M*_(*ω*) (eqn (60)), we notice a close relation: the *ω* is replaced by the central frequency *Ω* of the pump pulse, and the linear dephasing with time constant *τ*_*M*_, leading to lifetime broadening in the linear absorption spectrum, is replaced by a quadratic dephasing with decay constant *τ*_p_^2^/*ln*2 reflecting the fact that the shorter the pulse width *τ*_p_ the larger is the spectral width of the pulse.

## 3 Experiment

### 3.1 Setup

**Fig. 2:**
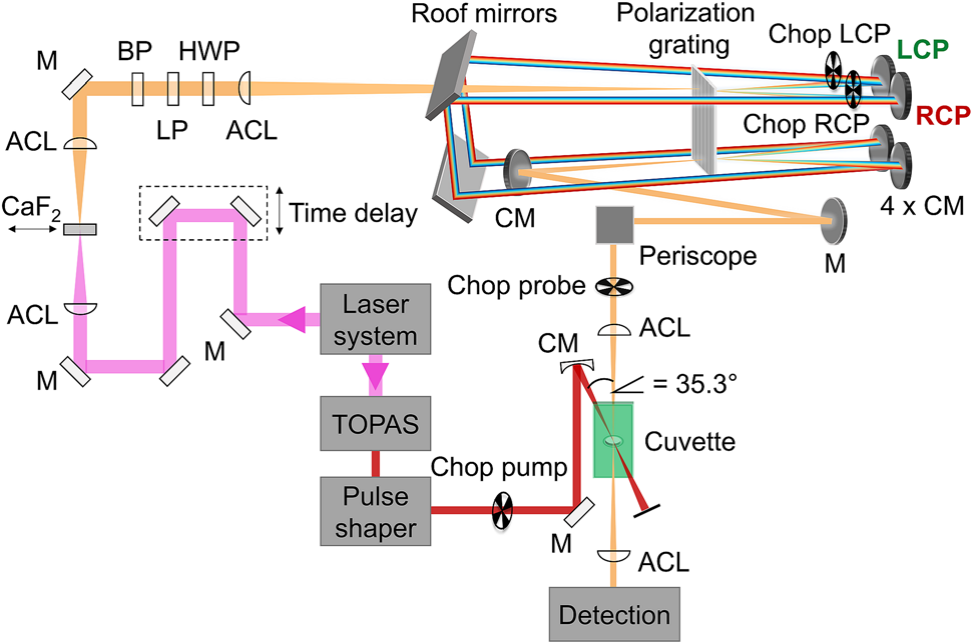
Schematic representation of the experimental setup including the fundamental (pink), which was converted to a white-light continuum (orange) with a continuously moving CaF_2_ plate, and the pump beam (red), which was tuned by a TOPAS and the pulse shaper in wavelength and pulse duration. The pump pulses were linearly polarized parallel to the laser table, passed a chopper and were focused into the sample cuvette under a geometrical magic angle of 35.3° with respect to the probe pulses. Remaining 800 nm light in the probe beam was blocked by a band-pass filter (BP). The white light passed a linear polarizer (LP) and a half-wave plate (HWP) before it was focused into the polarization grating (PG) with an achromatic lens (ACL). The positive (negative) first order of the diffracted light was left-(right)-circularly polarized (LCP, RCP) and was reflected off a concave mirror (CM) at the top, two roof mirrors, and again a concave mirror at the bottom before it was focused into the PG a second time. After recombination in the PG the two beams traveled collinearly with opposite circular polarization over a periscope and were focused in the sample cuvette before being detected *via* the spectrometer.

Our setup (Fig. 2) was driven by a regenerative Ti:sapphire amplifier system (Solstice, Spectra-Physics) at a central wavelength of 797 nm and a pulse duration of 100 fs with a repetition rate of 1 kHz. The beam was pointing-stabilized (TEM – 4D Aligna® system, TEM Messtechnik GmbH) and then split into two parts.

One beam was used to generate linearly polarized excitation pulses with a central wavelength at 633 nm in a commercial non-collinear optical parametric amplifier (TOPAS White, Light Conversion). The pulses were compressed to 32 fs with a liquid-crystal-display-(LCD-)based (SLM-S640d, Jenoptik) pulse shaper of our own design. The pulse duration was determined *via* FROG measurements.^66^ At the sample position the pump pulse energy was set to 300 nJ.

The other beam acted as a probe beam and passed a delay line to provide the time delay between pump and probe pulses. The fundamental was focused by an achromatic lens (ACL, *f* = 50 mm) into a linearly moving 5 mm thick CaF_2_ window to generate a white-light continuum from 470 nm to 720 nm (spectra shown in ESI, Section S1, Fig. S1,† green and red lines) which was collimated with a second achromatic lens (*f* = 125 mm). The white-light continuum passed a band-pass filter to eliminate remaining stray light of the fundamental beam. To clean up and to rotate the linear polarization, it passed a linear polarizer (LP) and a half-wave plate (HWP), respectively.

Afterwards an ACL (*f* = 300 mm) focused the white light into a polarization grating (PG, ImagineOptix; Edmund optics, 12-678). The polarization grating had a size of 25 mm × 25 mm × 0.45 mm with a VIS coating optimized from 450 nm to 650 nm and 286 grooves per mm on a substrate D263. The zeroth order of the polarization grating contained less than 4% of the beam and was blocked afterwards. The first orders were diffracted under an angle of 10° at 550 nm. The positive first order was left-circularly polarized (LCP) and the negative first order was right-circularly polarized (RCP).^67^ The efficiency of diffraction was dependent on the incoming polarization.^68^ Therefore it was necessary to adjust the HWP in a way that the spectra of the first diffraction orders were identical in intensity and shape. Each of the diffracted beams hit a concave mirror (CM, *f* = 100 mm) at the top of a home-built four-mirror mount (ESI, Section S2, Fig. S4†) to collimate them. These mirrors were mounted on the home-built four-mirror mount under an angle of 5° compared to normal incidence to compensate the diffraction angle of the PG. The circularly polarized beams traveled parallel to the incoming beam backwards onto two roof mirrors to change the height and the direction of the beams so that they hit again two concave mirrors mounted on the home-built four-mirror mount at the bottom. Focusing the beams into the PG a second time led to a combined single beam including pulses with opposite circular polarization states that were collimated by a CM and directed into the sample cuvette with a periscope to keep the circular polarization pure.^54^

The pump and the probe beam were focused into the sample cuvette under a geometrical angle of 35.3° to avoid any anisotropy effects. By choosing the linear polarization of the pump pulse in the plane defined by the propagation vectors of the pump and probe beams, a magic angle of 90° − 35.3° = 54.7° resulted.^13,64^

The sample consisted of squaraine polymers which were dissolved in acetone and the concentration was set to an optical density in the linear absorption of approximately 0.4 at the pump wavelength of 633 nm (see Section 4.1) with a cuvette thickness of 0.5 mm. The sample was pumped through a flow cuvette (Starna Scientific, 48/UTWA2 Q) with ultrathin windows with the help of a micro annular gear pump (HNP Mikrosysteme, mzr-2942).

### 3.2 Optical characterization

We measured the ellipticity of the probe beams as well as the beam profiles and pulse durations of pump and probe beams to specify the quality of the measurement beams. The exact description of ellipticity measurement procedure and subsequent calculation of the data points is described in ref. 54 as well as in the ESI in Section S1.† We received the dotted red and dotted green curves in Fig. S1 (ESI, Section S1†) for the ellipticities, *i.e.*, polarization states, of the right- and left-circularly polarized beams, respectively. There is only minor deviation from perfect circular polarization (blue line in Fig. S1†) in the region of interest (550–720 nm). This deviation has no negative effect on the TRCD data as long as left- and right-circular polarization differ symmetrically from perfect circular polarization as shown in our case in the ESI, Section S5,† by using a polarization grating. This grating is optimized for a spectral region from 450–650 nm but can be used in the region 650–720 nm as well.

Spatial characterizations of the beams were carried out with the help of a laser beam analyzer (Ophir®-Spiricon®, Model SP928), *i.e.*, a CCD camera and the software BeamGage®. We measured the shape of pump and probe beams at the position of the center of the cuvette and optimized their spatial overlap. The beam profiles of LCP probe and RCP probe are shown in the ESI, Section S1, Fig. S2a and b,† respectively. The cross-section at an intensity of 
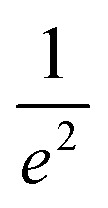
 is marked with the white line inside the rainbow plot and is depicted in green on top and orange on the side of each beam profile plot. The size of LCP probe was 71.8 μm × 64.8 μm with the long axis oriented in *x*-direction in contrast to the RCP probe with a size of 65.0 μm × 71.1 μm and the short axis oriented along the *x*-axis. The pump beam had more than three times the size of LCP probe or RCP probe in *x*-direction and six times in *y*-direction (Fig. S2d†). The spatial overlap of all three beams is shown in Fig. S2c.†

The pulse duration of the pump beam was measured *via* FROG as mentioned before. We measured TA of pure acetone to estimate the temporal resolution of the probe beam and to correct the data for the probe chirp. The probe chirp of around 4.5 ps (see ESI, Fig. S3a†) was mainly caused by the achromatic lenses (ACL) in the beam path (see Fig. 2) as well as the generation of a white-light continuum itself. We applied a well-known procedure^69,70^ to correct the data for the probe chirp. For this we fit the dispersion of the coherent artifact with a fourth-order polynomial and used the resulting coefficients to correct the chirp in the TA data of acetone (Fig. S3b†) and for every other dataset shown in this work. To estimate the temporal resolution, we fit the coherent artifact with the sum of a Gaussian function and its first and second derivative. This procedure is shown exemplary at a single probe wavelength of 576 nm in Fig. S3c.† The FWHM had a maximum value of ≈260 fs at 470 nm and a minimum of ≈162 fs at 700 nm (Fig. S3d,† blue line). The mean FWHM and therefore the temporal resolution of the experiment determined by the probe beam was 206 fs (Fig. S3d,† purple line).

### 3.3 Data acquisition

**Fig. 3:**
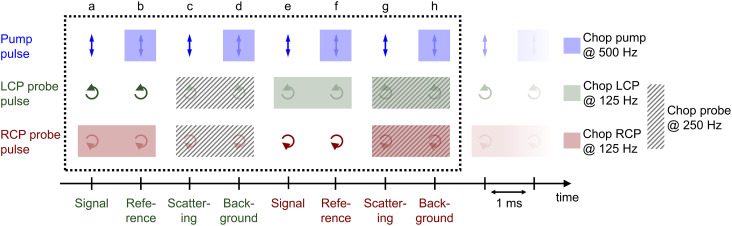
Pulse sequence which represents the procedure of the data acquisition during a TRCD measurement. The scheme represents eight consecutive laser shots (*a*–*h*) which were added together as described in the text. The arrows indicate the polarization direction of the corresponding pulse, *i.e.*, linear polarization of the pump pulses (blue), left- and right-circular polarization of the LCP (green) and RCP (red) probe pulses, respectively. Rectangles with the same color code symbolize the pulses blocked by a chopper in each beam path. The diagonal grey lines depict the pulses blocked by the additional chopper, which was placed in the common beam path of both probe pulses. See Section 3.3 for further details. Design adapted from ref. 71.

The procedure of the data acquisition with a periodically repetitive sequence of eight pulses is represented in Fig. 3. The pump pulses were chopped with a frequency of 500 Hz, *i.e.*, every second pulse was blocked (Fig. 3, blue, top row). We placed a chopper in each of the probe beams (see Fig. 2, Chop LCP and Chop RCP) close to the upper mirrors in the home-built four-mirror mount and set their frequencies to 125 Hz to block four consecutive pulses in each of the beams and let four pulses pass (Fig. 3, green and red in second and third row, respectively). The phase between the choppers in the LCP (Fig. 3, green, second row) and RCP (Fig. 3, red, third row) pulse trains was shifted by two pulses. More precisely, the pulses *a*–*d* of the LCP beam were transmitted and the following four pulses *e*–*h* were blocked, whereas in the RCP beam the pulses *c*–*f* were transmitted and pulses *a*, *b*, *g* and *h* were blocked (Fig. 3, second and third row, green and red rectangles, respectively). A fourth chopper was placed after the periscope (Chop probe, Fig. 2) to increase the signal-to-noise ratio by taking the pump scatter light and the background noise into account. This chopper ran with a frequency of 250 Hz, *i.e.*, two pulses went through and the following two were blocked. This is symbolized in Fig. 3 with diagonal grey lines in LCP and RCP pulse trains (pulse numbers *c*, *d*, *g* and *h*). The TRCD signal was then calculated, using the nomenclature of the laser shots in Fig. 3, as77
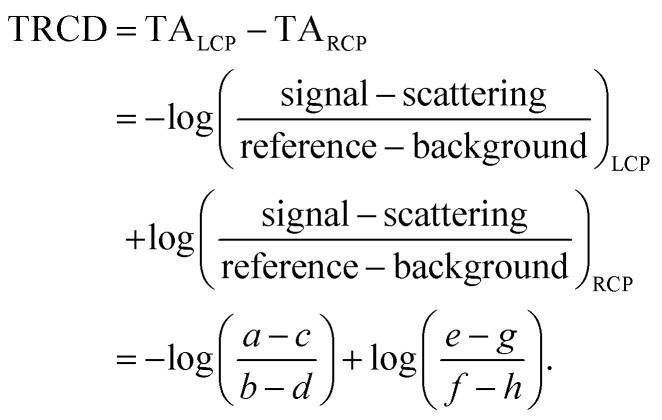


The TA data were calculated by the average of TA_LCP_ and TA_RCP_:78
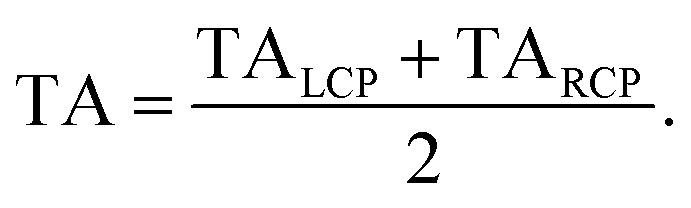


**Fig. 4:**
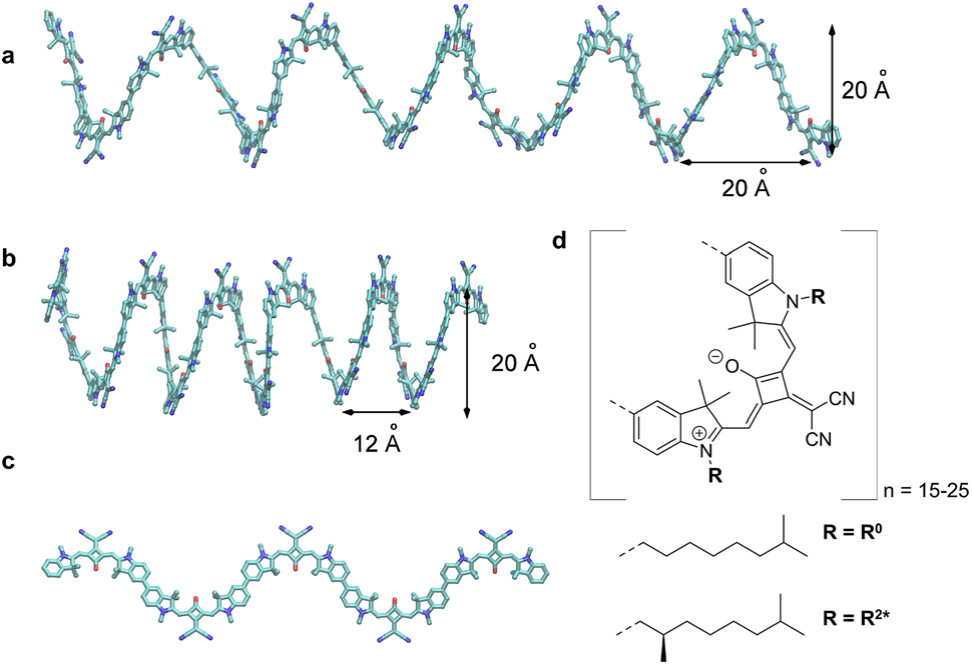
(a) Original helix model.^72^ (b) Squeezed helix model proposed in the present work. (c) Part (*N* = 5) of the planar zigzag structure used as a starting structure to create the random coil conformations, needed to explain the linear absorption spectrum (Fig. 8d). (d) Molecular structure of the squaraine polymer with *n* monomer units. If the achiral residue R^0^ is attached, the polymer forms helices oriented in both directions, *i.e.*, it is a racemic mixture. Adding the chiral alkyl chain R^2*^ to the polymer leads to a left-handed helix.

Each time step consisted of 4000 averaged shots, specifically 2000 averaged shots of the LCP probe and 2000 averaged shots of the RCP probe. We measured 300 time steps which were separated as follows: from −3.5 ps to 2 ps we used a step size of 40 fs steps (138 steps). The following 162 time steps increased exponentially until a time delay of 500 ps. This procedure was repeated 23 times for the chiral squaraine polymer p(SQ-R^2*^) (Fig. 4d), *i.e.*, the whole measurement took 9 h. The achiral p(SQ-R^0^) was used to improve the alignment and to validate our method, *i.e.*, to ensure that we observe no TRCD signal from an achiral sample. This measurement consisted of three averaged maps and is shown in the ESI, Section S3, Fig. S5.†

## 4 Experimental data

### 4.1 Steady-state data

**Fig. 5:**
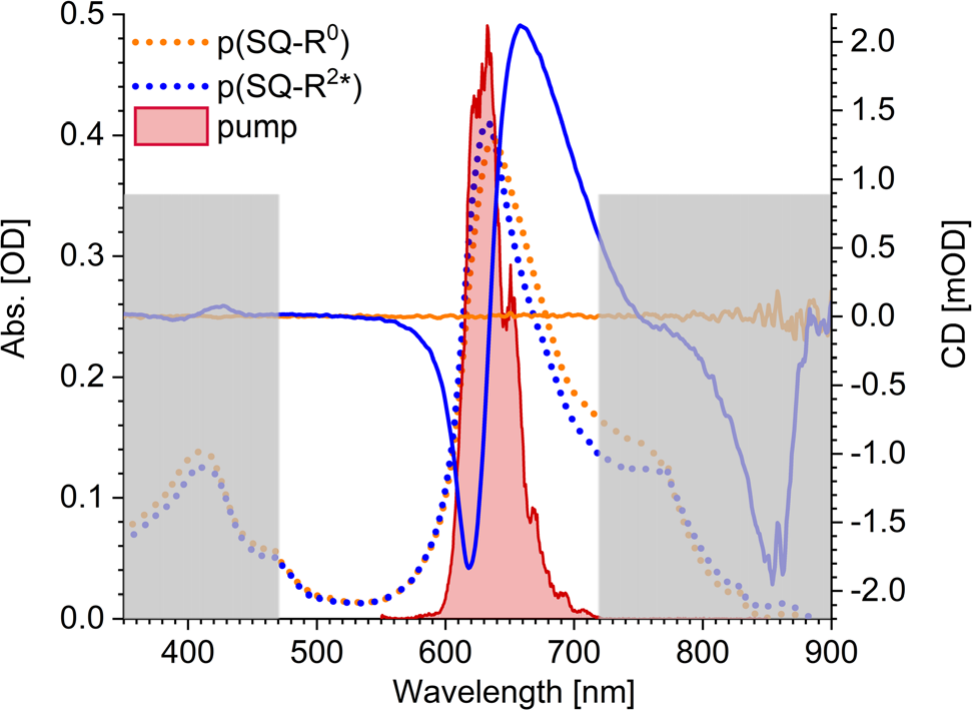
Absorption properties of the squaraine polymers dissolved in acetone. Absorbance (dotted lines) and CD (solid lines) spectra for chiral (blue) and achiral (orange) residue as well as the pump laser spectrum (red shaded area), centered at 633 nm. The grey rectangles mark the region which is not covered by our white-light spectrum, *i.e.*, which we do not investigate within our TA and TRCD setup. The cuvette thickness was 0.5 mm and the concentration was set to 9.88 × 10^−5^ mol l^−1^.

The squaraine polymer in acetone investigated in the present study (Fig. 4) consists of *n* = 15–25 monomer units. The squaraine indolenine building blocks (Fig. 4d) are substituted by an alkyl chain R = R^0^ or a chiral alkyl chain R^2*^. In case of R = R^0^ an overall achiral sample is obtained, which is CD-silent (Fig. 5, orange solid line). The polymer with R = R^2*^ exhibits a negative Cotton effect in the CD spectrum (Fig. 5, blue solid line). The absorption spectra of both polymers (dotted lines) look similar with a high-energetic peak around 410 nm and the main absorption peak at 632 nm in case of p(SQ-R^2*^) and at 637 nm for p(SQ-R^0^). These absorption maxima are strongly blue-shifted when compared to the maximum of the isolated chromophore at 681 nm in acetone,^56^ suggesting H-aggregate-type behavior, as the polymer is formed by arranging the chromophores in a helix. From a systematic study of squaraine oligomers of defined lengths (monomer up to nonamer), including temperature-dependent absorption, NMR spectroscopy, small-angle neutron scattering (SANS) as well as semi-empirical quantum chemical calculations and molecular dynamics simulations in explicit solvent, a structural model for the helix was proposed,^72^ termed “original helix” in the present work. The original helix is characterized by a helix radius of approximately 10 Å, 2.7 squaraine chromophores per helix turn, and a helix pitch of about 20 Å (Fig. 4a).

A left-handed screw sense of the helix could be determined, based on CD measurements on squaraine polymer helices with a chiral residue (R = R^2*^ in Fig. 4d).^9^ Interestingly, a strong low-energy maximum around 850 nm appears in the CD spectrum that is barely visible in the linear absorption spectrum (Fig. 5). As will be shown in the present work, the small low-energy peak in absorption and the strong rotational strength of this peak in circular dichroism can only be explained by increasing the H-aggregate character of the polymer, which in the present work was achieved by a squeezed helix model (Fig. 4b).

### 4.2 Time-resolved data

The pump pulse (Fig. 5, red shaded area) in the time-resolved experiments is centered around the main absorption peak. In the low-energy region a shoulder with ≈0.25 of the intensity of the maximum peak is present in the absorption data. This results from the formation of random coils (Fig. 4c, Section 5.1) within the sample and seems to be more present in the achiral solution. However, our white light ranges from 470 nm to 720 nm and does not cover the mentioned peaks outside this region, as indicated by the grey-shaded area in Fig. 5.

**Fig. 6:**
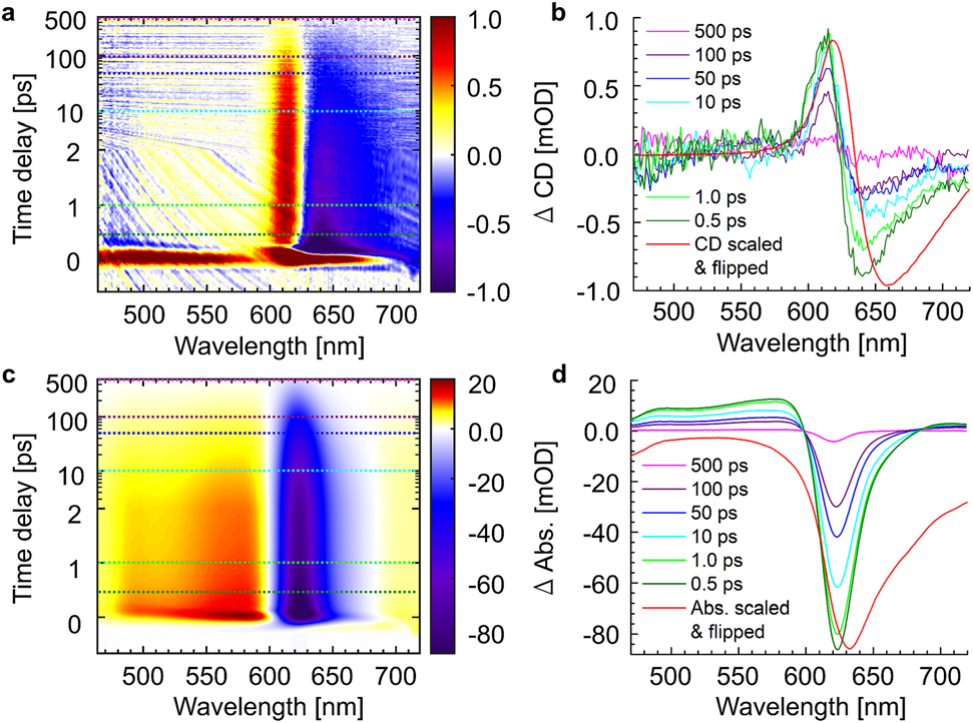
Time-resolved data of the chiral squaraine polymer p(SQ-R^2*^), which was dissolved in acetone and pumped at an excitation wavelength of 633 nm. (a and c) TRCD (a) and TA data (c) plotted linearly up to 2 ps and exponentially afterwards. Dotted horizontal lines (green to purple) symbolize the temporal position of (b and d) individual TRCD (b) and TA spectra (d) for specific time delays as indicated. The time-resolved spectra are plotted together with sign-flipped and scaled steady-state CD and absorption spectra (red lines), respectively.

The whole dataset of the TRCD measurement is depicted in Fig. 6a for the chiral polymer and some selected spectra for particular time delays in Fig. 6b for comparison with the steady-state data (red lines); similarly, the TA data are shown in Fig. 6c and selected spectra in Fig. 6d. The TA map (Fig. 6c) shows a broad ESA from 480 nm to 600 nm. The GSB covers the region from 600 nm to 680 nm and overlaps on the red side of the spectrum with another ESA. This becomes clearer if one compares the flipped-sign steady-state absorption spectrum (Fig. 6d, red line) with selected spectra at several delay times (Fig. 6d, green to purple lines): the steady-state absorption spectrum is significantly wider, pointing at the overlapping ESA for the time-resolved data. When comparing the flipped-sign steady-state CD spectrum (Fig. 6b, red line) with selected spectra at specific delay times (Fig. 6b, green to purple lines), the same behavior is visible: the GSB signal is shifted to the blue and covered by an ESA signal on the low-energetic side. The linear CD spectrum is again broader than the time-resolved spectra. There are two possible reasons why there is no signal in the TRCD data from 470 nm to 600 nm: either the ESA signal in the TA is too small so that it no longer appears in the subtraction or the ESA signal in the TA is achiral and therefore not visible in the TRCD measurement.

To check the correctness of our experiments, the well-known procedure in intrinsic CD would be to measure the other enantiomer and the racemate in the same way. While the synthesis of the enantiomeric monomer is possible, the preparation of the enantiomeric polymer is highly challenging. Namely, the length distribution of the enantiomeric polymers must be exactly the same, which is practically impossible to achieve. Therefore, a polymer p(SQ-R^0^) with an achiral side chain and similar linear absorption was used as a reference, *i.e.*, it should not show any signal in an ideal TRCD measurement. We used this sample to make tiny alignment improvements while simultaneously minimizing the TRCD signal. We could not remove the signal completely as can be seen in the data in ESI, Section S3, Fig. S5a and b.† However, the remaining signal was rather small compared to the TRCD signal of the chiral squaraine polymer (Fig. 6a and b). We can only assume that these signals arise as a consequence of slight deviations from perfect alignment, but we can simply subtract them from the chiral dataset and get the data in ESI, Section S3, Fig. S5e and f.† By comparing these with the TRCD data of p(SQ-R^2*^) it is clear that the main features still remain: the small blue shift of the time-resolved data compared to the steady-state CD spectrum (red line) and the shape are the same. Also the dynamics are the same; this becomes clear by comparing the time constants of the global fits, to be discussed below, in Table 1, which are consistent. The main difference between the TRCD data of p(SQ-R^2*^) and the subtracted data is the higher level of noise in the latter, arising from the subtraction of the data of the achiral sample that included only 3 maps (compared to 23 maps from the chiral sample). Thus, we conclude that the TRCD data in Fig. 6a is real and reflects the dynamics in the chiral polymer sample. We have used that dataset for further processing.

**Table 1:** Time constants resulting from global fits of datasets of p(SQ-R^2*^) in acetone shown in Fig. 7a and c and in the ESI, Section S3, Fig. S6b are compared to time constants obtained from TA measurements in DMF previously^55^

Dataset	*τ*_1_	*τ*_2_	*τ*_3_	*τ*_4_
TA (ref. 55)	100 fs	2 ps	8.6 ps	43 ps
TA (this work)	—	814 fs	19.8 ps	171 ps
TRCD (direct)	—	966 fs	—	140 ps
TRCD (achiral data subtracted)	—	1.05 ps	—	202 ps

### 4.3 Global fits

**Fig. 7:**
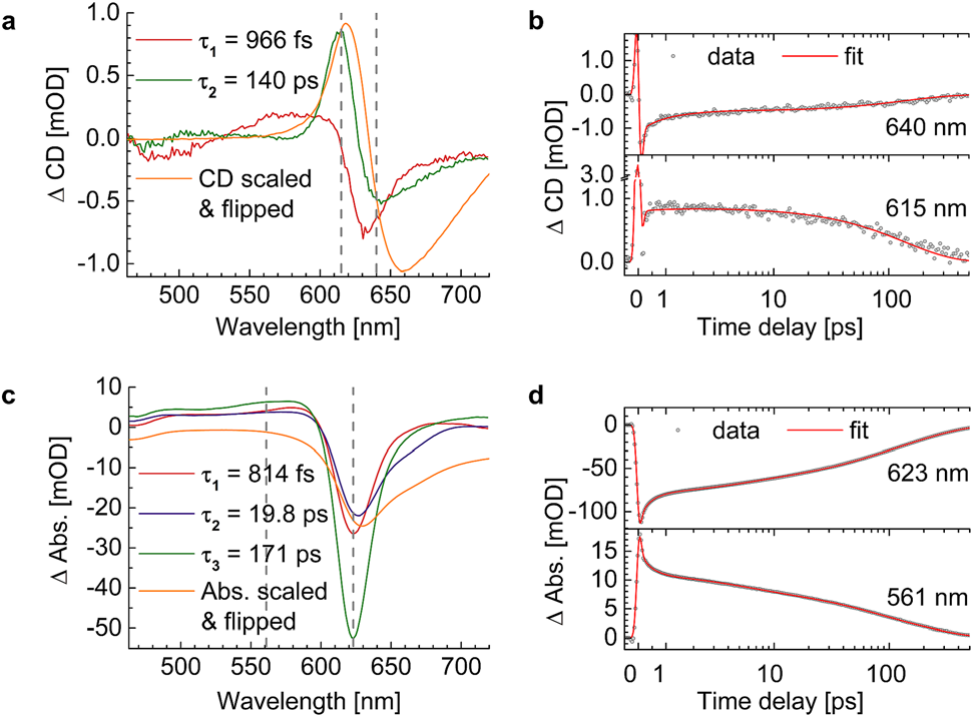
Results from global analysis of the datasets from Fig. 6. (a and b) Decay-associated spectra (DAS) (a) and corresponding data (grey dots) and fit (red lines) of time traces at 640 nm (top) and 615 nm (bottom) of TRCD (b). (c and d) Decay-associated spectra (c) and corresponding data (grey dots) and fit (red lines) at 623 nm (top) and 561 nm (bottom) of TA (d). DAS of TRCD (a) and TA (c) result by using two and three kinetic fit parameters, respectively. Sign-flipped and scaled CD and linear absorption spectra are added in yellow. The vertical dashed grey lines in (a) and (c) indicate the wavelength positions of the data in (b) and (d), respectively.

We evaluated the TA and TRCD data of the chiral polymer *via* global analysis with the software package Glotaran^73^ based on the R-package TIMP.^74^ The results are shown in Fig. 7. To fit the TA data properly, we used three kinetic parameters. In case of the TRCD data we used two kinetic parameters. The fit residuals did not decrease, when adding one more kinetic parameter to each fit and thus we kept the number of kinetic parameters minimal. Within Glotaran the instrument response function is generally fitted with a Gaussian and its first and second derivative including two parameters for the location and the full width at half maximum. While the dataset was chirp-corrected, we took into account any small remaining dispersion with a second-order polynomial fit (see ESI, Section S1, Fig. S3†). We show two exemplary time traces and the corresponding fits of the TRCD and TA data in Fig. 7b and d, respectively. We chose the time traces of the TA data at 623 nm and at 561 nm to investigate the typical GSB and ESA evolution, respectively. For TRCD, we chose time traces located at the minimum and maximum TRCD signals, *i.e.*, at 640 nm and 615 nm, respectively. Those spectral positions are marked in the decay-associated spectra (DAS) in Fig. 7a and c with grey dashed lines. The DAS are plotted together with sign-flipped CD and linear absorption spectra, respectively, to distinguish between GSB and ESA signals.

It is interesting to note that a similar TA study has been performed on the present squaraine polymer but in different solvents, DCM and DMF.^55^ Since the linear optical spectra in DMF are very similar to those in acetone, used in the present study, we expect a similar conformation of the polymer. A direct comparison between the two studies of the linear absorption and CD spectra is shown in the ESI, Section S4, Fig. S7.† Whereas the linear absorption spectra are practically identical, the linear CD spectra are about 15% smaller in amplitude in DMF than in acetone. These differences show the higher sensitivity of CD as compared to absorption with respect to small details of the molecular structure. Despite these differences, it can be expected that the kinetics of excited states is qualitatively similar in acetone and DMF. An advantage of the earlier study is that the TA spectra were measured over a somewhat larger wavelength range, in particular towards longer wavelengths, where a second negative peak was resolved that corresponds to the shoulder in the linear absorption spectrum associated with the random coil conformation of the squaraine polymer, as will be shown below. The TA spectra of the former study pumped at two different wavelengths, assumed to selectively excite the helix and the random coil conformations of the polymer, have been analyzed by a global fit revealing four time constants that are similar for the two different pump wavelengths. The TA time constants following the excitation of the helix are 100 fs, 2 ps, 8.6 ps and 43 ps, which have been interpreted to represent exciton transfer between helix and random coil sections, exciton relaxation within the latter, back transfer from random coil to helix, and relaxation to the electronic ground state, respectively.

An advantage and the major novel feature of the present study is the availability of the TRCD signal that has not been detected previously. The two time constants 966 fs and 140 ps, obtained from the global analysis of the TRCD signal, are in the same order of magnitude as the 2 ps and the 43 ps time constants of the earlier TA analysis. Most likely the 140 ps time constant reflects the transition to the electronic ground state, as before. However, exciton relaxation in the random coil conformation cannot be seen in the TRCD spectrum, and, hence, the interpretation of the 966 fs time constant seen in the TRCD spectrum must be different.

In order to clarify that the TRCD data of p(SQ-R^0^) show only artifacts, we subtracted them from the TRCD dataset of p(SQ-R^2*^). We performed global analysis of the subtracted data shown in the ESI, Section S3, Fig. S6.† The DAS as well as the kinetic traces look similar to the pure TRCD data of p(SQ-R^2*^) and the resulting time constants are consistent (Table 1).

Interestingly, the global analysis of the present TA experiment reveals, besides the two time constants from TRCD, a third time constant of 19.8 ps. It is tempting to assume that this time constant reflects slow energy transfer in the random coil section of the polymer. In order to get deeper insights into the exciton relaxation in the squaraine polymer and how it is reflected in the TA and the TRCD spectra, we present structure-based theoretical studies in the following.

## 5 Application of TRCD theory to experimental data

### 5.1 Structural model and parametrization of the Hamiltonian

The helix model proposed in ref. 72 (Fig. 4a) was used as a starting point to calculate linear absorption and CD spectra. Since in ref. 72 the residues attached to the squaraine chromophores were racemic, no helix screw sense bias was expected and no CD spectra were measured. However, the given handedness in the supporting online information was right-handed. In ref. 9, enantiomerically pure alkyl chains were used as residues, as in the present study, and a left-handed screw sense of the squaraine helix was determined, based on the analysis of the CD spectrum. In the present work, we characterized the squaraine monomer by quantum chemical calculations, using (time-dependent) density functional theory (TDDFT) and the CAM/B3LYP exchange–correlation (XC) functional and a 6-31G** basis set. After geometry optimization, we calculated the charge densities of the ground and first excited state, as well as the transition densities between these states. The electrostatic potential of these charge and transition densities were fitted by assigning atomic partial charges to the atoms.^59^ With these partial charges the electrostatic shifts in local transition energies of the one- and two-exciton states were calculated as well as the excitonic coupling between the transition densities of the chromophores. For this purpose we placed the partial charges onto the atoms of the squaraine chromophores in the helix. The initial atomic coordinates of the latter were taken from the ESI of ref. 72. We inverted the handedness of the helix from right- to left-handed screw sense. In addition, we doubled the helix length from *N* = 8 to *N* = 16 chromophores, in order to describe the polymers considered in the present work.

Since the transition dipole moment of 10.3 D obtained in the quantum chemical calculations agrees with the 10.7 D estimated from the absorption spectrum of the squaraine monomer in acetone,^56^ no rescaling of the atomic transition charges was necessary. We also used unscaled atomic partial charges of the ground and excited states. The numerical values of the different types of atomic partial charges are given in the ESI, Section S15, Table S2.† Initially, a mean transition energy *E*_0_ corresponding to a wavelength of 681 nm was assumed for the squaraine monomers, as obtained from the maximum of the experimental absorption spectrum of the isolated squaraine monomer in acetone.^56^ The intermolecular part of the spectral density *J*(*ω*) of the exciton-vibrational coupling was assumed to be identical with that obtained from fluorescence line-narrowing spectra of the bacteriochlorophyll *a* monomer bound to the B777 complex^65^ that has been widely used. It consists of a rather structureless function peaking at 25 cm^−1^ and decaying to zero for smaller and larger frequencies. It reflects the contributions from intermolecular vibrations which we expect to be similar for different chromophores. An intramolecular part was added with a maximum at 1200 cm^−1^ obtained from the position of the vibrational sideband of the isolated squaraine monomer.^56^ Overall the spectral density reads79
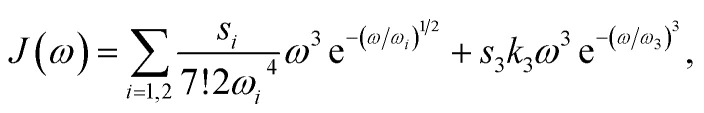
where *k*_3_ = 3(*ω*_3_^4^*Γ*(4/3))^−1^, containing the *Γ*-function, and 7!2*ω*_*i*_^4^ are normalization constants, and the remaining parameters are *s*_1_ = 0.8, *s*_2_ = 0.5, ℏ*ω*_1_ = 0.557 cm^−1^, ℏ*ω*_2_ = 1.936 cm^−1^ for the low-frequency part of the spectral density and ℏ*ω*_3_ = 1200 cm^−1^ for the high-frequency part. Note that the maxima of these different contributions to the spectral density occur at 36*ω*_*i*_ for *i* = 1, 2 and at *ω*_3_ for *i* = 3. Exciton relaxation was found to be sensitive to the amplitude *s*_3_ of the high-frequency component. From comparison with TRCD and TA data a value *s*_3_ = 0.15 was inferred that will be used also in the calculation of linear spectra discussed below.

Disorder in local transition energies was taken into account by randomly assigning site energies from a Gaussian distribution function and averaging the resulting homogeneous spectra over many (10 000) realizations of this disorder. In addition, we took into account disorder in torsional angles between neighboring squaraine monomers by random variation from a Gaussian distribution function. For the different realizations of torsional angles, the excitonic couplings and site energy shifts were calculated and the homogeneous spectra calculated and averaged, taking into account also the additional site energy shifts, described above. The parameters used for the calculations of linear and non-linear spectra, presented below, are summarized in Table 2.

**Table 2:** Summary of parameters^a^ used in the calculations of optical spectra

*E*_0_(sh)	*E*_0_(h,zz)	Δ*E*_inh_	Δ*θ*(h,sh)	Δ*θ*(zz)	*s*_3_	*f*_exc_(sh)	*τ*_anni-2exc_	*τ*_*M*→g_
681 nm	708 nm	1700 cm^−1^	20°	120°	0.15	2.4	30 fs	140 ps

a *E*_0_(sh), *E*_0_(h), *E*_0_(zz): site energies for chromophores in squeezed helix (sh), original helix (h), and zigzag random coil (zz), respectively; Δ*E*_inh_: FWHM of Gaussian distribution function used for the description of static disorder in site energies; Δ*θ*: FWHM of Gaussian distribution function used to describe static disorder of the torsional angles between neighboring chromophores in the helices (h, sh) and the zigzag random coil (zz); *s*_3_: Huang–Rhys factor of the high-energy contribution to the spectral density (eqn (79)); *f*_exc_(sh): enhancement factor of excitonic couplings in the squeezed helix; *τ*_anni-2exc_: implicit dephasing time constant (eqn (73)) used in the ESA lineshape function; *τ*_*M*→g_: time constant for transition between *M*th exciton state and electronic ground state.

### 5.2 Linear absorption and circular dichroism spectra and refinement of structural model

**Fig. 8:**
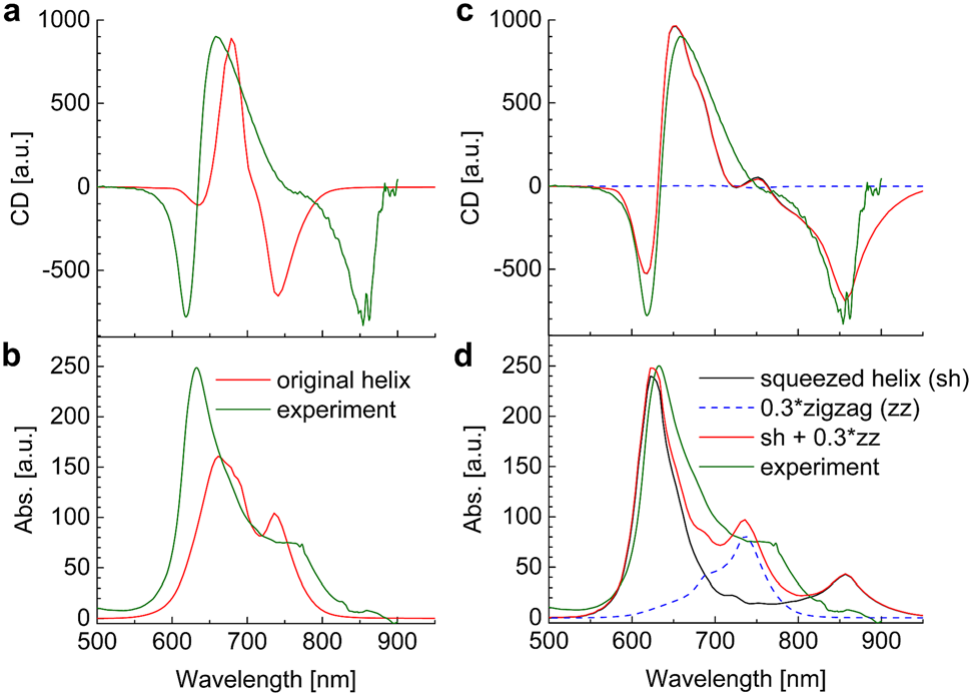
Circular dichroism spectra and absorption spectra calculated for a squaraine helix in the original helix model^72^ in (a) and (b), respectively and in a new model, assuming a 1 : 0.3 mixture of squeezed helices and zigzag random coils in (c) and (d), respectively, are compared to experimental data measured in acetone, *T* = 300 K. The parameters used in the calculations are given in Table 2.

The linear absorption and circular dichroism spectra obtained for the original helix model (Fig. 4a) are compared in Fig. 8 (left) to the experimental data.

Although there is qualitative agreement between calculations and experiments, there are quantitative differences that cannot be explained by uncertainties of the quantum chemical calculations and our theory of optical spectra. Three main differences are: (i) the spread of excitation energies in the experiment is roughly twice larger than in the calculations; (ii) the lowest-energy exciton state observed at 750 nm in the calculations and at 850 nm in the experiment has considerable oscillator strength in the calculated absorption spectrum and almost no intensity in the experiment, where it is much better visible in the CD spectrum (around 850 nm); (iii) the exciton energies range between 600 nm and 750 nm in the calculations and between 600 nm and 850 nm in the experiment. Hence, the center of the exciton band seems to be considerably red-shifted in the experiment, although the experimental transition energy of the squaraine monomer in acetone was assumed as reference site energy *E*_0_.

These differences are reminiscent of the influence of charge transfer (CT) states on site energies and excitonic couplings.^75–78^ The coupling to CT states, however, requires wave function overlap between the chromophores. In order to get closer to this scenario and in order to achieve a more parallel H-aggregate-type orientation of transition dipole moments, we have varied the torsional angles between chromophores and in this way refined the structural model. Since in this squeezed helix (Fig. 4b) the mutual orientation of transition dipole moments is more sandwich-like than in the original model, the J-type low-energy absorption peak loses intensity, in better agreement with the experimental observation. As discussed in ESI, Section S14,† the coupling to CT states can be taken into account implicitly, under certain circumstances (large energies of CT states), by adapting the site energies and excitonic couplings. Here, we have decreased the site energy of the chromophores in the squeezed helix such that the corresponding wavelength is increased by 27 nm from 681 nm (isolated chromophore in acetone, original helix) to 708 nm (squeezed helix). Additionally, we have increased the excitonic couplings by a factor of 2.4. In this way an almost quantitative fit of the experimental CD spectrum can be obtained (Fig. 8c).

However, in the calculated absorption spectrum (Fig. 8d, black) a significant part is still missing, in particular around 750 nm, where there is a clear shoulder in the experiment. It has been demonstrated before that the squaraine helix can be unfolded (*e.g.*, by increasing the temperature).^72^ Assuming that the sample consists of a mixture of unfolded zigzag random coils (Fig. 8d, blue dashed) and folded squeezed helices in a concentration ratio of 0.3 : 1 results in a total absorption spectrum (Fig. 8d, red) that semi-quantitatively agrees with experimental data (Fig. 8d, green). Note that the CD spectrum practically stays the same as without random coils, since the CD spectra of the latter average to zero. The zigzag random coils were obtained from a planar zigzag structure (Fig. 4c), but using *N* = 16 by randomly assigning torsional angles from a Gaussian distribution function with an FWHM of 120°. The latter value has been chosen such that the J-aggregate-type absorption spectrum becomes similar to the absorption spectrum of the present polymer in CHCl_3_ solution (Fig. 6 in ref. 9), where it is assumed to be in a pure zigzag random coil conformation. Finally, we note that the absorption spectrum can be fitted equally well by assuming that the zigzag random coil and helix conformations are covalently connected, *e.g.*, by considering a polymer made of 24 chromophores (instead of 16 considered so far), where the first 19 are in a squeezed helix conformation and the remaining five chromophores constitute the zigzag random coil fraction (Fig. 13). We will come back to this polymer in the calculation of time-resolved spectra. The atomic coordinates of the mean structures of the original helix, the squeezed helix, and the mixed helical/random coil conformation are given in the ESI, Section S15.†

### 5.3 TRCD and TA spectra

We start by investigating the signatures of exciton relaxation in TRCD and TA spectra in general. Previous studies^55^ have revealed ultrafast exciton relaxation in squaraine oligomers with fastest components in the sub-50 fs range that is well below the time resolution of the present experiments. However, as will be shown below, the exciton-state lifetimes increase with decreasing energy, since there are fewer relaxation channels available. For a quantitative evaluation we use the frequency-resolved average inverse lifetime *T*(*ω*)^−1^ defined as^79^80
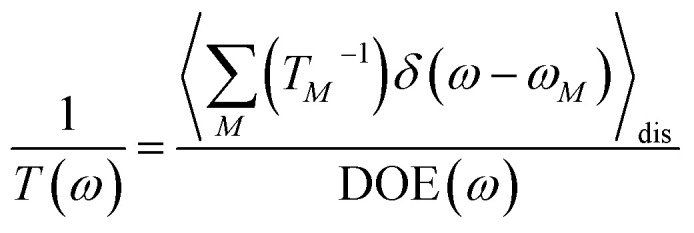
that contains the inverse lifetime of the *M*th exciton state81
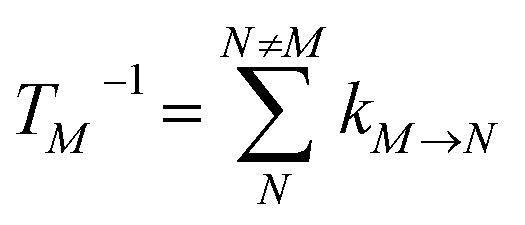
which is determined by the exciton relaxation rate constant *k*_*M*→*N*_ (eqn (31)). The frequency *ω*_*M*_ is given as *E*_*M*_/ℏ, where *E*_*M*_ is the energy of the *M*th eigenstate of *H*^(1)^_exc_ (eqn (2)), and DOE(*ω*) is the density of exciton states,82
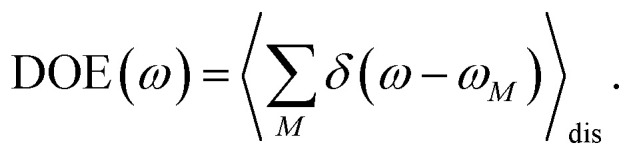


The 〈…〉_dis_ denotes an average over static disorder in site energies of the pigments and torsional angles of the helix. In addition, we will analyze the overall probability distribution of exciton-state lifetimes defined as83
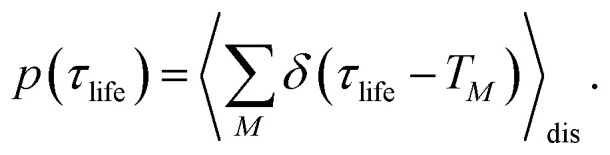


**Fig. 9:**
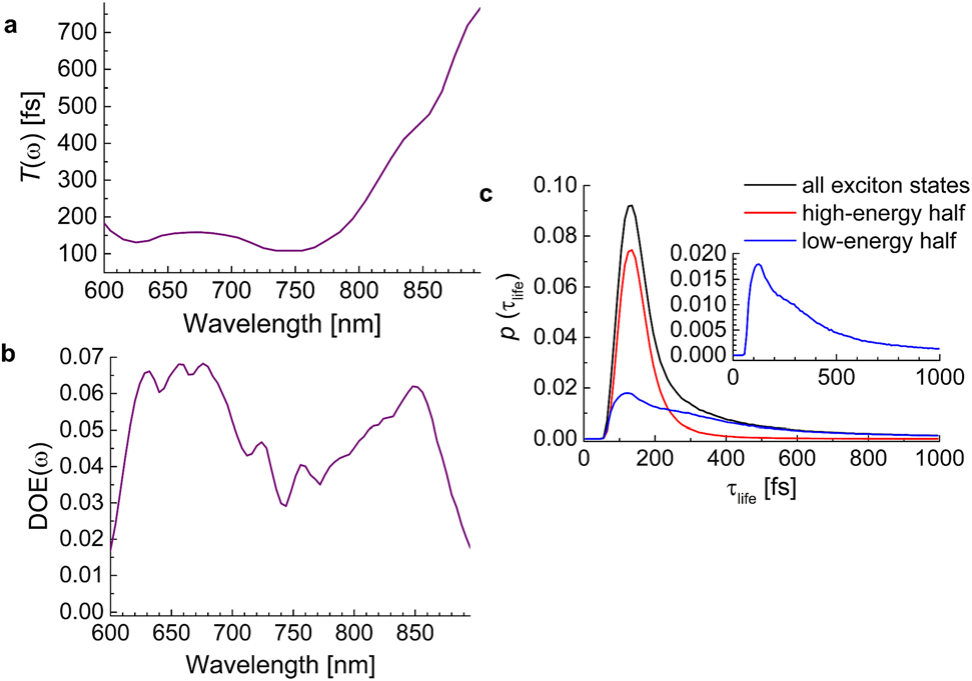
(a) Average frequency-resolved exciton-state lifetime *T*(*ω*) (eqn (80)). (b) Density of exciton states DOE(*ω*) (eqn (82)). (c) Probability distribution of lifetimes (eqn (83)) of all exciton states (black line), high-energy half of exciton states (red line) and low-energy half of exciton states (blue line). The inset contains an enlarged view on the lifetime distribution of the low-energy half of exciton states.

The lifetimes of exciton states of the squeezed helix model, calculated for *s*_3_ = 0.15, are analyzed in Fig. 9. The average lifetime varies between 100 fs and 200 fs for wavelengths 600 nm < *λ* < 750 nm and for longer wavelengths (smaller energies) increases linearly up to 800 fs at *λ* = 900 nm (Fig. 9a). The density of exciton states DOE(*ω*) has a local minimum around 750 nm that divides the high- and the low-energy half of exciton states (Fig. 9b). The high-energy half has a much sharper probability distribution of lifetimes (red line in Fig. 9c) than the low-energy half (blue line). Although the latter also peaks around 100 fs, it has a long tail extending to lifetimes well above 800 fs. Hence, we expect a highly multiexponential exciton relaxation, in particular in the low-energy half of the exciton manifold.

We will investigate now how this exciton relaxation is reflected in the TRCD and TA signals. As in the experiment, we consider excitation by a pump pulse with *τ*_p_ = 30 fs (eqn (11)) centered at 633 nm, where the high-energy exciton states of the squeezed helix are excited.

**Fig. 10:**
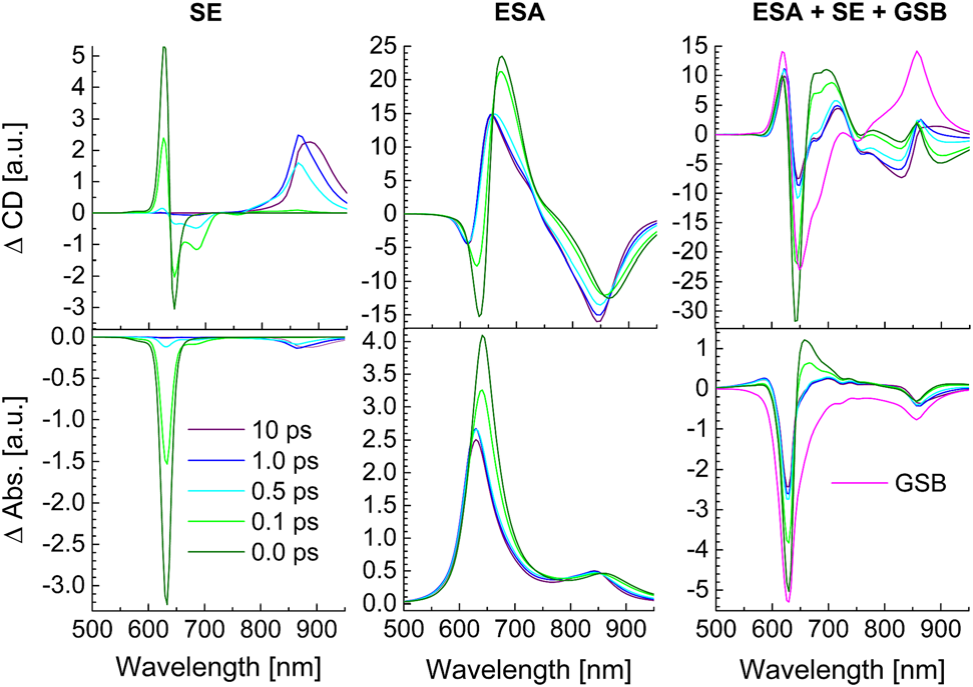
Calculations of TRCD (upper row) and TA (lower row) spectra, obtained for squeezed helices (Fig. 4b) using the parameters in Table 2. The left column shows the SE contribution, the middle column that of ESA and the right column the GSB as well as the complete signals. Excitation by a 30 fs pump pulse centered at 633 nm has been assumed. The delay times between pump and probe pulse are given in the figure legend.

In Fig. 10 the calculated SE and ESA contributions to the TRCD (top) and TA spectra (bottom) as well as the complete spectra are shown for different delay times between pump and probe pulse. The initial population of high-energy exciton states gives rise to a large SE for delay times 0 and 100 fs around 600–650 nm. After 500 fs this signal has practically decayed to zero and the main SE signal is now obtained from the low-energy exciton states around 850–900 nm. Between 500 fs and 1 ps delay the low-energy SE signal in TRCD increases in amplitude and slightly shifts towards longer wavelengths (lower energies). Afterwards the SE signal shifts further to the red between 1 ps and 10 ps delay times. Due to the low oscillator strength of the low-energy exciton states, the SE contribution to the TA signal is much smaller than that to the TRCD signal discussed above. In contrast to SE, the ESA at all delay times contributes over the whole spectrum between 600 and 900 nm, where the low-energy contribution is again larger in TRCD than in TA. There is some similarity between the overall shape of ESA and the corresponding linear absorbance and circular dichroism spectrum (Fig. 8) reflecting the partial bosonic character of the excitons.^80^ This character is disturbed by the fact that every chromophore can be excited only once. Although the strongest changes of the ESA signal occur at wavelengths corresponding to the energies of exciton states that are populated at a given delay time, the lifetimes of high-(low-)energy exciton states can be observed in ESA also at low (high) excitation energies. The overall TRCD and TA signals clearly exhibit the population decay of high-energy exciton states at high energies for short delay times. Whereas the TA signal in the low-energy half of the spectrum is practically constant for all delay times, the low-energy half of the TRCD spectrum shows a complex dynamics reflecting the decay of high-energy exciton states for short delay times ≤500 fs and the arrival of excitation energy in the low-energy half of the exciton manifold for long delay times ≥500 fs.

**Fig. 11:**
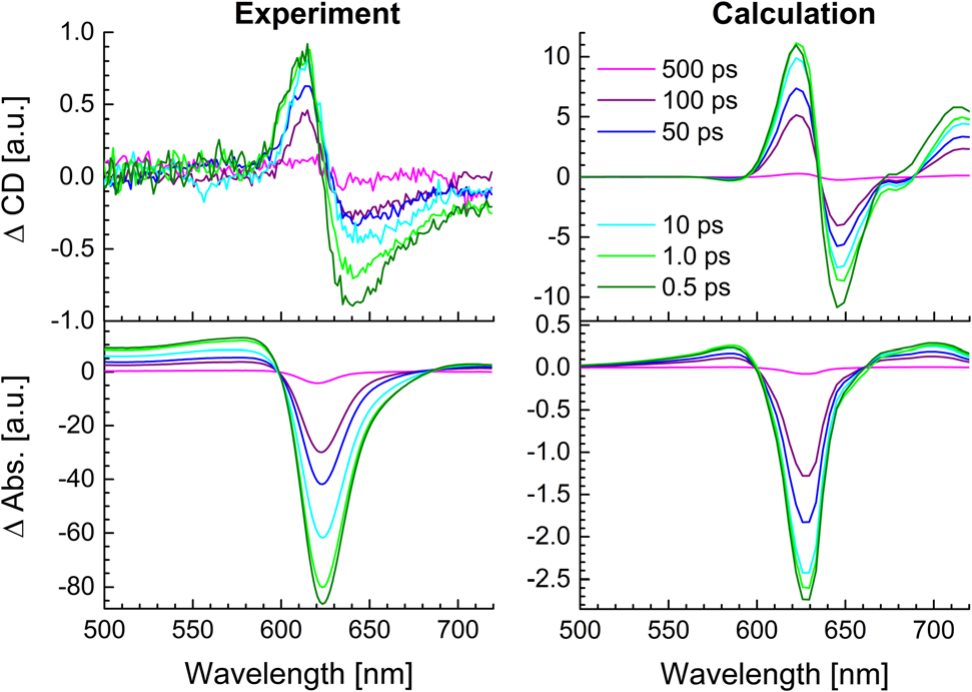
Comparison between experimental (left column) and calculated (right column) TRCD (upper row) and TA (lower row) spectra. The calculations were performed for the squeezed helix (Fig. 4b) using the parameters in Table 2. We assumed an excitation by a 30 fs pump pulse centered at 633 nm. The delay times between pump and probe pulse are given in the figure legend.

In Fig. 11, a comparison is shown between the experimental TRCD (left, top) and TA (left, bottom) spectra with spectra calculated (right) for the squeezed helix. Note that the contribution of the 23% zigzag random coils, inferred from the linear optical spectra (Fig. 8) to the TRCD and TA spectra, is negligible because of the off-resonant excitation wavelength, as shown in ESI, Fig. S12.† Needless to say that the TRCD and TA spectra calculated in the original helix model (ESI, Fig. S13†) give much larger deviations from experimental data than the calculated spectra for the squeezed helix model in Fig. 11.

Qualitative agreement is obtained between theory and experiment in Fig. 11. Between the two smallest delay times of 500 fs and 1 ps the main positive TRCD peak around 625 nm remains constant, whereas the amplitude of the main negative peak around 650 nm in TRCD and around 625 nm in TA reduces. This behavior is explained by analyzing the different contributions to the signals, focusing on a smaller wavelength region (500–720 nm) which is supported by the setup, in Fig. S9.† The ESA contributions to the TRCD signal around 625 nm are constant for the first three delay times, whereas ESA around 650 nm slightly increases for increasing delay times. In addition, SE in TRCD around 650 nm is larger in amplitude than around 625 nm. These differences explain the different temporal behavior of the 625 nm and the 650 nm peak in the TRCD spectrum for early delay times. In case of TA, ESA at 625 nm is constant between 500 fs and 1 ps delay times, and hence the decay of SE explains the decay of the overall TA signal between 500 fs and 1 ps delay times. Between 1 ps and 10 ps delay times, the remaining SE signal has decayed to zero but the change in ESA is much stronger and explains the further decay of the overall TA signal between 1 ps and 10 ps delay times. The decay in the negative peak in the TRCD and the TA spectrum between 1 ps and 10 ps is smaller in the calculations than in the experiment. The GSB contribution to TRCD and TA is practically constant for the first three delay times (the blue solid lines in the right panels in Fig. S9†) and comparison with the overall TRCD and TA signals indicates the strong signatures of ESA, in agreement with the experimental observation that the flipped circular dichroism and linear absorption peaks are broader than the TRCD and TA signals, respectively (Section 4.2, Fig. 6). Note that in the flipped absorption peak also the random coil fraction of the sample contributes that does not contribute to the GSB of the squeezed helix in the TA spectrum in Fig. S9.† The overall decay of the TRCD and TA signals for long delay times ≥50 ps in Fig. 11 simply reflects the decay of excited states to the electronic ground state, assumed to occur with a 140 ps time constant in the calculations.

The present experimental TA spectrum, however, reveals a 19.8 ps time constant not present in TRCD. Noting that the random coil fraction of the sample is only visible in linear absorption but not in CD, it is tempting to assume that the 19.8 ps time constant reflects slow exciton relaxation in random coil fragments. The amplitude of the TA spectrum of random coils is roughly an order of magnitude smaller than that of squeezed helices for the present excitation wavelength of 633 nm. Hence, a direct excitation of the random coil fragments by the present pump pulse can be ruled out, since the pump laser wavelength is off-resonant. Therefore, we would have to assume that after excitation of the helix fraction of the polymer there is fast (sub-ps) transfer to the random coil fraction followed by slow (20 ps) exciton relaxation in the latter. In order to investigate excitation energy transfer between helical and random coil fragments, we have created a mixed configuration of 24 linked chromophores in which the first 19 chromophores adopt a helical configuration and the remaining five chromophores represent the random coil fraction. Interestingly, the resulting frequency-resolved exciton state lifetimes are close to those found for the purely helical fragments (Fig. S10†), with sub-ps lifetimes of all states. Obviously the excitation energy transfer between the different fragments is very efficient. However, we find that random coil fragments still exhibit sub-200 fs exciton-state lifetimes in the high-energy half of the exciton manifold (650 nm < *λ* < 700 nm) and sub-ps lifetimes in the low-energy half (700 nm < *λ* < 750 nm) (Fig. S10†). In order to explain the 20 ps time constant by exciton relaxation/transfer in the random coil segments of the polymer, we would have to assume that the exciton-vibrational coupling is at least an order of magnitude smaller for the random coil fragments than for the helical parts, which is an unrealistic assumption.

In addition, once the exciton has relaxed in the random coil fraction it is transferred back to the helical segment of the polymer, since the latter has the lowest exciton state (seen around 850 nm in the CD spectrum in Fig. 8c). Although our present detection scheme does not allow us to detect SE in TRCD at 850 nm, we should see this back-transfer in the ESA signal in TRCD. However, no such signal is observed. Hence, it can be ruled out that the 19.8 ps time constant seen in the TA spectrum reflects slow exciton relaxation in the random coil fragments. Considering the 30–40 ps time constants for the transition from the excited to the ground state of squaraine polymers in DMF and DCM solvent inferred from previous TA data,^55^ a possibility might be that besides the 140 ps time constant, inferred here, also the 19.8 ps time constant reflects transitions to the electronic ground state. The coupling between exciton and CT states could lead to a stronger displacement of excited-state potential energy surfaces with respect to that of the electronic ground state thereby enhancing Franck–Condon factors relevant for non-radiative transitions to the ground state. The present system indeed does not fluoresce.^9^ Assuming that the helices can be divided in two equal fractions, one with an excited-state lifetime of 140 ps and another with 19.8 ps, leads to a somewhat better description of the TA and TRCD spectra (Fig. S11†). However, such a non-radiative transition to the electronic ground state should be also seen in TRCD, which seems to be in conflict with the global analysis of the experimental data discussed above. Most likely a wider detection range covering wavelengths up to the low-energy maximum of the absorbance of random coil fragments at 750 nm in TA and the lowest exciton state around 850 nm in the TRCD spectrum holds the key to solve this puzzle.

## 6 TRCD application potential and future developments

**Fig. 12:**
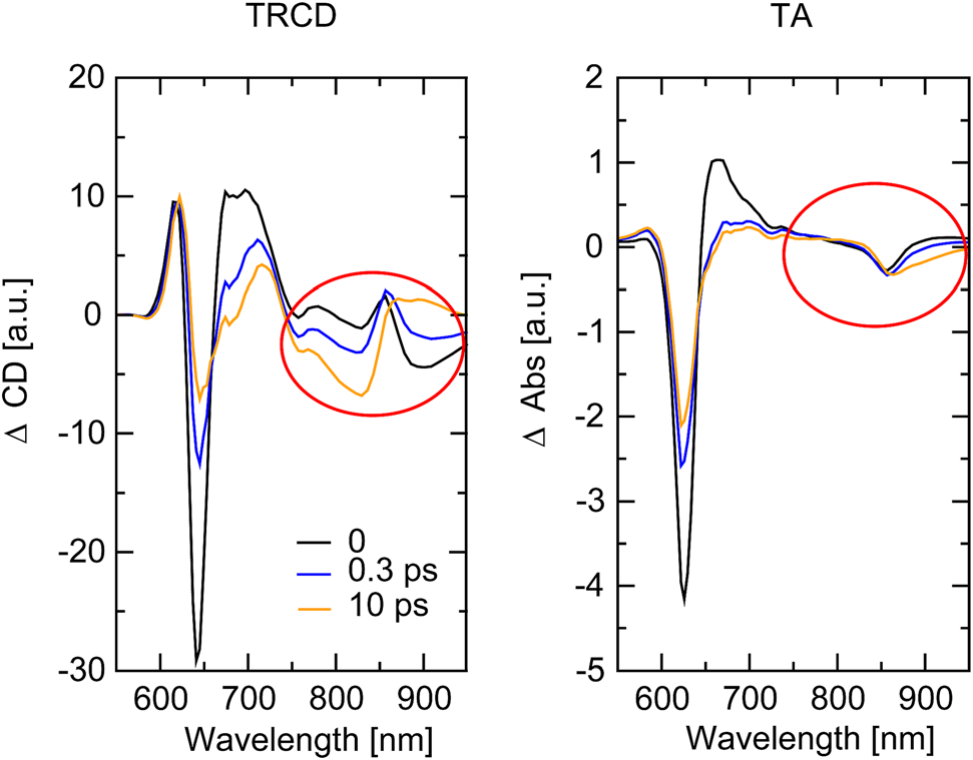
Enlarged view of TRCD (left) and TA (right) spectra calculated for the squeezed helix model for delay times 0 ps (black), 0.3 ps (blue) and 10 ps (yellow) between pump and probe pulse, taken from the right upper and lower panels of Fig. 10. The low-energy region of the spectra is marked with a red ellipse to highlight important differences between TRCD and TA data.

**Fig. 13:**
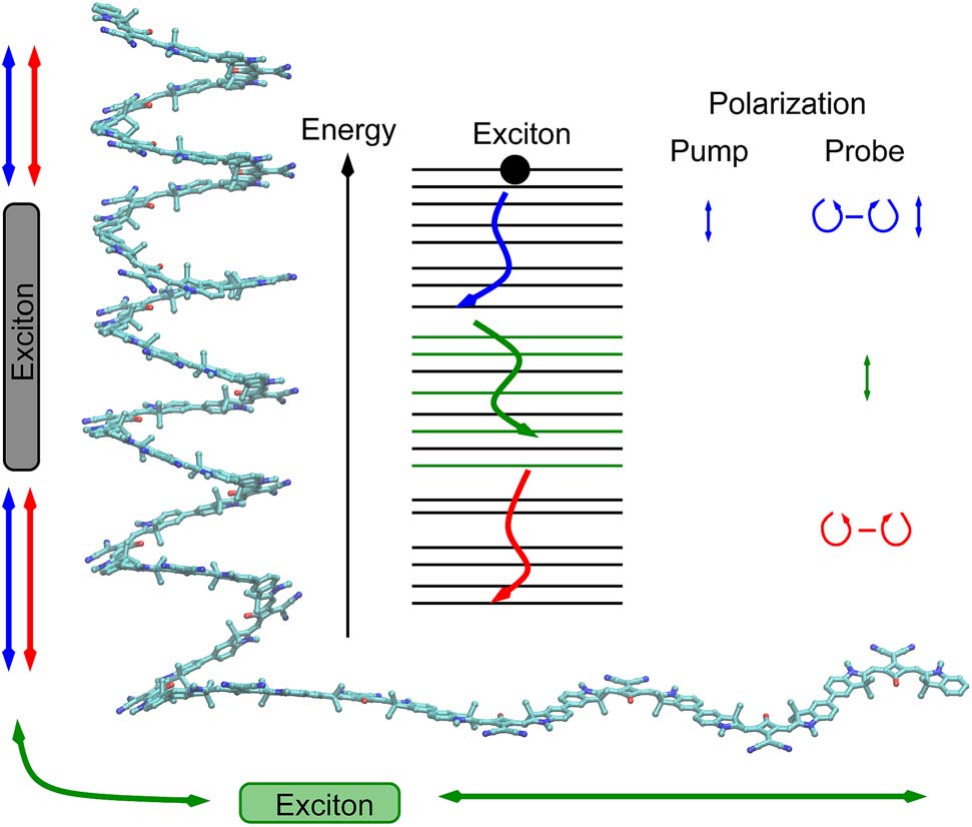
Illustration of exciton relaxation in polymers with mixed helical and zigzag random coil conformation and how it can be probed by circularly and linearly polarized pulses. A linearly polarized pump pulse excites the high-energy exciton states (around 630 nm, blue arrows). The population of these high-energy states can be probed by, both, linearly as well as circularly polarized probe pulses, as in the present experiments. When the exciton relaxes in energy it is spatially transferred into the random coil section of the helix that dominates the exciton states at intermediate energies (around 750 nm, green arrows). The random coil fraction is CD-silent but can be probed with a linearly polarized probe pulse.^55^ Further relaxation brings the exciton back to the helical fraction (with exciton energies corresponding to wavelengths around 850 nm, red arrows) which is practically TA-silent but can be probed with circularly polarized light in TRCD.

From the present calculations of linear absorption and circular dichroism spectra (Fig. 8) it was concluded that the experimental sample consists of a mixture of squaraine polymers with squeezed helix and zig-zag random coil conformations. From the linear spectra, however, it is impossible to decide whether these two conformations can exist in the same polymer, or whether the sample consists of a mixture of polymers in different conformations. This decision can only be made by employing time-resolved spectroscopy using linearly and circularly polarized pulses. In Fig. 13 exciton relaxation in a polymer with mixed conformations is illustrated. Upon excitation of the high-energy exciton states that are located in the helical part of the polymer, the excitation energy is transferred to the random coil section which has a large contribution to exciton states at intermediate energies. The lowest exciton states are again located in the helical part of the polymers, hence there will be back transfer from the random coil to the helical section of the polymer. According to our calculations the low-energy states of the helical fraction is TA-silent, whereas the random coil section has no chirality and, therefore, is CD silent. So far, in our experiments we were able to excite the high-energy exciton states of the helical fraction with a linearly polarized pump pulse and we probed in this high-energy region with linearly and circularly polarized pulses. Obviously, the next step will be to probe the intermediate-energy exciton states with linearly polarized light and the low-energy exciton states with circularly polarized light. The arrival of excitation energy in the low-energy exciton states of the squeezed helix is barely visible in the TA spectrum as a low-energy shoulder of the negative TA peak at 850 nm (Fig. 12, right, region in red ellipse) developing with increasing delay time between pump and probe pulse. In TRCD, in contrast, a strong signal between 750 nm and 950 nm is visible (Fig. 12, left, red ellipse) that even changes sign with increasing delay time. This difference demonstrates the potential of TRCD in providing additional information on exciton relaxation.

In the following we discuss necessary future developments in experiment and theory that will help to establish the full potential of TRCD and to solve the remaining questions concerning the exciton dynamics in the present squaraine polymer.

Experimentally, we will aim at improving the time resolution and extending the spectral detection window towards longer wavelengths. In this way, it should be possible to monitor the complete relaxation of excitation energy in the specific example of the squaraine polymers, in particular its ultrafast start at high energies and its arrival at the lowest exciton state of the helix at 850 nm. The dispersion of the probe pulses can be reduced by replacing the achromatic lenses (ACL), that currently collimate the white-light continuum after its generation as well as the one that focuses the white-light into the grating, by off-axis parabolic mirrors. Such improvements would also be beneficial for studying other supramolecular systems where signals arise that cannot be fully investigated with TA but with TRCD spectroscopy.

Concerning theoretical developments, we aim at a structure-based explanation of the short-range effects and a refinement of the structural model of squaraines. Quantum chemical geometry optimization of the polymer helix is expected to bring the chromophores even closer together. The short-range effects can be studied by applying different diabatization techniques^75,81–83^ to the helix. These techniques would allow us to map the short-range effects onto an effective Frenkel exciton Hamiltonian. Application of such a technique to the central bacteriochlorophyll dimer (known as special pair) of the photosynthetic reaction center of purple bacteria revealed a 50 nm redshift and a two-fold enhancement of excitonic couplings by short-range effects.^75^ These values are in a similar range as those inferred here from comparison of calculated and measured spectra. Based on these quantum chemical calculations, we expect to further improve the description of optical spectra. In case of linear absorption, a tighter helix will further reduce the intensity of the low-energy peak around 850 nm that is barely visible in the experimental absorption spectrum. Concerning the TRCD and TA spectra, we expect to improve the ESA contribution by taking into account the short-range contribution to the bi-exciton shifts *Δ*_*mn*_ that will shift the two-exciton states energetically.

Another improvement of the theory concerns the description of optical lineshapes. The inspection of the absorption spectrum of isolated squaraine chromophores revealed the coupling of the electronic transition to a high-frequency vibration with a vibrational energy of 1200 cm^−1^. So far this mode has been included in the spectral density *J*(*ω*). The Huang–Rhys factor *s*_3_ = 0.15 estimated for this contribution from the exciton relaxation times that in turn were inferred from the time-resolved data, is similar to the value (0.22) estimated from optical spectra.^56^ A more accurate way would be to include the high-frequency mode explicitly in the exciton Hamiltonian.^76–78,84–86^ The lineshape function for ESA can be improved by including a microscopic theory of exciton–exciton annihilation^84^ in order to obtain a state-specific dephasing time constant.

## 7 Conclusions

The study of excitonic systems with considerable conformational freedom such as the present squaraine polymers is a challenging task in many respects: (i) the structure of the excitonic system is unknown. There might exist different conformers with similar free energies. (ii) The optical properties of the excitonic system are very different from those of the isolated chromophore in solution. (iii) A quantum chemical treatment of the excitonic system is difficult and time consuming because of the large number of electronic degrees of freedom and the unknown structure.

In the present work we combined quantum chemical calculations on the isolated squaraine chromophore with a Frenkel exciton model of the excitonic system. Based on the change in optical properties of the excitonic system with respect to the isolated chromophore we inferred two conformational states of the excitonic system, a squeezed helix and a random coil. A 1 : 0.3 mixture of the latter two explains the linear absorption and circular dichroism spectra. This structural hypothesis was tested by transient absorption (TA) and time-resolved circular dichroism (TRCD) spectroscopy, where we selectively excited the squeezed helices. A detailed analysis of these spectra with our Frenkel exciton model suggested that the 1 ps time constant found experimentally reflects the slow part of exciton equilibration in the low-energy half of the exciton manifold. The theory predicts pronounced TRCD and TA signals caused by stimulated emission (SE) from the high-energy exciton states around 600–650 nm at delay times in the 100 fs time range. In addition, strong TRCD signals of the low-energy exciton states around 850 nm were predicted that were barely visible in TA. The experimental TA spectrum revealed a 19.8 ps time constant for which we do not yet have a microscopic explanation. A time constant of 140 ps can be assigned to the decay of excited states to the electronic ground state.

The new aspects of the present work are (i) the introduction of TRCD as a spectroscopic tool to study structure–function relationships of excitonic systems. Prior work using TRCD focused on resolving structural dynamics after optical excitation leading to changes in the chirality properties of the molecule, whereas here we explored TRCD as a probe of exciton dynamics leading to spatial and energetic rearrangements of excitation energy. The generally small signal-to-noise ratio of such a double-difference technique, *i.e.*, obtaining the difference in absorbance between left and right circularly polarized probe pulses with and without a prior pump pulse, is a great challenge. Here we presented a new setup that employs a specific chopping scheme with shot-to-shot detection. Synchronizing four choppers, we measured TA as well as TRCD and corrected the data with respect to scattering and background signals at the same time. Hence, we introduced a working TRCD setup without the need for either a Pockels cell, a photoelastic modulator, a Babinet-Soleil compensator, or a quarter-wave plate to generate fs broadband circularly polarized pulses because we were using a polarization grating. TRCD measurements on an achiral version of the polymer only showed negligible non-zero background signals, resulting probably from slight deviations from a perfectly round beam profile.

(ii) We introduced a Frenkel exciton theory for the description of TRCD spectroscopy of excitonic systems. As opposed to other literature work that deals with structural changes of molecular frameworks after optical excitation and how they affect the TRCD signal, we here explored how a TRCD signal arises even if the molecular framework does not change but only the excitation energy, deposited by the pump pulse, relaxes. The TRCD and the TA experiments are particularly simple to interpret if a magic-angle geometry is chosen between the polarization vector of the linearly polarized pump pulse and the propagation direction of the probe pulse. In this case, the anisotropy of the non-linear signals caused by the pump-pulse excitation averages to zero for isotropic samples. Thereby, also the contributions from electric quadrupole transitions vanish. Application of the model to squaraine helices provided a structure-based explanation of the experimental TA and TRCD data in terms of exciton relaxation and ground-state recovery. In addition, the model predicted a pronounced TRCD signal at low energies (around 850 nm) reflecting the arrival of excitation energy at the lowest exciton state. The small oscillator strength of this state in linear absorption is mirrored in a small amplitude of the SE contribution in TA that makes it nearly impossible to detect the population of the lowest exciton state in TA. Hence, a general complementing feature of TRCD spectroscopy on excitonic systems is that it can detect states that are not visible in TA.

(iii) A new structural model (Fig. 4b) for squaraine aggregates was proposed, in which the chromophores form a helix, with a much smaller pitch than assumed previously (Fig. 4a). For the squeezed helix proposed here, the intensity of the low-energy absorption band is still much larger than in the experiment. We, therefore, hypothesized that an even smaller pitch may be present in the experimental helix. Microscopic modeling of such a structure should provide the basis for a calculation of the couplings between local excited and CT states. In the present calculations these couplings were implicitly taken into account by shifting the site energy of the chromophore in the squeezed helix by 27 nm to the red and by enhancing the interchromophore couplings by a factor of 2.4. So far these changes were inferred from a fit of the optical spectra. A structure-based explanation is still missing.

The understanding of excitation energy and charge transfer in complex molecular systems is an important and challenging problem that can only be solved by a combined effort of theory and experiment. The theoretical and experimental foundation of TRCD spectroscopy presented in the present work provides an additional and in part complementary measure of the exciton dynamics in molecular systems. As an additional observable, it allows one to critically test existing parameterizations of exciton-charge transfer Hamiltonians and dynamical theories of optical spectra and charge- and excitation energy transfer. Complementary to transient absorption, TRCD spectroscopy is capable of visualizing the population of exciton states with a small electric dipole strength.

## Data availability

Additional datasets, formulas and setup verifications supporting this article have been uploaded as part of the ESI.† Further data is available from the corresponding authors upon reasonable request.

## Author contributions

T. R., D. L. and M. H. performed the calculations and analyzed the theoretical data. P. M., J. B. L. and L. R. performed the time-resolved experiments. L. R. analyzed the experimental data. J. S. synthesized the squaraine polymers. L. R. and T. R. wrote the manuscript with input from all co-authors. T. R., T. B. and C. L. supervised the project. All authors read and approved the final version.

## Conflicts of interest

There are no conflicts to declare.

## Supplementary Material

SC-014-D3SC01674A-s001

## References

[cit1] Garab G. , in Biophysical Techniques in Photosynthesis, ed. J. Amesz and A. J. Hoff, Springer Netherlands, Dordrecht, 1996, pp. 11–40

[cit2] von Berlepsch H., Böttcher C., Ouart A., Regenbrecht M., Akari S., Keiderling U., Schnablegger H., Dähne S., Kirstein S. (2000). Langmuir.

[cit3] Didraga C., Klugkist J. A., Knoester J. (2002). J. Phys. Chem. B.

[cit4] Kunsel T., Günther L. M., Köhler J., Jansen T. L. C., Knoester J. (2021). J. Chem. Phys..

[cit5] Eisfeld A., Kniprath R., Briggs J. S. (2007). J. Chem. Phys..

[cit6] Georgakopoulou S., Frese R. N., Johnson E., Koolhaas C., Cogdell R. J., van Grondelle R., van der Zwan G. (2002). Biophys. J..

[cit7] Lindorfer D., Renger T. (2018). J. Phys. Chem. B.

[cit8] Akhtar P., Lindorfer D., Lingvay M., Pawlak K., Zsiros O., Siligardi G., Jávorfi T., Dorogi M., Ughy B., Garab G., Renger T., Lambrev P. H. (2019). J. Phys. Chem. B.

[cit9] Selby J., Holzapfel M., Radacki K., Swain A. K., Braunschweig H., Lambert C. (2022). Macromolecules.

[cit10] Bulheller B. M., Rodger A., Hirst J. D. (2007). Phys. Chem. Chem. Phys..

[cit11] Mukamel S. , Principles of Nonlinear Optical Spectroscopy, Oxford University Press, New York, 1st edn, 1995

[cit12] Valkunas L. , Abramavicius D. and Mančal T., Molecular Excitation Dynamics and Relaxation, Wiley-VCH, Weinheim, 1st edn, 2013

[cit13] Cho M. (2003). J. Chem. Phys..

[cit14] Abramavicius D., Mukamel S. (2006). J. Chem. Phys..

[cit15] Powis I. (2000). J. Chem. Phys..

[cit16] Janssen M. H. M., Powis I. (2014). Phys. Chem. Chem. Phys..

[cit17] Baykusheva D., Zindel D., Svoboda V., Bommeli E., Ochsner M., Tehlar A., Wörner H. J. (2019). Proc. Natl. Acad. Sci. U.S.A..

[cit18] Svoboda V., Ram N. B., Baykusheva D., Zindel D., Waters M. D. J., Spenger B., Ochsner M., Herburger H., Stohner J., Wörner H. J. (2022). Sci. Adv..

[cit19] Cireasa R., Boguslavskiy A. E., Pons B., Wong M. C. H., Descamps D., Petit S., Ruf H., Thiré N., Ferré A., Suarez J., Higuet J., Schmidt B. E., Alharbi A. F., Légaré F., Blanchet V., Fabre B., Patchkovskii S., Smirnova O., Mairesse Y., Bhardwaj V. R. (2015). Nat. Phys..

[cit20] Baykusheva D., Wörner H. J. (2018). Phys. Rev.
X.

[cit21] Comby A., Beaulieu S., Boggio-Pasqua M., Descamps D., Légaré F., Nahon L., Petit S., Pons B., Fabre B., Mairesse Y., Blanchet V. (2016). J. Phys. Chem. Lett..

[cit22] Beauvarlet S., Bloch E., Rajak D., Descamps D., Fabre B., Petit S., Pons B., Mairesse Y., Blanchet V. (2022). Phys. Chem. Chem. Phys..

[cit23] Lux C., Wollenhaupt M., Bolze T., Liang Q., Köhler J., Sarpe C., Baumert T. (2012). Angew. Chem., Int. Ed..

[cit24] Lux C., Wollenhaupt M., Sarpe C., Baumert T. (2015). ChemPhysChem.

[cit25] Böwering N., Lischke T., Schmidtke B., Müller N., Khalil T., Heinzmann U. (2001). Phys. Rev. Lett..

[cit26] Lischke T., Böwering N., Schmidtke B., Müller N., Khalil T., Heinzmann U. (2004). Phys. Rev. A: At., Mol., Opt. Phys..

[cit27] Lewis J. W., Tilton R. F., Einterz C. M., Milder S. J., Kuntz I. D., Kliger D. S. (1985). J. Phys. Chem..

[cit28] Gold J. S., Milder S. J., Lewis J. W., Kliger D. S. (1985). J. Am. Chem. Soc..

[cit29] Xie X., Simon J. D. (1989). Rev. Sci. Instrum..

[cit30] Mesnil H., Schanne-Klein M., Hache F., Alexandre M., Lemercier G., Andraud C. (2001). Chem. Phys. Lett..

[cit31] Fischer P., Hache F. (2005). Chirality.

[cit32] Mesnil H., Hache F. (2000). Phys. Rev. Lett..

[cit33] Dartigalongue T., Hache F. (2005). Chem. Phys. Lett..

[cit34] Dartigalongue T., Hache F. (2006). Chirality.

[cit35] Dartigalongue T., Niezborala C., Hache F. (2007). Phys. Chem. Chem. Phys..

[cit36] Hache F. (2009). J. Photochem. Photobiol., A.

[cit37] Niezborala C., Hache F. (2008). J. Am. Chem. Soc..

[cit38] Schmid M., Martinez-Fernandez L., Markovitsi D., Santoro F., Hache F., Improta R., Changenet P. (2019). J. Phys. Chem. Lett..

[cit39] Mendonça L., Hache F., Changenet-Barret P., Plaza P., Chosrowjan H., Taniguchi S., Imamoto Y. (2013). J. Am. Chem. Soc..

[cit40] Hache F., Changenet P. (2021). Chirality.

[cit41] Mangot L., Taupier G., Romeo M., Boeglin A., Cregut O., Dorkenoo K. D. (2010). Opt. Lett..

[cit42] Trifonov A., Buchvarov I., Lohr A., Würthner F., Fiebig T. (2010). Rev. Sci. Instrum..

[cit43] Meyer-Ilse J., Akimov D., Dietzek B. (2013). Laser Photonics Rev..

[cit44] Hiramatsu K., Nagata T. (2015). J. Chem. Phys..

[cit45] Stadnytskyi V., Orf G. S., Blankenship R. E., Savikhin S. (2018). Rev. Sci. Instrum..

[cit46] Meyer-Ilse J., Akimov D., Dietzek B. (2012). J. Phys. Chem. Lett..

[cit47] Oppermann M., Bauer B., Rossi T., Zinna F., Helbing J., Lacour J., Chergui M. (2019). Optica.

[cit48] Oppermann M., Spekowius J., Bauer B., Pfister R., Chergui M., Helbing J. (2019). J. Phys. Chem. Lett..

[cit49] Kuronuma M., Sato T., Araki Y., Mori T., Sakamoto S., Inoue Y., Ito O., Wada T. (2019). Chem. Lett..

[cit50] Oppermann M., Zinna F., Lacour J., Chergui M. (2022). Nat. Chem..

[cit51] Scholz M., Morgenroth M., Cho M. J., Choi D. H., Oum K., Lenzer T. (2019). J. Phys. Chem. Lett..

[cit52] Morgenroth M., Scholz M., Lenzer T., Oum K. (2020). J. Phys. Chem. C.

[cit53] Morgenroth M., Scholz M., Cho M. J., Choi D. H., Oum K., Lenzer T. (2022). Nat. Commun..

[cit54] Steinbacher A., Hildenbrand H., Schott S., Buback J., Schmid M., Nuernberger P., Brixner T. (2017). Opt. Express.

[cit55] Lambert C., Koch F., Völker S. F., Schmiedel A., Holzapfel M., Humeniuk A., Röhr M. I. S., Mitric R., Brixner T. (2015). J. Am. Chem. Soc..

[cit56] Turkin A., Malý P., Lambert C. (2021). Phys. Chem. Chem. Phys..

[cit57] Nielsen J. T., Kulminskaya N., Bjerring M., Linnanto J. M., Rätsep M., Pedersen M. O., Lambrev P. H., Dorogi M., Garab G., Thomsen K., Jegerschoeld C., Frigaard N.-U., Lindahl M., Nielsen N. C. (2016). Nat. Commun..

[cit58] Adolphs J., Müh F., Madjet M. E.-A., Renger T. (2008). Photosynth. Res..

[cit59] Madjet M. E., Abdurahman A., Renger T. (2006). J. Phys. Chem. B.

[cit60] Raszewski G., Renger T. (2008). J. Am. Chem. Soc..

[cit61] Adolphs J., Renger T. (2006). Biophys. J..

[cit62] Andrews S. S. (2004). J. Chem. Educ..

[cit63] Andrews D. L., Thirunamachandran T. (1977). J. Chem. Phys..

[cit64] Schott S., Steinbacher A., Buback J., Nuernberger P., Brixner T. (2014). J. Phys. B: At., Mol. Opt. Phys..

[cit65] Renger T., Marcus R. A. (2002). J. Chem. Phys..

[cit66] DeLong K. W., Trebino R., Hunter J., White W. E. (1994). J. Opt. Soc. Am. B.

[cit67] Oh C., Escuti M. J. (2007). Phys. Rev. A: At., Mol., Opt. Phys..

[cit68] Oh C., Escuti M. J. (2008). Opt. Lett..

[cit69] Megerle U., Pugliesi I., Schriever C., Sailer C. F., Riedle E. (2009). Appl. Phys. B.

[cit70] Kovalenko S. A., Dobryakov A. L., Ruthmann J., Ernsting N. P. (1999). Phys. Rev. A: At., Mol., Opt. Phys..

[cit71] Steinbacher A. , PhD thesis, Dissertation, Universität Würzburg, 2015

[cit72] Turkin A., Holzapfel M., Agarwal M., Fischermeier D., Mitric R., Schweins R., Gröhn F., Lambert C. (2021). Chem.–Eur. J..

[cit73] Snellenburg J. J., Laptenok S. P., Seger R., Mullen K. M., van Stokkum I. H. M. (2012). J. Stat. Software.

[cit74] Mullen K. M., van Stokkum I. H. M. (2007). J. Stat. Software.

[cit75] Madjet M. E.-A., Müh F., Renger T. (2009). J. Phys. Chem. B.

[cit76] Hestand N. J., Spano F. C. (2018). Chem. Rev..

[cit77] Hestand N. J., Zheng C., Penmetcha A. R., Cona B., Cody J. A., Spano F. C., Collison C. J. (2015). J. Phys. Chem. C.

[cit78] Hart S. M., Banal J. L., Castellanos M. A., Markova L., Vyborna Y., Gorman J., Häner R., Willard A. P., Bathe M., Schlau-Cohen G. S. (2022). Chem. Sci..

[cit79] Klinger A., Lindorfer D., Müh F., Renger T. (2020). J. Chem. Phys..

[cit80] Bednarz M., Knoester J. (2001). J. Phys. Chem. B.

[cit81] Hsu C.-P., You Z.-Q., Chen H.-C. (2008). J. Phys. Chem. C.

[cit82] Voityuk A. A., Rösch N. (2002). J. Chem. Phys..

[cit83] Cupellini L., Caprasecca S., Guido C. A., Müh F., Renger T., Mennucci B. (2018). J. Phys. Chem. Lett..

[cit84] Renger T., May V. (1997). J. Phys. Chem. B.

[cit85] Spano F. C. (2010). Acc. Chem. Res..

[cit86] Friedl C., Renger T., Berlepsch H. v., Ludwig K., Schmidt am Busch M., Megow J. (2016). J. Phys. Chem. C.

