# Supplementary material for: Time-resolved circular dichroism of excitonic systems: theory and experiment on an exemplary squaraine polymer
Source: Chem Sci. 2023 Jul 28;14(35):9328–49. doi: 10.1039/d3sc01674a (PMC10498725; doi:10.1039/d3sc01674a)
Supplement: SC-014-D3SC01674A-s001 [file SC-014-D3SC01674A-s001.pdf]

Electronic Supplementary Material (ESI) for Chemical Science.
This journal is © The Royal Society of Chemistry 2023

Electronic Supplementary Information

Time-Resolved Circular Dichroism of Excitonic Systems: Theory and Experiment on an Exemplary Squaraine Polymer

Lea Ress,[a] Pavel Malý,[a,b] Jann B. Landgraf,[a,f] Dominik Lindorfer,[c] Michael Hofer,[c] Joshua Selby,[d] Christoph Lambert,[d] Thomas Renger,*[c] and Tobias Brixner*[a,e]

[a] *Institut für Physikalische und Theoretische Chemie, Universität Würzburg, Am Hubland, 97074 Würzburg, Germany.*

[b] *Faculty of Mathematics and Physics, Charles University, Ke Karlovu 5, 121 16 Praha 2, Czech Republic.*

[c] *Institut für Theoretische Physik, Johannes Kepler Universität Linz, Altenberger Str. 69, 4040 Linz, Austria.*

[d] *Institut für Organische Chemie, Universität Würzburg, Am Hubland, 97074 Würzburg, Germany.*

[e] *Center for Nanosystems Chemistry (CNC), Universität Würzburg, Theodor-Boveri-Weg, 97074 Würzburg, Germany*

[f] *Freiburg Center for Interactive Materials and Bioinspired Technologies (FIT), Universität Freiburg, Georges-Köhler-Allee 105, 79110 Freiburg, Germany.* ∗ *E-mail: brixner@uni-wuerzburg.de, thomas.renger@jku.at*

S1 Specifications of Pump and Probe Beams

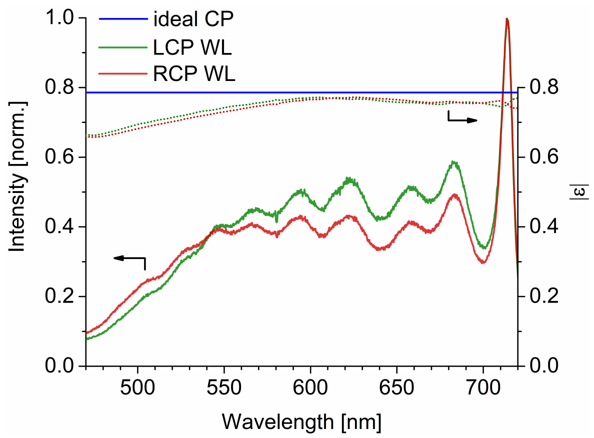

Figure S1: White-light spectrum (solid lines) and ellipticity (dotted lines) of LCP and RCP probe beams in green and red, respectively. The white-light spectra are normalized to the maximum peak at 715 nm. The ellipticity of an ideal circularly polarized beam is depicted in blue with a value of $|\epsilon| = \frac{\pi}{4}$. Ellipticity is depicted as absolute values for both polarizations.

The white-light spectra of the first diffraction orders after the polarization grating (PG) are displayed in Fig. S1. The spectra are normalized to the maximum intensity around 715 nm. As mentioned in the main text, the incoming polarization into the PG affected the efficiency of the diffraction as well as the diffracted spectra.[1] Therefore the optimal position of the half-wave plate (HWP) was determined so that the spectra of the left- and right-circularly polarized probe pulses were equal in shape and intensity. We found that an exact agreement of both spectra was not possible. This is not a crucial problem for time-resolved circular dichroism (TRCD), however, because we detected the intensity of the transmitted light with and without excitation pulse and divided them so that only the difference was left, which is not dependent on the spectral shape anymore. Thus, as long as the ellipticity was the same, a perfect agreement of both spectra was not required.

To measure the ellipticity, each of the two circularly polarized beams was measured separately: Behind the TRCD setup they passed a linear polarizer (LP) and a quarter-wave plate (QWP). The latter was inserted to make sure that possible artifacts from polarization-dependent detection efficiencies of the spectrometer were avoided. The LP and QWP were mounted on a rotational stage and their main axis were oriented in an angle of 45° to each other. Both optics were rotated around 360° in 3° steps and the resulting intensity was measured with the spectrometer.

To determine the absolute handedness of the beams a polarization tester was purchased from Edmund Optics (article number 37-699). Additionally, the properties of the polarization grating itself give an exact determination of the polarization handedness as described in Ref. 1. The polarization state behind the polarization grating of the positive first diffraction order is right circularly polarized and exits the polarization grating to the left side. The negative first diffraction order exits the grating on the right side and is left circularly polarized. Due to the propagation over several optics, the polarization state of the two beams at the cuvette position (see Fig. 2, main text) is mirrored to that behind the polarization grating. The description of LCP and RCP beams, choppers and the calculation of the TRCD signal is related to the polarization state at the cuvette position.

To make sure that all beams hit the sample at the same spot we inserted a camera at the cuvette position and moved it along the longitudinal (z) direction until we were in the focus of the probe beams, i.e., when the beam size was as round and small as possible. We aligned the pump so that the probe beams (Figs. S2a, b) were centered at the intensity maximum of the pump beam, i.e., the upper part of the beam in Fig. S2d. This overlap situation is shown in Fig. S2c with the pump profile as it is shown in Fig. S2d and the LCP probe as green contour and the RCP probe as blue contour on top of it. These blue and green contours are the positions where the intensity of the

probe beams is dropped to $\frac{1}{e^2}$, i.e., only the white line of Figs. S2a and S2b is plotted at scale. The distorted beam profiles of LCP and RCP probe beams had their origin in spatial chirp and a slightly misaligned setup. The fact that the LCP and RCP probe beam profiles were not the same could lead to the assumption that some artifacts in the TRCD map of the achiral squaraine polymer p(SQ-R^0) arose. Since there is no perfect overlap with the pump at the exact same position, one of the probe beams overlaps with another part of the pump beam than the other probe beam.

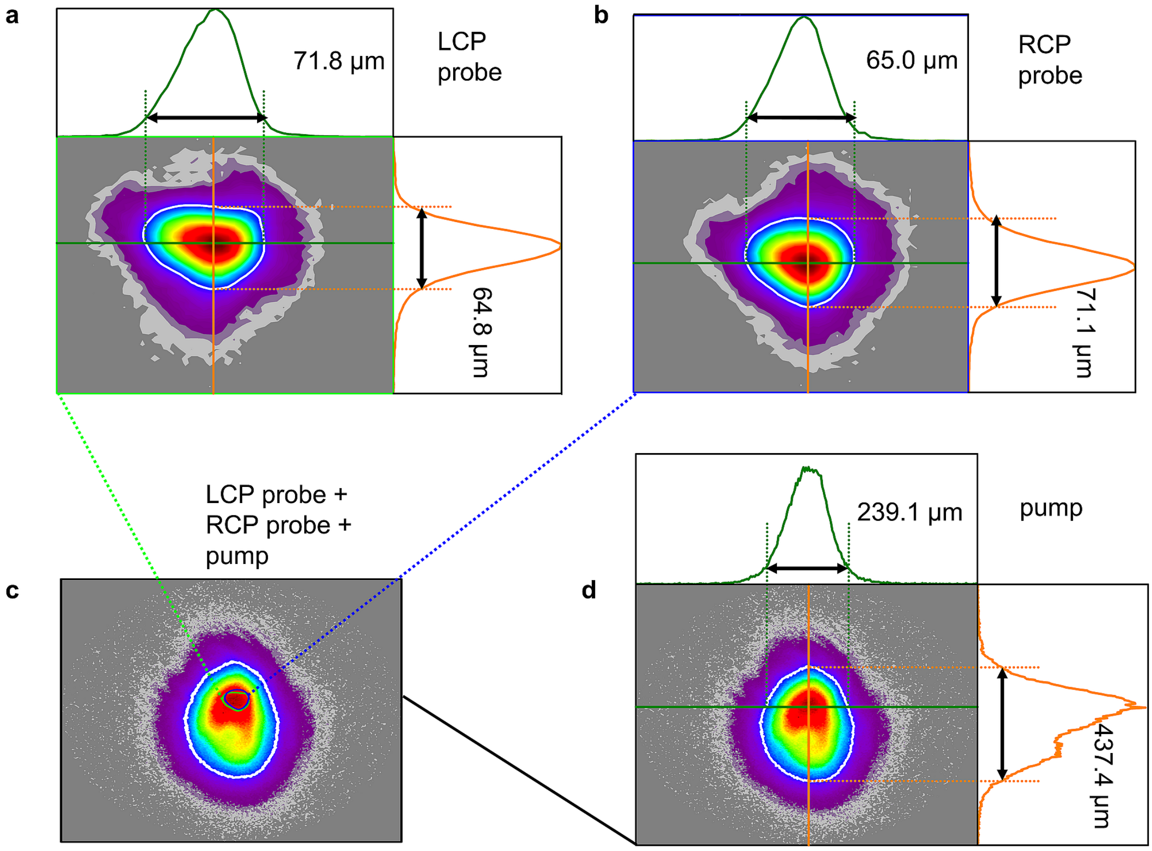

Figure S2: Laser beam profiles of (a) LCP probe, (b) RCP probe, (c) overlap of pump, LCP probe (green) and RCP probe (blue) and (d) pump. The green and orange plots are the cross-sections of the x- and y-axes, respectively. The intensities are normalized to the maximum peak intensity of each plot. The white line in every rainbow plot indicates the intensity drop to $\frac{1}{e^2}$. At this intensity the beam size was measured to the value depicted close to the cross-section plot. Note that the probe beam profiles are enlarged in (a) and (b) but in correct ratio to the pump beam in (c), marked in green and blue for LCP and RCP, respectively.

Another part of evaluating the setup is the estimation of the temporal resolution of the TRCD setup. The procedure is described in the main text in Section 2.2 and the results are shown in Figure S3.

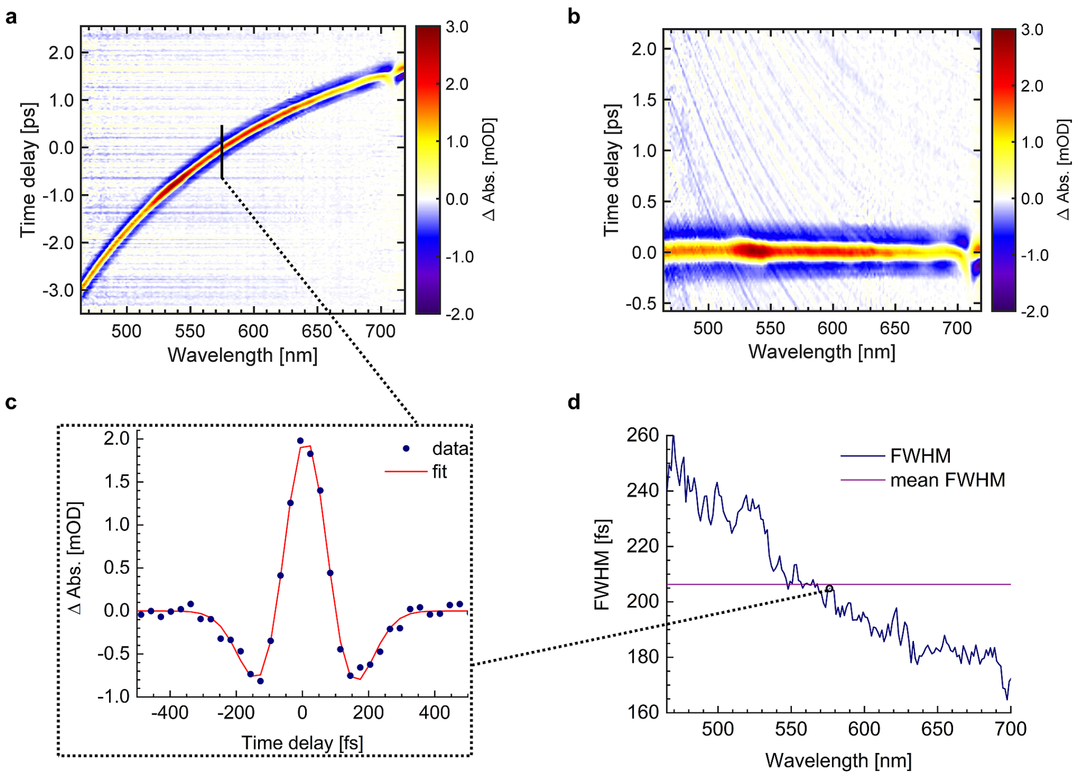

Figure S3: Estimation of the temporal resolution of the setup. (a) TA data of acetone showing the coherent artifact which extends from −3 ps to 1.5 ps. (b) Chirp-corrected TA data of acetone. (c) Exemplary Gaussian fit (red line) of the time trace (blue dots) at 576 nm which is marked with the black line in (a). (d) The temporal resolution was estimated by the fitted Gaussian full width at half maximum (FWHM) as a function of probe wavelength (blue) together with its first and second derivative. The purple line shows the mean FWHM of roughly 206 fs. The black circle marks the FWHM of the fitted time trace at 576 nm in (c).

S2 Home-Built Four-Mirror Mount

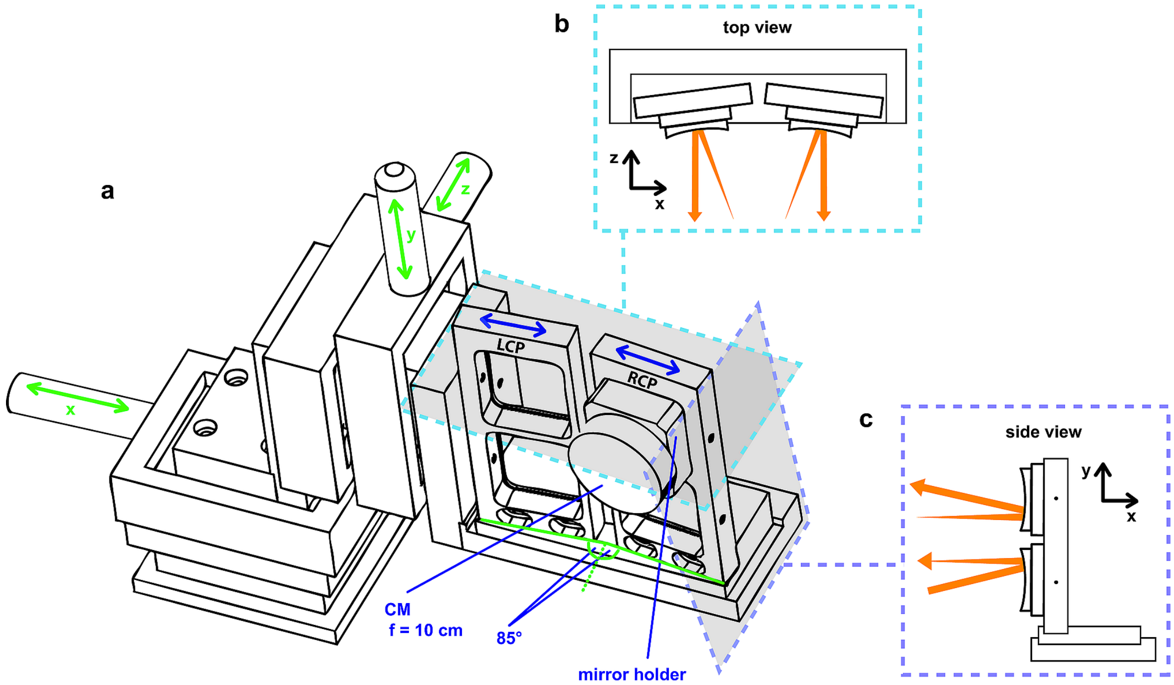

Figure S4: Home-built four-mirror mount with an exemplary beam path from top and side view. (a) Engineering drawing of the four-mirror mount. Green arrows and corresponding letters indicate possible movements of the linear stages in x-, y-, and z-directions with respect to the laser table and the incoming beam traveling along the z-direction. (b) Top view and (c) side view of the four-mirror mount without showing the linear stages. A coordinate system has been drawn for better illustration. The orange lines visualize the laser beams which hit the mirrors directly after diffraction by the PG and before being focused again into the PG.

The home-built four-mirror mount is shown in Fig. S4. The engineering drawing (Fig. S4a) includes three linear stages on the left side. To indicate the moving direction of each linear stage a green arrow and the corresponding direction of the laboratory system was marked on the screw. The laser beam traveled along the z-axis. Attached to the y-axis linear stage was a holder which included two separate rectangular frames marked with LCP and RCP. Each of these frames could be moved in x-direction, which is indicated by the blue arrows. These frames were not mounted under normal incidence to the z-axis but in an angle of 85° to compensate the angle of the white-light beam which was diffracted under an angle of 10° (at 550 nm) by the polarization grating (PG). To collimate the beam after the PG, we inserted two mirror holders (thorlabs, KMSS and MH25T) with a concave spherical mirror (CM) in each holder. For better illustration, one mirror holder and a concave mirror is shown in the upper part of the RCP frame. To demonstrate the beam path after every mirror a top view and a side view is shown in Fig. S4b and c, respectively, together with a coordinate system. In the top view one sees the upper two mirrors of each frame. The orange lines symbolize the laser beams which spread from the PG to the mirrors under an angle of 10°. After passing the concave mirror the beam was collimated and due to the tilt of the frame the beam was reflected back parallel to the z-direction of the laboratory system. In the side view only the RCP frame is visible. The incoming laser beam hit the upper concave mirror in the lower edge of the mirror. This led to a reflection upwards in the direction of the roof (see Fig. 2, main text for full beam path). After passing the roof mirrors the beam traveled back under an angle from the bottom to the lower mirror in the frame. This concave mirror (f = 10 cm) was hit on the top edge, and therefore the beam was parallelized to the table again and focused into the PG.

S3 Reference Data of Achiral Squaraine Polymer p(SQ-R^0) and Subtracted Data

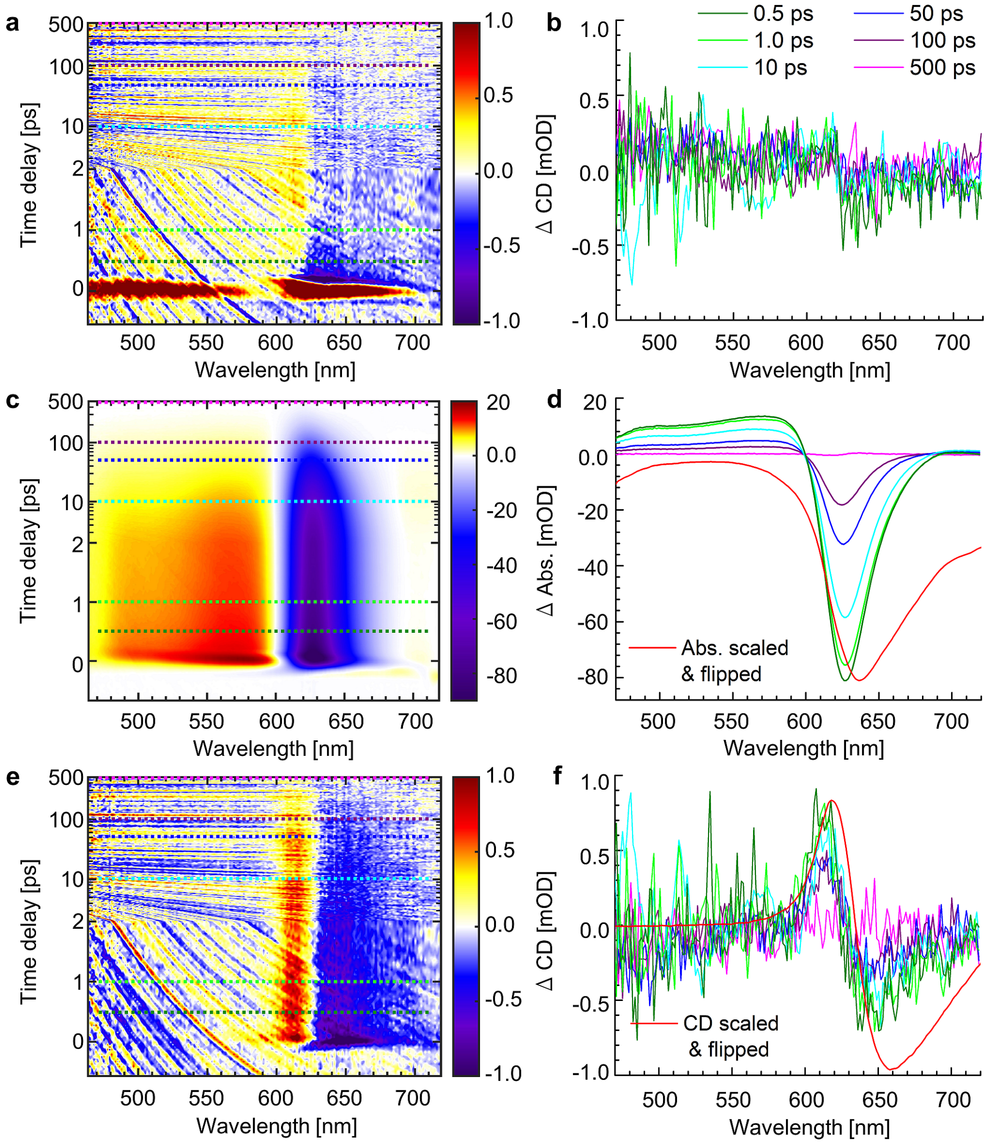

Figure S5: Time-resolved data of the achiral squaraine polymer p(SQ-R^0) in acetone and the subtracted data. (a) TRCD data and (c) TA data of p(SQ-R^0). Dotted horizontal lines (green to purple) symbolize the temporal position of the spectra shown in (b) of the TRCD map and in (d) of the TA map. (e) TRCD map resulting from subtracting the TRCD data of p(SQ-R^0) from the TRCD data of p(SQ-R^{2*}) and (f) the extracted spectra from temporal positions marked by the dotted lines. The time-resolved spectra are plotted together with the corresponding flipped and scaled steady-state absorption and CD spectra in red.

During a fine alignment process we used the achiral squaraine polymer p(SQ-R^0) in acetone with the same concentration and flow cuvette as described in the main text for the chiral squaraine polymer. By slightly adjusting the horizontal and vertical tilt of the focusing mirror of the pump beam we could decrease the TRCD signal to a minimum as shown in Figs. S5a and b. Even though there is some remaining signal in the TRCD map, this was much smaller than the signal of the chiral squaraine polymer p(SQ-R^{2*}) in Figs. 6a and b in the main text. We assume that this remaining signal had its origin in the ellipticity of the probe beams' polarizations and the beam profiles of pump and probe beams. The first issue will be discussed in more detail in Section S5. The second issue was explained in Section S1. The TA data (Figs. S5c, d) of the achiral squaraine polymer look similar to those

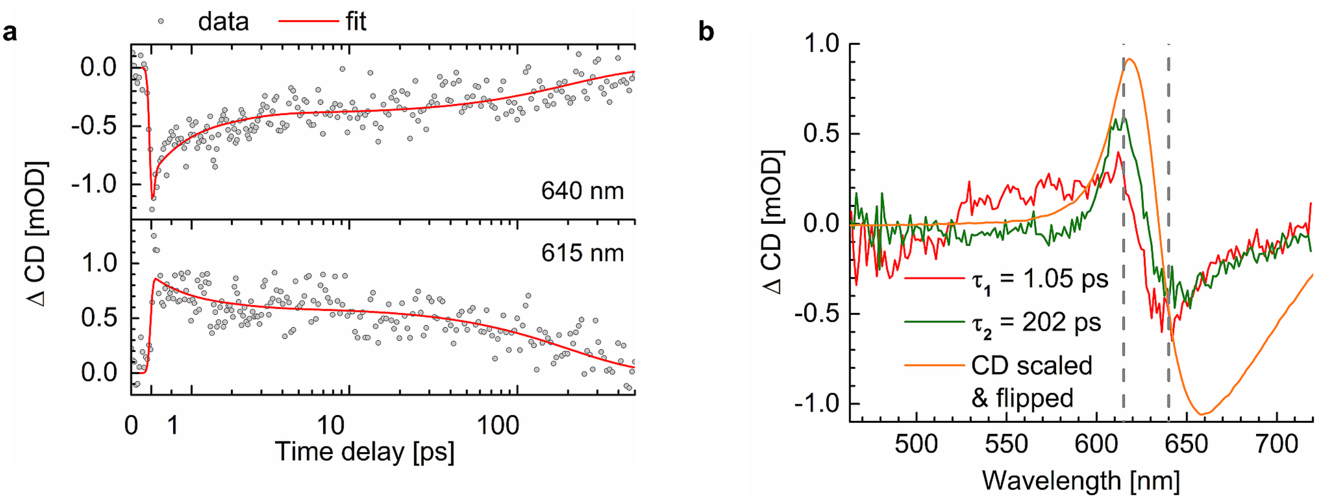

Figure S6: Results from global analysis of the subtracted TRCD dataset shown in Fig. S5e and f. (a) Data (grey dots) and fit (red lines) of time traces at 640 nm (top) and at 615 nm (bottom). (b) Corresponding decay-associated spectra (DAS) for two population times τ_1 (red) and τ_2 (green) and the sign-flipped and scaled steady-state CD spectrum of p(SQ-R_2*) plotted in yellow. The dashed gray lines indicate the wavelength positions of the data in (a).

of the chiral one (main text, Figs. 6c and d). To prove that the TRCD signal of p(SQ-R^{2*}) is genuine and the TRCD data of p(SQ-R^0) are just small remaining artifacts, one can simply subtract the datasets from each other. This leads to Figs. S5e and f. By comparing these data with the data of p(SQ-R^{2*}), we find that the dynamics are the same and the hypsochromic shift compared to the steady-state CD signal (red line) remains. We compare the global fit of the subtracted data in Fig. S6 with the plots in the main text (Fig. 7) and find that after subtraction, the dynamics are similar (Table 1, main text).

To quantify the signal-to-noise (S/N) ratio, we used the method described in 2. We extracted the minimum and maximum TRCD signal ($S^{640\text{ nm}}_{\text{TRCD}}, S^{615\text{ nm}}_{\text{TRCD}}$) at 640 nm and 615 nm, respectively, of the TRCD data of p(SQ-R^{2}*) in acetone in Fig. 6 (main text) according to eqn (77) at a delay time of 0.5 ps. Additionally we calculated the standard deviation (σ_t) of the delay times $t < 0$ ps (from -0.57 ps to -0.13 ps, which includes 12 time steps with a step size of 40 fs for each of the two mentioned wavelengths. See Sections 2.2 and 2.3 of the main text for details about time resolution and data acquisition, respectively.) to get the noise of the chirp-corrected data (see Section S1, Fig. S3 for more information about chirp correction of the data). With this the S/N ratios for the absolute magnitudes of the minimum and maximum TRCD signals are calculated as

$$|\frac{S}{N}|_{640\text{ nm}} = \frac{S^{\text{TRCD}}_{640\text{ nm}}}{\sigma_{t<0\text{ ps}}}, \tag{S1}$$

$$|\frac{S}{N}|_{615\text{ nm}} = \frac{S^{\text{TRCD}}_{615\text{ nm}}}{\sigma_{t<0\text{ ps}}}, \tag{S2}$$

respectively. The calculated S/N ratios, the signal at a delay time of 0.5 ps as well as the standard deviations σ_t of the time traces at 615 nm and 640 nm are summarized in Table S1.

Table S1: Signal to noise (S/N) ratios of the TRCD data of p(SQ-R^{2}*) in acetone at 615 nm and 640 nm. The noise was calculated as the standard deviation of the time traces for $t < 0$ps.

λ [nm]	$\sigma_{t<0\text{ ps}}$ [mOD]	$\lvert S^{\text{TRCD}}_{0.5\text{ ps}}\rvert$ [mOD]	$\lvert\frac{S}{N}\rvert$
615	0.0781	0.9195	11.770
640	0.0760	0.8945	11.766

The standard deviation of the noise for $t < 0$ ps of the two selected time traces is 0.077 mOD on average and at

0.5 ps the S/N ratio is determined to be 11.77 on average. Comparing the S/N ratio of our experiment with other TRCD experiments performed at similar conditions (i.e., using a broadband white-light spectrum in the visible, fs time resolution, and the differential absorptive approach) is rather difficult since the noise level or S/N ratio is often not quoted in publications.

S4 Spectra in Different Solvents

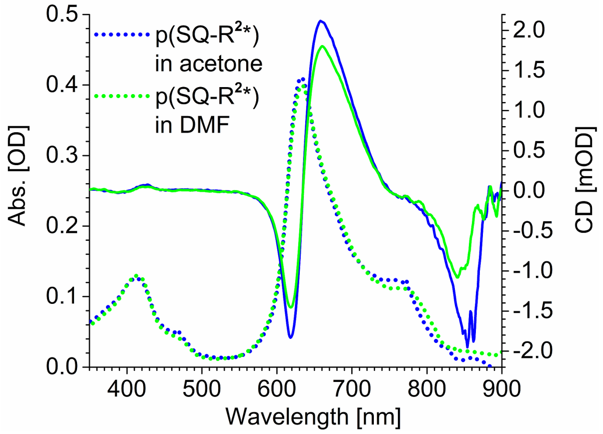

Figure S7: Absorption (dotted lines) and CD (solid lines) spectra of chiral p(SQ-R^{2*}) in acetone (blue) and DMF (green). The cuvette thickness was 0.5 mm and the concentration in DMF was $9.33 \cdot 10^{-5}$ mol/l and in acetone $9.88 \cdot 10^{-5}$ mol/l.

We discuss the TA data in the main text by comparing them with a similar squaraine polymer from literature which was measured in DMF.[3] Therefore we plot the absorption and CD spectra of the squaraine polymer p(SQ-R_2*) in DMF and in acetone in Fig. S7 for comparison. The shape of the absorption bands is the same. In DMF the CD signal is weaker than in acetone. This difference could be caused by a somewhat larger fraction of zigzag random coil components in DMF than in acetone.

S5 Imperfect Circular Polarization in TRCD

In this section, we investigate how robust is our TRCD measurement against imperfection in the circular probe light polarization. Pump–probe spectroscopy results from four-wave mixing, with the signal field

$$E_i^{\mathrm{S}} = \chi_{ijkl} E_j^{\mathrm{Pr}} E_k^{\mathrm{P}} E_l^{\mathrm{P}*} = \chi_{ijkl} E_j^{\mathrm{Pr}} I^{\mathrm{P}}, \tag{S3}$$

where χ_{ijkl} is the third-order susceptibility tensor and E^{Pr}, E^{P} are the probe and pump fields, respectively. To describe electric fields, we will use the positive-frequency part of the electric field, also called complex analytical signal. The intensity can then be defined simply as $I = E \cdot E$ (apart from some prefactors). In our geometry, we choose linear pump polarization,

$$\mathbf{E}^{\mathrm{P}} = E^{\mathrm{P}} \mathbf{e}_x, \tag{S4}$$

and probe with elliptic polarization,

$$\mathbf{E}^{\mathrm{Pr}} = E_x^{\mathrm{Pr}} \mathbf{e}_x + E_y^{\mathrm{Pr}} \mathbf{e}_y = E^{\mathrm{Pr}} \left(\cos(\alpha) \mathbf{e}_x + \sin(\alpha) e^{i\delta} \mathbf{e}_y \right), \tag{S5}$$

In case of perfect circular polarization we do not need the term $e^{i\delta}$. Note that pump and probe beams are roughly collinear along the z direction in our experiment such that we will not discuss anisotropy effects. Indeed, the goal of this section is to distinguish the linear (in polarization) and chiral transient signals. In our geometry, assuming an isotropic sample, the nonlinear signal field can be expressed as

$$\mathbf{E}^{\mathrm{S}} = I^{\mathrm{P}} E^{\mathrm{Pr}} \left\{ \chi_{xxxx} \cos(\alpha) + \chi_{xyxx} \sin(\alpha) e^{i\delta} \right\} \mathbf{e}_x + \left\{ \chi_{yyxx} \sin(\alpha) e^{i\delta} + \chi_{yxxx} \cos(\alpha) \right\} \mathbf{e}_y. \tag{S6}$$

The elements χ_{xxxx} and χ_{yyxx} of the susceptibility tensor form the standard TA signal, whereas χ_{xyxx} and χ_{yxxx} are the chiral contributions to the overall susceptibility. For symmetry reasons $\chi_{xyxx} =$ - χ_{yxxx} holds. χ_{xxxx} is parallel oriented in the sense of response to the pump with the same polarization and χ_{yyxx} is oriented with the polarization of the response perpendicular to the pump polarization.

The TA signal is self-heterodyned, i.e., the signal co-propagates with the probe pulse and interferes with it in the spectrometer. We thus have for the detected intensity

$$I^{\mathrm{det}} \propto |\mathbf{E}^{S} + \mathbf{E}^{\mathrm{Pr}}|^2 = |\mathbf{E}^{\mathrm{S}}|^2 + \mathbf{E}^{\mathrm{Pr}} \cdot \mathbf{E}^{\mathrm{S}*} + \mathbf{E}^{\mathrm{Pr}*} \cdot \mathbf{E}^{\mathrm{S}} + |\mathbf{E}^{\mathrm{Pr}}|^2 = I^{\mathrm{S}} + I^{\mathrm{Pr}} + 2\Re \left\{ \mathbf{E}^{\mathrm{Pr}*} \cdot \mathbf{E}^{\mathrm{S}} \right\}, \tag{S7}$$

because the nonlinear signal field E^{S} is much weaker than the probe field, I^{S} is very small and can be neglected and $\Re$ denotes the real part. The TA is calculated as

$$\begin{aligned}
\Delta \mathrm{OD} &= \log_{10}\left(\frac{I_0}{I}\right) = \lg\left(\frac{I_0}{I}\right) = \lg\left(\frac{I^{\mathrm{Pr}}}{I^{\mathrm{det}}}\right) = -\lg\left(\frac{I^{\mathrm{det}}}{I^{\mathrm{Pr}}}\right) \\
&= -\lg\left(\frac{I^{\mathrm{Pr}} + 2\Re\left\{\mathbf{E}^{\mathrm{Pr}*} \cdot \mathbf{E}^{\mathrm{S}}\right\}}{I^{\mathrm{Pr}}}\right) = -\lg\left(\frac{I^{\mathrm{Pr}}}{I^{\mathrm{Pr}}} + \frac{2\Re\left\{\mathbf{E}^{\mathrm{Pr}*} \cdot \mathbf{E}^{\mathrm{S}}\right\}}{I^{\mathrm{Pr}}}\right) \\
&= -\lg\left(1 + \frac{2\Re\left\{\mathbf{E}^{\mathrm{Pr}*} \cdot \mathbf{E}^{\mathrm{S}}\right\}}{I^{\mathrm{Pr}}}\right) \approx -\frac{2\Re\left\{\mathbf{E}^{\mathrm{Pr}*} \cdot \mathbf{E}^{\mathrm{S}}\right\}}{I^{\mathrm{Pr}}},
\end{aligned} \tag{S8}$$

because $-\lg(1 + x) \approx x$ for small values of x. Again, this is justified by the probe beam intensity being much higher than that of the nonlinear signal. This is easily checked in the experiment, taking into account the the values of Δ CD, which are typically on the order of 10^{-3} (see Fig. S6 for example).

In order to incorporate the polarization sensitivity of the detection, we consider independent intensity and phase modulation of the x and y components of both the probe and signal fields that propagate together after the sample to the detector, so that they experience the same phase and intensity modulation. Because absolute scaling of the

fields doesn't matter because relative absorbance change is measured, we consider the y component multiplied by a complex number β instead of having both x and y modulated. Inserting the signal field from eqn (S6), we get

$$\begin{aligned}
\Delta\mathrm{OD} &= \frac{2\Re\left\{\mathbf{E}^{\mathrm{Pr}*}\cdot\mathbf{E}^{\mathrm{S}}\right\}}{I^{\mathrm{Pr}}} \\
&= \frac{2\Re\left\{\mathbf{E}^{\mathrm{Pr}*}I^{\mathrm{P}}\mathbf{E}^{\mathrm{Pr}}\left\{\chi_{xxxx}\cos(\alpha)+\chi_{xyxx}\sin(\alpha)e^{i\delta}\right\}\mathbf{e}_x+\left\{\chi_{yyxx}\sin(\alpha)e^{i\delta}\beta+\chi_{yxxx}\cos(\alpha)\beta\right\}\mathbf{e}_y\right\}}{I^{\mathrm{Pr}}} \\
&= I^{\mathrm{P}}2\Re\Big\{\left\{\chi_{xxxx}\cos^2(\alpha)+\chi_{xyxx}\sin(\alpha)\cos(\alpha)e^{i\delta}\right\} \\
&+\left\{\chi_{yyxx}\sin^2(\alpha)+\chi_{yxxx}\cos(\alpha)\sin(\alpha)e^{-i\delta}\right\}|\beta|^2\Big\} \\
&= I^{\mathrm{P}}2\Re\left\{\chi_{xxxx}\cos^2(\alpha)+\chi_{yyxx}\sin^2(\alpha)|\beta|^2\right\} \\
&+I^{\mathrm{P}}2\Re\left\{\chi_{xyxx}\sin(\alpha)\cos(\alpha)e^{i\delta}+\chi_{yxxx}\sin(\alpha)\cos(\alpha)e^{-i\delta}|\beta|^2\right\} \\
&= I^{\mathrm{P}}2\Re\left\{\chi_{xxxx}\cos^2(\alpha)+\chi_{yyxx}\sin^2(\alpha)|\beta|^2\right\} \\
&+I^{\mathrm{P}}\sin(\alpha)\cos(\alpha)2\Re\left\{\chi_{xyxx}e^{i\delta}+\chi_{yxxx}e^{-i\delta}|\beta|^2\right\}.
\end{aligned} \tag{S9}$$

Using $\sin(\alpha)\cos(\alpha)=\frac{1}{2}\sin(2\alpha)$, we obtain

$$\begin{aligned}
\Delta\mathrm{OD} &= I^{\mathrm{P}}2\Re\left\{\chi_{xxxx}\cos^2(\alpha)+\chi_{yyxx}\sin^2(\alpha)|\beta|^2\right\} \\
&+I^{\mathrm{P}}\sin(2\alpha)\Re\left\{\chi_{xyxx}e^{i\delta}+\chi_{yxxx}e^{-i\delta}|\beta|^2\right\}.
\end{aligned} \tag{S10}$$

The first line of eqn (S10) is the standard TA signal, with an isotropic signal acquired under the magic angle when $\cos^2(\alpha)=2\sin^2(\alpha)$. The polarization-sensitive detection alters the combination of the two components of the response, and thus introduces anisotropy. The second line of eqn (S10) is the chiral signal, as can be seen from the presence of the respective susceptibility elements. Let us now consider CD, i.e., set $\alpha=\frac{\pi}{4}$, and additionally for LCP $\delta=\frac{\pi}{2}$ and for RCP $\delta=-\frac{\pi}{2}$ using the relations $\cos^2(\frac{\pi}{4})=\frac{1}{2}=\sin^2(\frac{\pi}{4})$ and $\sin(2\frac{\pi}{4})=1$:

$$\begin{aligned}
&\Delta\mathrm{OD}_{\mathrm{LCP}}-\Delta\mathrm{OD}_{\mathrm{RCP}} \\
&=\left(I^{\mathrm{P}}2\Re\left\{\chi_{xxxx}\cos^2(\alpha)+\chi_{yyxx}\sin^2(\alpha)|\beta|^2\right\}+I^{\mathrm{P}}\sin(2\alpha)\Re\left\{\chi_{xyxx}e^{i\delta_{\mathrm{LCP}}}+\chi_{yxxx}e^{-i\delta_{\mathrm{LCP}}}|\beta|^2\right\}\right) \\
&-\left(I^{\mathrm{P}}2\Re\left\{\chi_{xxxx}\cos^2(\alpha)+\chi_{yyxx}\sin^2(\alpha)|\beta|^2\right\}+I^{\mathrm{P}}\sin(2\alpha)\Re\left\{\chi_{xyxx}e^{i\delta_{\mathrm{RCP}}}+\chi_{yxxx}e^{-i\delta_{\mathrm{RCP}}}|\beta|^2\right\}\right) \qquad \text{(S11)} \\
&=\left(I^{\mathrm{P}}2\Re\left\{\chi_{xxxx}\frac{1}{2}+\chi_{yyxx}\frac{1}{2}|\beta|^2\right\}+I^{\mathrm{P}}\Re\left\{\chi_{xyxx}e^{i\frac{\pi}{2}}+\chi_{yxxx}e^{-i\frac{\pi}{2}}|\beta|^2\right\}\right) \\
&-\left(I^{\mathrm{P}}2\Re\left\{\chi_{xxxx}\frac{1}{2}+\chi_{yyxx}\frac{1}{2}|\beta|^2\right\}+I^{\mathrm{P}}\Re\left\{\chi_{xyxx}e^{i\frac{-\pi}{2}}+\chi_{yxxx}e^{-i\frac{-\pi}{2}}|\beta|^2\right\}\right) \qquad \text{(S12)}
\end{aligned}$$

The standard TA signal (the terms with χ_{xxxx} and χ_{yyxx}) cancels out because it does not depend on δ. We thus obtain

$$\begin{aligned}
&\Delta\mathrm{OD}_{\mathrm{LCP}}-\Delta\mathrm{OD}_{\mathrm{RCP}} \\
&=I^{\mathrm{P}}\Re\left\{\chi_{xyxx}e^{i\frac{\pi}{2}}+\chi_{yxxx}e^{-i\frac{\pi}{2}}|\beta|^2\right\}-I^{\mathrm{P}}\Re\left\{\chi_{xyxx}e^{-i\frac{\pi}{2}}+\chi_{yxxx}e^{i\frac{\pi}{2}}|\beta|^2\right\} \\
&=I^{\mathrm{P}}\left\{\Re\left\{\chi_{xyxx}e^{i\frac{\pi}{2}}-\chi_{xyxx}e^{-i\frac{\pi}{2}}\right\}+\Re\left\{\chi_{yxxx}e^{-i\frac{\pi}{2}}-\chi_{yxxx}e^{i\frac{\pi}{2}}\right\}|\beta|^2\right\} \\
&=I^{\mathrm{P}}\frac{1}{2}\left\{-2\Im\left\{\chi_{xyxx}\right\}+2\Im\left\{\chi_{yxxx}\right\}|\beta|^2\right\} \\
&=-I^{\mathrm{P}}2\Im\left\{\chi_{xyxx}-\chi_{yxxx}|\beta|^2\right\}.
\end{aligned} \tag{S13}$$

Finally, using $\chi_{xyxx}=-\chi_{yxxx}$, we have

$$\Delta\mathrm{OD}_{\mathrm{LCP}}-\Delta\mathrm{OD}_{\mathrm{RCP}}=I^{\mathrm{P}}2\Im\left\{\chi_{yxxx}\left(1+|\beta|^2\right)\right\}. \tag{S14}$$

For perfectly circular polarization of the probe, the polarization sensitivity of detection does not mix the chiral and non-chiral transient signals. Like in the case of standard TA spectroscopy, polarization-sensitive detection can introduce some anisotropy into TRCD, but by itself, without other imperfections, it does not introduce a linear pump–probe signal.

Let us now analyze what happens if we have imperfect probe polarization. In that case, we have a deviation both in α, $\alpha_{\mathrm{RCP/LCP}} = \frac{\pi}{4} + \delta\alpha_{\mathrm{R/L}}$, and in the phase, $\delta_{\mathrm{RCP/LCP}} = \pm\frac{\pi}{2} + \delta\delta_{\mathrm{R/L}}$, where the deltas indicate small deviation from the respective parameters. For slightly elliptical polarization such as from our setup the deviations will be small. Because of the symmetry, one can also expect them to be of similar magnitude. That is, only the lowest perturbative terms in $\delta\alpha$ and $\delta\delta$ contribute to all expressions. Including the polarization imperfection, eqn (S10) reads

$$\begin{aligned}
&\Delta\mathrm{OD}_{\mathrm{LCP}} - \Delta\mathrm{OD}_{\mathrm{RCP}} && \text{(S15)}\\
&= I^{\mathrm{P}} 2\Re\left\{\chi_{xxxx}\cos^2(\alpha_{\mathrm{LCP}}) + \chi_{yyxx}\sin^2(\alpha_{\mathrm{LCP}})|\beta|^2\right\}\\
&+ I^{\mathrm{P}}\sin(2(\frac{\pi}{4} + \delta\alpha_{\mathrm{LCP}}))\Re\left\{\chi_{xyyx}e^{i(\frac{\pi}{2}+\delta\delta_{\mathrm{LCP}})} + \chi_{yxxx}e^{-i(\frac{\pi}{2}+\delta\delta_{\mathrm{LCP}})}|\beta|^2\right\}\\
&- I^{\mathrm{P}} 2\Re\left\{\chi_{xxxx}\cos^2(\alpha_{\mathrm{RCP}}) + \chi_{yyxx}\sin^2(\alpha_{\mathrm{RCP}})|\beta|^2\right\}\\
&- I^{\mathrm{P}}\sin(2(\frac{\pi}{4} + \delta\alpha_{\mathrm{RCP}}))\Re\left\{\chi_{xyyx}e^{i(-\frac{\pi}{2}+\delta\delta_{\mathrm{RCP}})} + \chi_{yxxx}e^{-i(-\frac{\pi}{2}+\delta\delta_{\mathrm{RCP}})}|\beta|^2\right\} && \text{(S16)}\\
&= I^{\mathrm{P}} 2\Re\left\{\left(\cos^2(\alpha_{\mathrm{LCP}}) - \cos^2(\alpha_{\mathrm{RCP}})\right)\chi_{xxxx} + \left(\sin^2(\alpha_{\mathrm{LCP}}) - \sin^2(\alpha_{\mathrm{RCP}})\right)|\beta|^2\chi_{yyxx}\right\}\\
&+ I^{\mathrm{P}}\sin(2(\frac{\pi}{4} + \delta\alpha_{\mathrm{LCP}}))\Re\left\{\chi_{xyyx}e^{i(\frac{\pi}{2}+\delta\delta_{\mathrm{LCP}})} + \chi_{yxxx}e^{-i(\frac{\pi}{2}+\delta\delta_{\mathrm{LCP}})}|\beta|^2\right\}\\
&- I^{\mathrm{P}}\sin(2(\frac{\pi}{4} + \delta\alpha_{\mathrm{RCP}}))\Re\left\{\chi_{xyyx}e^{i(-\frac{\pi}{2}+\delta\delta_{\mathrm{RCP}})} + \chi_{yxxx}e^{-i(-\frac{\pi}{2}+\delta\delta_{\mathrm{RCP}})}|\beta|^2\right\}. && \text{(S17)}
\end{aligned}$$

Using $\sin(2(x + \frac{\pi}{4})) = \cos(2x)$ we obtain

$$\begin{aligned}
&\Delta\mathrm{OD}_{\mathrm{LCP}} - \Delta\mathrm{OD}_{\mathrm{RCP}} && \text{(S18)}\\
&= I^{\mathrm{P}} 2\Re\left\{\left(\cos^2(\alpha_{\mathrm{LCP}}) - \cos^2(\alpha_{\mathrm{RCP}})\right)\chi_{xxxx} + \left(\sin^2(\alpha_{\mathrm{LCP}}) - \sin^2(\alpha_{\mathrm{RCP}})\right)|\beta|^2\chi_{yyxx}\right\}\\
&- I^{\mathrm{P}}\cos(2\delta\alpha_{\mathrm{L}})\Im\left\{\chi_{xyyx}e^{i\delta\delta_{\mathrm{L}}} - \chi_{yxxx}e^{-i\delta\delta_{\mathrm{L}}}|\beta|^2\right\}\\
&- I^{\mathrm{P}}\cos(2\delta\alpha_{\mathrm{R}})\Im\left\{\chi_{xyyx}e^{i\delta\delta_{\mathrm{R}}} - \chi_{yxxx}e^{-i\delta\delta_{\mathrm{R}}}|\beta|^2\right\}. && \text{(S19)}
\end{aligned}$$

From the first line in eqn (S19) we see that $\delta\alpha$ introduces the standard pump–probe signal into the expected chiral one, although with a weak amplitude. Interestingly, the linear (in polarization) pump–probe signal still vanishes as long as the RCP and LCP polarizations are perturbed in the same way, i.e., $\delta\alpha_{\mathrm{L}} = \pm\delta\alpha_{\mathrm{R}}$. Importantly, when the detection is not very polarization sensitive ($|\beta|^2 \to 1$), the chiral signal is (taking $\chi_{xyyx} = -\chi_{yxxx}$)

$$(\Delta\mathrm{OD}_{\mathrm{LCP}} - \Delta\mathrm{OD}_{\mathrm{RCP}})_{\mathrm{chiral}} = 2I^{\mathrm{P}}\left(\cos(2\delta\alpha_{\mathrm{L}})\cos(\delta\delta_{\mathrm{L}}) + \cos(2\delta\alpha_{\mathrm{R}})\cos(\delta\delta_{\mathrm{R}})\right)\Im\left\{\chi_{yxxx}\right\}, \qquad \text{(S20)}$$

i.e., it corresponds to the correct chiral signal (compare to eqn (S19)) multiplied by some number smaller than 1, for polarization very close to 1. Imperfect circular (elliptical) polarization in general introduces linear pump–probe signal and makes the chiral signal weaker. When the two polarizations are imperfect (elliptical) in the same way, meaning they have the same polarization angle and phase deviation from perfect circular, the linear pump–probe signal is not introduced.

In order to obtain a standard TA signal, one can sum the two circularly probed signals together, i.e.,

$$\Delta\mathrm{OD}_{\mathrm{LCP}} + \Delta\mathrm{OD}_{\mathrm{RCP}} \tag{S21}$$

$$\begin{aligned} &= I^{\mathrm{P}} 2\Re\left\{\left(\cos^2\alpha_{\mathrm{LCP}} + \cos^2\alpha_{\mathrm{RCP}}\right)\chi_{xxxx} + \left(\sin^2\alpha_{\mathrm{LCP}} + \sin^2\alpha_{\mathrm{RCP}}\right)|\beta|^2\chi_{yyxx}\right\} \\ &- I^{\mathrm{P}}\cos\left(2\delta\alpha_{\mathrm{L}}\right)\Im\left\{\chi_{xyxx}e^{i\delta\delta_{\mathrm{L}}} - \chi_{yxxx}e^{-i\delta\delta_{\mathrm{L}}}|\beta|^2\right\} \\ &+ I^{\mathrm{P}}\cos\left(2\delta\alpha_{\mathrm{R}}\right)\Im\left\{\chi_{xyxx}e^{i\delta\delta_{\mathrm{R}}} - \chi_{yxxx}e^{-i\delta\delta_{\mathrm{R}}}|\beta|^2\right\}. \end{aligned} \tag{S22}$$

In this combination of signals, the chiral signal is suppressed in a way that is robust against polarization imperfections, for example for $|\beta|^2 \to 1$ one gets

$$\left(\Delta\mathrm{OD}_{\mathrm{LCP}} + \Delta\mathrm{OD}_{\mathrm{RCP}}\right)_{\mathrm{chiral}} = 2I^{\mathrm{P}}\left(\cos\left(2\delta\alpha_{\mathrm{L}}\right)\cos\left(\delta\delta_{\mathrm{L}}\right) - \cos\left(2\delta\alpha_{\mathrm{R}}\right)\cos\left(\delta\delta_{\mathrm{R}}\right)\right)\Im\left\{\chi_{yxxx}\right\}. \tag{S23}$$

For slightly perturbed polarization, i.e., for small $\delta\alpha$ and $\delta\delta$, this expression nearly vanishes (the leading term is quadratic in the δ terms). Furthermore, for polarization perturbed in the same way ($\delta\alpha_{\mathrm{L}} = \pm\delta\alpha_{\mathrm{R}}, \delta\delta_{\mathrm{L}} = \pm\delta\delta_{\mathrm{R}}$), the chiral contribution vanishes completely. Summarizing, with imperfect polarization, the combination $\Delta\mathrm{OD}_{\mathrm{LCP}} - \Delta\mathrm{OD}_{\mathrm{RCP}}$ in general contains a relatively strong chiral signal and a weak linear signal. The combination $\Delta\mathrm{OD}_{\mathrm{LCP}} + \Delta\mathrm{OD}_{\mathrm{RCP}}$ contains a strong linear pump–probe signal and a very weak chiral signal.

S6 Orientational Averages Relevant for Magic Angle TRCD and TA

The transformation of a vector $\boldsymbol{A}$ between lab ($\boldsymbol{A} = (A_X, A_Y, A_Z)$) and molecule ($\boldsymbol{A} = (A_x, A_y, A_z)$) frame is given as[4]

$$\boldsymbol{A}_{\text{lab}} = \boldsymbol{\Phi}\boldsymbol{A}_{\text{mol}} \tag{S24}$$

with the matrix $\boldsymbol{\Phi}$ reading

$$\begin{aligned}\boldsymbol{\Phi} &= \begin{pmatrix} \cos(\phi)\cos(\theta)\cos(\chi) - \sin(\phi)\sin(\chi) & \sin(\phi)\cos(\theta)\cos(\chi) + \cos(\phi)\sin(\chi) & -\sin(\theta)\cos(\chi) \\ -\cos(\phi)\cos(\theta)\sin(\chi) - \sin(\phi)\cos(\chi) & -\sin(\phi)\cos(\theta)\sin(\chi) + \cos(\phi)\cos(\chi) & \sin(\theta)\sin(\chi) \\ \cos(\phi)\sin(\theta) & \sin(\phi)\sin(\theta) & \cos(\theta) \end{pmatrix} \\ &= \begin{pmatrix} \Phi_{Xx} & \Phi_{Yx} & \Phi_{Zx} \\ \Phi_{Xy} & \Phi_{Yy} & \Phi_{Zy} \\ \Phi_{Xz} & \Phi_{Yz} & \Phi_{Zz} \end{pmatrix}\end{aligned} \tag{S25}$$

and the isotropic orientational average of a quantity f is obtained as

$$\langle f \rangle_{\text{orient}} = \frac{1}{8\pi^2} \int_0^{\pi} \int_0^{2\pi} \int_0^{2\pi} f(\theta, \phi, \chi)\ \sin(\theta)\ d\theta d\phi d\chi \tag{S26}$$

S6.1 TRCD, Proof of Eqn (47) of the Main Text

The orientational average relevant for TRCD (first line of eqn (47) of the main text) reads

$$\left\langle f_{mn} |\vec{\mu}_K \cdot \vec{e}_{\text{pu}}|^2 \right\rangle_{\text{orient}} = A + B \tag{S27}$$

with

$$A = \left\langle \left(\vec{n}_{\text{pr}} \cdot \vec{R}_{mn}\right) \left(\vec{n}_{\text{pr}} \cdot \vec{S}_{mn}\right) |\vec{\mu}_K \cdot \vec{e}_{\text{pu}}|^2 \right\rangle_{\text{orient}}, \tag{S28}$$

where we introduced the vector

$$\vec{S}_{mn} = \vec{\mu}_m \times \vec{\mu}_n \tag{S29}$$

as the cross product of electric transition dipole moments of chromophores m and n, and

$$B = \left\langle \vec{n}_{\text{pr}} \cdot \left((\hat{Q}_n \vec{n}_{\text{pr}}) \times \vec{\mu}_m \right) |\vec{\mu}_K \cdot \vec{e}_{\text{pu}}|^2 \right\rangle_{\text{orient}}. \tag{S30}$$

containing the intrinsic electric quadrupole transition tensor $\hat{Q}_n$ of chromophore m.[5] In order to calculate the average with respect to random orientation of the molecular system, we transfer all molecular quantities from the laboratory frame, in which the vectros of the external fields are fixed to the molecular frame by eqn (S24). The orientational average can then be executed with respect to the transformation matrices ϕ. In setup (a) (Figure 1 of the main text) we have

$$\vec{e}_{\text{pu}} = \vec{e}_z \cos\alpha - \vec{e}_y \sin\alpha\,, \tag{S31}$$

$$\vec{n}_{\text{pr}} = \vec{e}_z\,, \text{and} \tag{S32}$$

$$\vec{n}_{\text{pu}} = \vec{e}_z \sin\alpha + \vec{e}_y \cos\alpha\,. \tag{S33}$$

Using eqns (S28), (S31) and (S32), the orientational average contained in quantity A (eqn (S28)) becomes

$$\begin{aligned} A &= \left\langle \left(\vec{e}_z \cdot \vec{R}_{mn}\right) \left(\vec{e}_z \cdot \vec{S}_{mn}\right) |\vec{\mu}_K \cdot (\vec{e}_z \cos\alpha - \vec{e}_y \sin\alpha)|^2 \right\rangle_{\text{orient}} \\ &= \textstyle\sum_{i,j,k,l} R_{mn}^{(i)} S_{mn}^{(j)} \mu_K^{(k)} \mu_K^{(l)} \left\langle \phi_{Zi}\phi_{Zj}(\phi_{Zk}\cos\alpha - \phi_{Yk}\sin\alpha)(\phi_{Zl}\cos\alpha - \phi_{Yl}\sin\alpha) \right\rangle_{\text{orient}}, \end{aligned} \tag{S34}$$

where i, j, k, l count the x, y and z components of the different vectors in the molecule-fixed frame. By noting that $\langle\phi_{Zi}\phi_{Zj}\phi_{Zk}\phi_{Yl}\rangle_{\text{orient}} = 0$ and $\langle\phi_{Zi}\phi_{Zj}\phi_{Zk}\phi_{Yl}\rangle_{\text{orient}} = 0$, we obtain

$$A = \sum_{i,j,k,l} R_{mn}^{(i)} S_{mn}^{(j)} \mu_K^{(k)} \mu_K^{(l)} \left(\langle\phi_{Zi}\phi_{Zj}\phi_{Zk}\phi_{Zl}\rangle_{\text{orient}} \cos^2\alpha + \langle\phi_{Zi}\phi_{Zj}\phi_{Yk}\phi_{Yl}\rangle_{\text{orient}} \sin^2\alpha\right). \tag{S35}$$

Using the following properties[4]

$$\langle\phi_{Fi}\phi_{Fj}\phi_{Fk}\phi_{Fl}\rangle_{\text{orient}} = \frac{\delta_{ij}\delta_{kl} + \delta_{ik}\delta_{jl} + \delta_{il}\delta_{jk}}{15} \tag{S36}$$

and

$$\langle\phi_{Fi}\phi_{Fj}\phi_{Gk}\phi_{Gl}\rangle_{\text{orient}} = \frac{4\delta_{ij}\delta_{kl} - \delta_{ik}\delta_{jl} - \delta_{il}\delta_{jk}}{15} \tag{S37}$$

for $F \neq G$, we obtain

$$\begin{aligned} A &= \tfrac{1}{15}\cos^2\alpha\left(\vec{R}_{mn}\cdot\vec{S}_{mn}|\vec{\mu}_K|^2 + 2(\vec{R}_{mn}\cdot\vec{\mu}_K)(\vec{S}_{mn}\cdot\vec{\mu}_K)\right) \\ &\quad + \tfrac{1}{15}\sin^2\alpha\left(2\vec{R}_{mn}\cdot\vec{S}_{mn}|\vec{\mu}_K|^2 - (\vec{R}_{mn}\cdot\vec{\mu}_K)(\vec{S}_{mn}\cdot\vec{\mu}_K)\right) \\ &= \tfrac{1}{15}\vec{R}_{mn}\cdot\vec{S}_{mn}|\vec{\mu}_K|^2(2-\cos^2\alpha) + \tfrac{1}{15}(\vec{R}_{mn}\cdot\vec{\mu}_K)(\vec{S}_{mn}\cdot\vec{\mu}_K)(3\cos^2\alpha - 1). \end{aligned} \tag{S38}$$

The average in quantity B (eqn (S30)) is expressed as

$$\begin{aligned} B &= \left\langle\left(\mu_m^{(Y)}(\hat{Q}_n\cdot\vec{n}_{\text{pr}})^{(X)} - \mu_m^{(X)}(\hat{Q}_n\cdot\vec{n}_{\text{pr}})^{(Y)}\right)|\vec{\mu}_K\cdot\vec{e}_{\text{pu}}|^2\right\rangle_{\text{orient}} \\ &= \left\langle\left(\mu_m^{(Y)}Q_n^{(ZX)} - \mu_m^{(X)}Q_n^{(ZY)}\right)|\vec{\mu}_K\cdot(\vec{e}_z\cos\alpha - \vec{e}_y\sin\alpha)|^2\right\rangle_{\text{orient}} \\ &= \left\langle\left(\mu_m^{(Y)}Q_n^{(ZX)} - \mu_m^{(X)}Q_n^{(ZY)}\right)(\mu_K^{(Z)}\cos\alpha - \mu_K^{(Y)}\sin\alpha)^2\right\rangle_{\text{orient}}. \end{aligned} \tag{S39}$$

Transformation to the molecular frame gives

$$\begin{aligned} B &= \sum_{i_1,i_2,i_3,i_4,i_5} \mu_m^{(i_1)} Q_n^{(i_2 i_3)} \mu_K^{(i_4)} \mu_K^{(i_5)} \langle(\phi_{Yi_1}\phi_{Zi_2}\phi_{Xi_3} - \phi_{Xi_1}\phi_{Zi_2}\phi_{Yi_3}) \\ &\times (\phi_{Zi_4}\cos\alpha - \phi_{Yi_4}\sin\alpha)(\phi_{Zi_5}\cos\alpha - \phi_{Yi_5}\sin\alpha)\rangle_{\text{orient}} \\ &= \sum_{i_1,i_2,i_3,i_4,i_5} \mu_m^{(i_1)} Q_n^{(i_2 i_3)} \mu_K^{(i_4)} \mu_K^{(i_5)} \langle(\phi_{Yi_1}\phi_{Zi_2}\phi_{Xi_3} - \phi_{Xi_1}\phi_{Zi_2}\phi_{Yi_3}) \\ &\times (\cos^2(\alpha)\phi_{Zi_4}\phi_{Zi_5} + \sin^2(\alpha)\phi_{Yi_4}\phi_{Yi_5} - 2\cos(\alpha)\sin(\alpha)\phi_{Zi_4}\phi_{Yi_5})\rangle_{\text{orient}}. \end{aligned} \tag{S40}$$

The orientational average of $\phi_{\lambda_1 i_1}\phi_{\lambda_2 i_2}\phi_{\lambda_3 i_3}\phi_{\lambda_4 i_4}\phi_{\lambda_5 i_5}$ where the $\lambda_i = \{X, Y, Z\}$ refer to the vector components in the laboratory frame is obtained as[6]

$$\langle\phi_{\lambda_1 i_1}\phi_{\lambda_2 i_2}\phi_{\lambda_3 i_3}\phi_{\lambda_4 i_4}\phi_{\lambda_5 i_5}\rangle_{\text{orient}} = \frac{1}{30}\vec{E}^{\text{T}}\hat{I}\vec{L} \tag{S41}$$

with the vectors

$$\vec{E}^{\text{T}} = (\epsilon_{i_1 i_2 i_3}\delta_{i_4 i_5}, \epsilon_{i_1 i_2 i_4}\delta_{i_3 i_5}, \epsilon_{i_1 i_2 i_5}\delta_{i_3 i_4}, \epsilon_{i_1 i_3 i_4}\delta_{i_2 i_5}, \epsilon_{i_1 i_3 i_5}\delta_{i_2 i_4}, \epsilon_{i_1 i_4 i_5}\delta_{i_2 i_3}) \tag{S42}$$

and the transpose of the vector $\vec{L}$

$$\vec{L}^{\text{T}} = (\epsilon_{\lambda_1\lambda_2\lambda_3}\delta_{\lambda_4\lambda_5}, \epsilon_{\lambda_1\lambda_2\lambda_4}\delta_{\lambda_3\lambda_5}, \epsilon_{\lambda_1\lambda_2\lambda_5}\delta_{\lambda_3\lambda_4}, \epsilon_{\lambda_1\lambda_3\lambda_4}\delta_{\lambda_2\lambda_5}, \epsilon_{\lambda_1\lambda_3\lambda_5}\delta_{\lambda_2\lambda_4}, \epsilon_{\lambda_1\lambda_4\lambda_5}\delta_{\lambda_2\lambda_3}), \tag{S43}$$

where ϵ_{ijk} and δ_{ij} are the Levi-Civita and the Kronecker-Delta symbols, and the matrix $\hat{I}$ reads

$$\hat{I} = \begin{pmatrix} 3 & -1 & -1 & 1 & 1 & 0 \\ -1 & 3 & -1 & -1 & 0 & 1 \\ -1 & -1 & 3 & 0 & -1 & -1 \\ 1 & 0 & -1 & -1 & 3 & -1 \\ 0 & 1 & -1 & 1 & -1 & 3 \end{pmatrix} \tag{S44}$$

With the above relations (eqns (S41)-(S44)) we obtain

$$\begin{aligned}
\langle \phi_{Yi_1}\phi_{Zi_2}\phi_{Xi_3}\phi_{Zi_4}\phi_{Zi_5}\rangle_{\text{orient}} &= \frac{1}{30}\left(\epsilon_{i_1i_2i_3}\delta_{i_4i_5} - \epsilon_{i_1i_3i_4}\delta_{i_2i_5} - \epsilon_{i_1i_3i_5}\delta_{i_2i_4}\right) && \text{(S45)}\\
\langle \phi_{Xi_1}\phi_{Zi_2}\phi_{Yi_3}\phi_{Zi_4}\phi_{Zi_5}\rangle_{\text{orient}} &= \frac{1}{30}\left(-\epsilon_{i_1i_2i_3}\delta_{i_4i_5} + \epsilon_{i_1i_3i_4}\delta_{i_2i_5} + \epsilon_{i_1i_3i_5}\delta_{i_2i_4}\right) && \text{(S46)}\\
\langle \phi_{Yi_1}\phi_{Zi_2}\phi_{Xi_3}\phi_{Yi_4}\phi_{Yi_5}\rangle_{\text{orient}} &= \frac{1}{30}\left(3\epsilon_{i_1i_2i_3}\delta_{i_4i_5} - \epsilon_{i_1i_2i_4}\delta_{i_3i_5} - \epsilon_{i_1i_2i_5}\delta_{i_3i_4}\right. \\
&+ \left.\epsilon_{i_1i_3i_4}\delta_{i_2i_5} + \epsilon_{i_1i_3i_5}\delta_{i_2i_4}\right) && \text{(S47)}\\
\langle \phi_{Xi_1}\phi_{Zi_2}\phi_{Yi_3}\phi_{Yi_4}\phi_{Yi_5}\rangle_{\text{orient}} &= \frac{1}{30}\left(-\epsilon_{i_1i_2i_3}\delta_{i_4i_5} - \epsilon_{i_1i_2i_4}\delta_{i_3i_5} - \epsilon_{i_1i_2i_5}\delta_{i_3i_4}\right) && \text{(S48)}\\
\langle \phi_{Yi_1}\phi_{Zi_2}\phi_{Xi_3}\phi_{Zi_4}\phi_{Yi_5}\rangle_{\text{orient}} &= 0 && \text{(S49)}\\
\langle \phi_{Xi_1}\phi_{Zi_2}\phi_{Yi_3}\phi_{Zi_4}\phi_{Yi_5}\rangle_{\text{orient}} &= 0 && \text{(S50)}
\end{aligned}$$

resulting in the orientational average

$$\begin{aligned}
B &= \frac{1}{30}\sum_{i_1,i_2,i_3,i_4,i_5} \mu_m^{(i_1)} Q_n^{(i_2i_3)} \mu_K^{(i_4)} \mu_K^{(i_5)} [2\left(\epsilon_{i_1i_2i_3}\delta_{i_4i_5} - \epsilon_{i_1i_3i_4}\delta_{i_2i_5} - \epsilon_{i_1i_3i_5}\delta_{i_2i_4}\right)\cos^2\alpha \\
&+ \left(4\epsilon_{i_1i_2i_3}\delta_{i_4i_5} + \epsilon_{i_1i_3i_4}\delta_{i_2i_5} + \epsilon_{i_1i_3i_5}\delta_{i_2i_4}\right)\sin^2\alpha]\,. && \text{(S51)}
\end{aligned}$$

By noting that the quadrupole tensor is symmetric ($Q_n^{(i_2i_3)} = Q_n^{(i_3i_2)}$) and $\epsilon_{i_1i_2i_3} = -\epsilon_{i_1i_3i_2}$, the sums over the first and the fourth term are seen to vanish and we get

$$\begin{aligned}
B &= -\frac{1}{30}\sum_{i_1,i_2,i_3,i_4,i_5} \mu_m^{(i_1)} Q_n^{(i_2i_3)} \mu_K^{(i_4)} \mu_K^{(i_5)} [\left(\epsilon_{i_1i_3i_4}\delta_{i_2i_5} + \epsilon_{i_1i_3i_5}\delta_{i_2i_4}\right)\left(3\cos^2\alpha - 1\right) \\
&= -\frac{1}{15}\sum_{i_1,i_2,i_3,i_4} \mu_m^{(i_1)} Q_n^{(i_2i_3)} \mu_K^{(i_4)} \mu_K^{(i_2)} \epsilon_{i_1i_3i_4}(3\cos^2\alpha - 1) \\
&= -\frac{1}{15}\vec{\mu}_m \cdot \left((\hat{Q}_n\vec{\mu}_K) \times \vec{\mu}_K\right)\left(3\cos^2\alpha - 1\right). && \text{(S52)}
\end{aligned}$$

In setup (b) (Figure 1 of the main text) we have

$$\begin{aligned}
\vec{e}_{\text{pu}} &= \vec{e}_z\cos\alpha + \vec{e}_x\sin\alpha\,, && \text{(S53)}\\
\vec{n}_{\text{pr}} &= \vec{e}_z\,, \text{and} && \text{(S54)}\\
\vec{n}_{\text{pu}} &= \vec{e}_y && \text{(S55)}\\
& && \text{(S56)}
\end{aligned}$$

and A (eqn (S28)) is obtained as

$$\begin{aligned}
A &= \left\langle \left(\vec{e}_z \cdot \vec{R}_{mn}\right)\left(\vec{e}_z \cdot \vec{S}_{mn}\right)\left|\vec{\mu}_K \cdot (\vec{e}_z\cos\alpha + \vec{e}_x\sin\alpha)\right|^2 \right\rangle_{\text{orient}} \\
&= \textstyle\sum_{i,j,k,l} R_{mn}^{(i)} S_{mn}^{(j)} \mu_{K0}^{(k)} \mu_{K0}^{(l)} \left\langle \phi_{Zi}\phi_{Zj}(\phi_{Zk}\cos\alpha + \phi_{Xk}\sin\alpha)(\phi_{Zl}\cos\alpha + \phi_{Xl}\sin\alpha)\right\rangle_{\text{orient}} \\
&= \textstyle\sum_{i,j,k,l} R_{mn}^{(i)} S_{mn}^{(j)} \mu_{K0}^{(k)} \mu_{K0}^{(l)} \left(\langle \phi_{Zi}\phi_{Zj}\phi_{Zk}\phi_{Zl}\rangle_{\text{orient}}\cos^2\alpha + \langle \phi_{Zi}\phi_{Zj}\phi_{Xk}\phi_{Xl}\rangle_{\text{orient}}\sin^2\alpha\right)\right), && \text{(S57)}
\end{aligned}$$

which resembles the expression of setup (a) in eqn (S35), since it holds that

$$\langle \phi_{Zi}\phi_{Zj}\phi_{Yk}\phi_{Yl}\rangle_{\text{orient}} = \langle \phi_{Zi}\phi_{Zj}\phi_{Xk}\phi_{Xl}\rangle_{\text{orient}} \,. \tag{S58}$$

Hence, the remaining part of the proof is identical to that given for setup (a) above.

In case of quantity B (eqn (S30)) we obtain for setup (b)

$$\begin{aligned} B &= \left\langle \left(\mu_m^{(Y)}Q_n^{(ZX)} - \mu_m^{(X)}Q_n^{(ZY)}\right)|\vec{\mu}_K \cdot (\vec{e}_z\cos\alpha + \vec{e}_x\sin\alpha)|^2\right\rangle_{\text{orient}} \\ &= \left\langle \left(\mu_m^{(Y)}Q_n^{(ZX)} - \mu_m^{(X)}Q_n^{(ZY)}\right)(\mu_K^{(Z)}\cos\alpha + \mu_K^{(X)}\sin\alpha)^2\right\rangle_{\text{orient}} \\ &= \sum_{i_1,i_2,i_3,i_4,i_5} \mu_m^{(i_1)}Q_n^{(i_2i_3)}\mu_K^{(i_4)}\mu_K^{(i_5)}\,\langle(\phi_{Yi_1}\phi_{Zi_2}\phi_{Xi_3} - \phi_{Xi_1}\phi_{Zi_2}\phi_{Yi_3}) \\ &\times \quad (\cos^2(\alpha)\phi_{Zi_4}\phi_{Zi_5} + \sin^2(\alpha)\phi_{Xi_4}\phi_{Xi_5} + 2\cos(\alpha)\sin(\alpha)\phi_{Zi_4}\phi_{Xi_5})\rangle_{\text{orient}} \end{aligned} \tag{S59}$$

From eqns (S41)-(S44), is follows that

$$\langle \phi_{Yi_1}\phi_{Zi_2}\phi_{Xi_3}\phi_{Xi_4}\phi_{Xi_5}\rangle_{\text{orient}} = -\langle \phi_{Xi_1}\phi_{Zi_2}\phi_{Yi_3}\phi_{Yi_4}\phi_{Yi_5}\rangle_{\text{orient}} \tag{S60}$$

$$\langle \phi_{Xi_1}\phi_{Zi_2}\phi_{Yi_3}\phi_{Xi_4}\phi_{Xi_5}\rangle_{\text{orient}} = -\langle \phi_{Yi_1}\phi_{Zi_2}\phi_{Yi_3}\phi_{Yi_4}\phi_{Xi_5}\rangle_{\text{orient}} \tag{S61}$$

$$\langle \phi_{Xi_1}\phi_{Zi_2}\phi_{Yi_3}\phi_{Zi_4}\phi_{Xi_5}\rangle_{\text{orient}} = 0 \tag{S62}$$

and, hence, the same expression for B is obtained (eqn (S52)), as for setup (a).

S6.2 TA, Proof of Eqn (50) of the Main Text

For the present setups (a) and (b) (Figure 1 of the main text) the probe pulse propagates in z-direction and its left(l)- and right(r) circular polarization are described by the polarization vectors

$$\vec{e}_{\text{pr}}(\text{l/r}) = \frac{1}{\sqrt{2}}(\vec{e}_x \pm i\vec{e}_y)\,. \tag{S63}$$

With these vectors, assuming setup (a) (eqn (S31)), the orientational average relevant for TA (eqn (50)) reads

$$\begin{aligned} &\left\langle |\vec{\mu}_A \cdot \vec{e}_{\text{pr}}^{(\text{l/r})}|^2 |\vec{\mu}_N \cdot \vec{e}_{\text{pu}}|^2\right\rangle_{\text{orient}} \\ &= \tfrac{1}{2}\langle |\vec{\mu}_A \cdot (\vec{e}_x \pm i\vec{e}_y)|^2|\vec{\mu}_N \cdot (\vec{e}_z\cos\alpha - \vec{e}_y\sin\alpha)|^2\rangle_{\text{orient}} \\ &= \tfrac{1}{2}\left\langle \left((\vec{\mu}_A\cdot\vec{e}_x)^2 + (\vec{\mu}_A\cdot\vec{e}_y)^2\right)(\vec{\mu}_N\cdot\vec{e}_z\cos\alpha - \vec{\mu}_N\cdot\vec{e}_y\sin\alpha)^2\right\rangle_{\text{orient}} \,. \end{aligned} \tag{S64}$$

Transforming the molecular vectors from the laboratory- into the molecule-fixed frame (eqn (S24)) we obtain

$$\begin{aligned} &\left\langle |\vec{\mu}_A \cdot \vec{e}_{\text{pr}}^{(\text{l/r})}|^2 |\vec{\mu}_N \cdot \vec{e}_{\text{pu}}|^2\right\rangle_{\text{orient}} \\ &= \tfrac{1}{2}\textstyle\sum_{i,j,k,l}\left\langle (\mu_A^{(i)}\phi_{Xi}\mu_A^{(j)}\phi_{Xj} + \mu_A^{(i)}\phi_{Yi}\mu_A^{(j)}\phi_{Yj}) + \mu_N^{(k)}(\phi_{Zk}\cos\alpha - \phi_{Yk}\sin\alpha)\right. \\ &\qquad \left.\times\mu_N^{(l)}(\phi_{Zl}\cos\alpha - \phi_{Yl}\sin\alpha)\right\rangle_{\text{orient}} \\ &= \tfrac{1}{2}\textstyle\sum_{i,j,k,l}\mu_A^{(i)}\mu_A^{(j)}\mu_N^{(k)}\mu_N^{(l)}\,\langle(\phi_{Xi}\phi_{Xj} + \phi_{Yi}\phi_{Yj})(\phi_{Zk}\phi_{Zl}\cos^2\alpha + \phi_{Yk}\phi_{Yl}\sin^2\alpha)\rangle_{\text{orient}} \\ &= \tfrac{1}{2}\textstyle\sum_{i,j,k,l}\mu_A^{(i)}\mu_A^{(j)}\mu_N^{(k)}\mu_N^{(l)} \\ &\times\left((1+\cos^2\alpha)\langle\phi_{Xi}\phi_{Xj}\phi_{Zk}\phi_{Zl}\rangle_{\text{orient}} + (1-\cos^2\alpha)\langle\phi_{Yi}\phi_{Yj}\phi_{Yk}\phi_{Yl}\rangle_{\text{orient}}\right)\,, \end{aligned} \tag{S65}$$

where we have used

$$\langle \phi_{Fi}\phi_{Fj}\phi_{Gk}\phi_{Gl}\rangle_{\text{orient}} = \langle \phi_{Fi}\phi_{Fj}\phi_{Hk}\phi_{Hl}\rangle_{\text{orient}} \,. \tag{S66}$$

With eqns (S36) and (S37) the orientational average becomes

$$\begin{aligned}
&\left\langle |\vec{\mu}_A \cdot \vec{e}_{\mathrm{pr}}^{(\mathrm{l/r})}|^2 |\vec{\mu}_N \cdot \vec{e}_{\mathrm{pu}}|^2 \right\rangle_{\mathrm{orient}} \\
&= \tfrac{1+\cos^2\alpha}{60} \left(4|\vec{\mu}_A|^2|\vec{\mu}_N|^2 - 2(\vec{\mu}_A \cdot \vec{\mu}_N)^2\right) + \tfrac{1-\cos^2\alpha}{30} \left(|\vec{\mu}_A|^2|\vec{\mu}_N|^2 + 2(\vec{\mu}_A \cdot \vec{\mu}_N)^2\right) \\
&= \tfrac{3+\cos^2\alpha}{30} |\vec{\mu}_A|^2|\vec{\mu}_N|^2 + \tfrac{1-3\cos^2\alpha}{30} (\vec{\mu}_A \cdot \vec{\mu}_N)^2 \,, \qquad \text{(S67)}
\end{aligned}$$

which is identical with eqn (50) of the main text. In case of setup (b) (Fig. 1 of main text), we have eqn (S53) for the pump-pulse polarization and the orientational average is given as

$$\begin{aligned}
&\left\langle |\vec{\mu}_A \cdot \vec{e}_{\mathrm{pr}}^{(\mathrm{l/r})}|^2 |\vec{\mu}_N \cdot \vec{e}_{\mathrm{pu}}|^2 \right\rangle_{\mathrm{orient}} \\
&= \tfrac{1}{2} \left\langle |\vec{\mu}_A \cdot (\vec{e}_x \pm i\vec{e}_y)|^2 |\vec{\mu}_N \cdot (\vec{e}_z \cos\alpha + \vec{e}_x \sin\alpha)|^2 \right\rangle_{\mathrm{orient}} \\
&= \tfrac{1}{2} \left\langle \left((\vec{\mu}_A \cdot \vec{e}_x)^2 + (\vec{\mu}_A \cdot \vec{e}_y)^2\right) \left(\vec{\mu}_N \cdot \vec{e}_z \cos\alpha + \vec{\mu}_N \cdot \vec{e}_x \sin\alpha\right)^2 \right\rangle_{\mathrm{orient}} \,. \qquad \text{(S68)}
\end{aligned}$$

After transformation to molecule fixed vectors we get

$$\begin{aligned}
&\left\langle |\vec{\mu}_A \cdot \vec{e}_{\mathrm{pr}}^{(\mathrm{l/r})}|^2 |\vec{\mu}_N \cdot \vec{e}_{\mathrm{pu}}|^2 \right\rangle_{\mathrm{orient}} \\
&= \tfrac{1}{2} \sum_{i,j,k,l} \Big\langle (\mu_A^{(i)} \phi_{Xi} \mu_A^{(j)} \phi_{Xj} + \mu_A^{(i)} \phi_{Yi} \mu_A^{(j)} \phi_{Yj}) + \mu_N^{(k)} (\phi_{Zk} \cos\alpha + \phi_{Xk} \sin\alpha) \\
&\qquad \times \mu_N^{(l)} (\phi_{Zl} \cos\alpha + \phi_{Xl} \sin\alpha) \Big\rangle_{\mathrm{orient}} \\
&= \tfrac{1}{2} \sum_{i,j,k,l} \mu_A^{(i)} \mu_A^{(j)} \mu_N^{(k)} \mu_N^{(l)} \left\langle (\phi_{Xi}\phi_{Xj} + \phi_{Yi}\phi_{Yj})(\phi_{Zk}\phi_{Zl} \cos^2\alpha + \phi_{Xk}\phi_{Xl} \sin^2\alpha) \right\rangle_{\mathrm{orient}} \\
&= \tfrac{1}{2} \sum_{i,j,k,l} \mu_A^{(i)} \mu_A^{(j)} \mu_N^{(k)} \mu_N^{(l)} \\
&\quad \times \left((1+\cos^2\alpha) \left\langle \phi_{Xi}\phi_{Xj}\phi_{Zk}\phi_{Zl} \right\rangle_{\mathrm{orient}} + (1-\cos^2\alpha) \left\langle \phi_{Xi}\phi_{Xj}\phi_{Xk}\phi_{Xl} \right\rangle_{\mathrm{orient}}\right) \,, \qquad \text{(S69)}
\end{aligned}$$

which is identical to eqn (S65) obtained for setup (a), since it holds that $\langle \phi_{Xi}\phi_{Xj}\phi_{Xk}\phi_{Xl} \rangle_{\mathrm{orient}} = \langle \phi_{Yi}\phi_{Yj}\phi_{Yk}\phi_{Yl} \rangle_{\mathrm{orient}}$. Hence, the remaining part of the proof is identical to that for setup (a) given above.

S6.3 Experimentally Determined TA Data with Different Probe Polarization Directions

Additional TA experiments were performed to check that no anisotropy effects arise when measuring with magic angle configuration. We measured TA spectra of p(SQ-R^{2}*) in acetone without the TRCD setup. The white-light beam was guided around the TRCD setup directly behind the half-wave plate (see Fig. 2, main text) with two mirrors and focused into the cuvette with the same achromatic lens as mentioned in Section 2.1. The polarization state of the linearly polarized white-light pulses was set to 54.7° in comparison to the linear polarization state of the pump pulses. Due to the fact that the beam did not pass through several optics from the other experiment, especially one achromatic lens (that focused the beam into the polarization grating), the dispersion of the white-light pulses was decreased. Additionally, the beam diameter of the white light focused into the cuvette was larger because it did not pass the TRCD setup including focusing and collimating optics that would decrease the beam diameter. Therefore, the overlap of pump and probe beam in the cuvette was different than in the TRCD experiment. However, despite these differences between the two setups, the resulting TA data (see Fig. S8) are very similar. Only around t = 0 ps the signal of the coherent artifact is stronger in the case without the TRCD setup, which can be explained by the differences discussed above. All other parameters of the measurements were held constant (see Section 2, main text). In summary we proof theoretically and experimentally that the TA data as well as the TRCD data, measured with the same setup, are anisotropy-free.

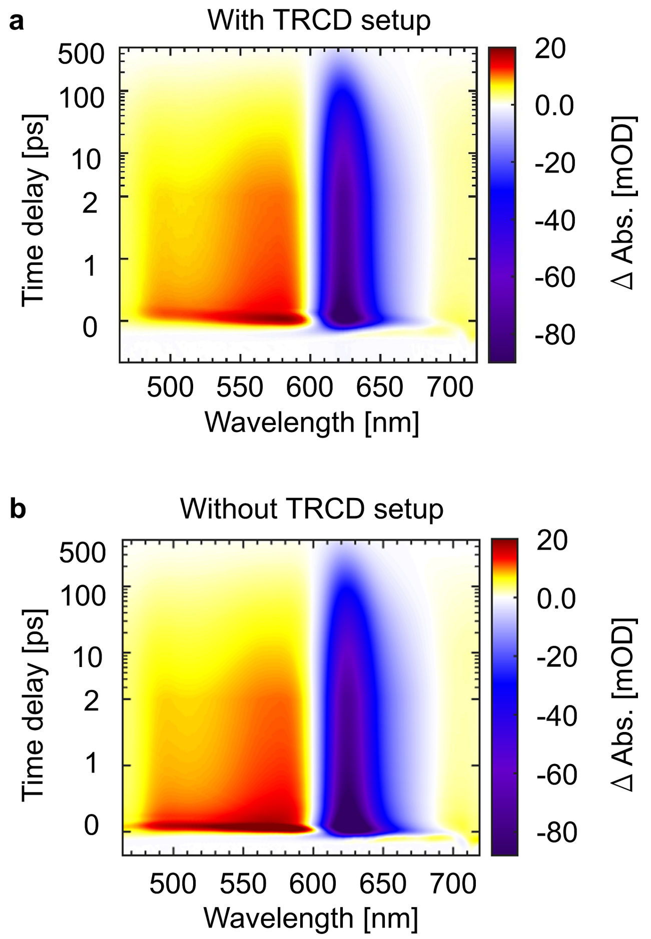

Figure S8: TA data recorded with (a) and without (b) the new TRCD setup. The top figure includes the same data shown in the main text in Fig. 6c. The bottom TA map includes the data taken without the TRCD setup shown in Fig. 2 (main text) and an angle of 54.7° was set between the linearly polarized pump and probe beams.

S7 Derivation of Eqn (27)

The matrix element squared, $|h^{(\mathrm{pr,CD})}_{M,2N}|^2$, for ESA in TRCD is given as a difference of a matrix element squared for interaction of the excitonic system with left (l) and right (r) circularly polarized light,

$$|h^{(\mathrm{pr,CD})}_{M,2N}|^2 = |h^{(\mathrm{pr,l})}_{M,2N}|^2 - |h^{(\mathrm{pr,r})}_{M,2N}|^2. \tag{S70}$$

We start by transforming the matrix element $h^{(\mathrm{pr},l/r)}_{M,2N}$ from the delocalized into the localized basis,

$$h^{(\mathrm{pr,l/r})}_{M,2N} = \sum_{k<l,m} c^{(M)}_m c^{(2N)}_{kl} h_{kl,m}, \tag{S71}$$

where in the local basis, it holds that

$$h_{kl,m} = \delta_{k,m} h_{l0} + \delta_{l,m} h_{k0}, \tag{S72}$$

Expanding the local matrix elements in powers of the wavevector of the external field,

$$h_{m0} \approx h^{(0)}_{m0} + h^{(1)}_{m0}, \tag{S73}$$

gives the zeroth-order contribution [5]

$$h^{(0)}_{m0} = i\frac{E_0}{2}(\vec{e}^{\,(\mathrm{l/r})}_{\mathrm{pr}} \cdot \vec{\mu}_m) \tag{S74}$$

with the polarization unit vector $\vec{e}^{\,(\mathrm{l/r})}_{\mathrm{pr}}$ of the left (l) or right (r) circularly polarized probe field with electric field amplitude E_0, and the electric transition dipole moment $\vec{\mu}_m$ of chromophore m. The first-order contribution $h^{(1)}_{m0}$ contains magnetic transition dipole and electric transition quadrupole contributions [5] that can be combined by introducing center-of-mass and relative coordinates of the electrons of the chromophores,

$$h^{(1)}_{m0} \approx -\frac{E_0}{2}(\vec{k}_{\mathrm{pr}} \cdot \vec{R}_m)(\vec{e}^{\,(l/r)}_{\mathrm{pr}} \cdot \vec{\mu}_m) + \frac{1}{2}\vec{k}_{\mathrm{pr}} \cdot (\hat{Q}_m \vec{e}^{\,(l/r)}_{\mathrm{pr}})\,. \tag{S75}$$

Here $\vec{R}_m$ points to the center of the mth chromophore, $\vec{k}_{\mathrm{pr}}$ is the wavevector of the probe field, and $\hat{Q}_m$ is the intrinsic electric transition moment of chromophore m defined with respect to its center. [5] Please note that we have neglected the intrinsic magnetic transition dipole moment of the chromophores, since the resulting intrinsic rotational strength can assumed to be small for the present squaraine chromophore (as judged from the conservative circular dichroism spectrum).

Sorting the square of the matrix elements on the r.h.s. of eqn (S70) with respect to powers in the probe field wavevector $\vec{k}_{\mathrm{pr}}$ results in

$$|h^{(\mathrm{pr,l/r})}_{M,2N}|^2 \approx |h^{(\mathrm{pr,l/r})}_{M,2N}|^2(0) + |h^{(\mathrm{pr,l/r})}_{M,2N}|^2(1) \tag{S76}$$

with the zeroth-order contribution

$$\begin{aligned} |h^{(\mathrm{pr,l/r})}_{M,2N}|^2(0) &= \sum_{k>l,m>n} c^{(2N)}_{kl} c^{(2N)}_{mn} \left(c^{(M)}_k h^{(0)}_{l0} + c^{(M)}_l h^{(0)}_{k0}\right)\left(c^{(M)}_m h^{(0)*}_{n0} + c^{(M)}_n h^{(0)*}_{m0}\right) \\ &= \frac{E_0^2}{4} \sum_{k>l,m>n} c^{(2N)}_{kl} c^{(2N)}_{mn} \left(c^{(M)}_k (\vec{e}^{\,(\mathrm{l/r})}_{\mathrm{pr}} \cdot \vec{\mu}_{l0}) + c^{(M)}_l (\vec{e}^{\,(\mathrm{l/r})}_{\mathrm{pr}} \cdot \vec{\mu}_{k0})\right) \\ &\times \left(c^{(M)}_m (\vec{e}^{\,(\mathrm{r/l})}_{\mathrm{pr}} \cdot \vec{\mu}_n) + c^{(M)}_n (\vec{e}^{\,(\mathrm{l/r})}_{\mathrm{pr}} \cdot \vec{\mu}_m)\right), \end{aligned} \tag{S77}$$

where the property $\vec{e}^{\,(\mathrm{l/r})*}_{\mathrm{pr}} = \vec{e}^{\,(\mathrm{r/l})}_{\mathrm{pr}}$ of the polarization vectors was used.

The first-order contribution reads

$$
\begin{aligned}
|h_{M,2N}^{(\mathrm{pr,l/r})}|^2(1) &= \sum_{k>l} c_{kl}^{(2N)} \left(c_k^{(M)} h_{l0}^{(0)} + c_l^{(M)} h_{k0}^{(0)} \right) \sum_{m>n} c_{mn}^{(2N)} \left(c_m^{(M)} h_{n0}^{(1)*} + c_n^{(M)} h_{m0}^{(1)*} \right) \\
&+ \sum_{k>l} c_{kl}^{(2N)} \left(c_k^{(M)} h_{l0}^{(1)} + c_l^{(M)} h_{k0}^{(1)} \right) \sum_{m>n} c_{mn}^{(2N)} \left(c_m^{(M)} h_{n0}^{(0)*} + c_n^{(M)} h_{m0}^{(0)*} \right) \\
&= \sum_{k>l,m>n} c_{kl}^{(2N)} c_{mn}^{(2N)} \Big(c_k^{(M)} c_m^{(M)} (v_{ln} + v_{nl}^*) + c_k^{(M)} c_n^{(M)} (v_{lm} + v_{ml}^*) \\
&+ c_l^{(M)} c_m^{(M)} (v_{kn} + v_{nk}^*) + c_l^{(M)} c_n^{(M)} (v_{km} + v_{mk}^*) \Big) , \qquad \text{(S78)}
\end{aligned}
$$

where in the last two lines we introduced the function

$$
v_{mn} = h_{m0}^{(0)} h_{n0}^{(1)*} = -i \frac{E_0^2}{4} (\vec{e}_{\mathrm{pr}} \cdot \vec{\mu}_m) \left\{ (\vec{k}_{\mathrm{pr}} \cdot \vec{R}_n)(\vec{e}_{\mathrm{pr}}^{\,*} \cdot \vec{\mu}_n) + \frac{1}{2} \vec{k}_{\mathrm{pr}} \cdot (\hat{Q}_n \vec{e}_{\mathrm{pr}}^{\,*}) \right\} . \qquad \text{(S79)}
$$

In order to simplify the above expression, we formally remove the restrictions $k > l$ and $m > n$ in the double sums and request that it holds that $c_{mn}^{(2N)} = c_{nm}^{(2N)}$ and similarly $c_{kl}^{(2N)} = c_{lk}^{(2N)}$. To correct for the double counting of locally excited two-exciton states $|mn\rangle = |nm\rangle$ and $|kl\rangle = |lk\rangle$, a factor 1/4 is introduced and the above $|h_{M,2N}^{(\mathrm{pr,CD})}|^2$ becomes, after interchanging summation indices,

$$
\begin{aligned}
|h_{M,2N}^{(\mathrm{pr,l/r})}|^2(1) &= \frac{1}{2} \sum_{k,l,m,n} c_{kl}^{(2N)} c_{mn}^{(2N)} \Big(c_k^{(M)} c_m^{(M)} \Re(v_{ln}) + c_k^{(M)} c_n^{(M)} \Re(v_{lm}) \\
&+ c_l^{(M)} c_m^{(M)} \Re(v_{kn}) + c_l^{(M)} c_n^{(M)} \Re(v_{km}) \Big) , \qquad \text{(S80)}
\end{aligned}
$$

where $\Re$ denotes the real part.

The matrix element $|h_{M,2N}^{(\mathrm{pr,CD})}|^2$ of ESA then follows as a difference between the above matrix elements for left and right circularly polarized probe field polarization vectors $\vec{e}_{\mathrm{pr}}^{\,(\mathrm{l})}$ and $\vec{e}_{\mathrm{pr}}^{\,(\mathrm{r})}$, respectively. The zeroth-order contribution $|h_{M,2N}^{(\mathrm{pr,l})}|^2(0) - |h_{M,2N}^{(\mathrm{pr,r})}|^2(0)$ is seen to vanish by interchanging summation variables $(m \leftrightarrow k)$ and $(n \leftrightarrow l)$ in eqn (S77). The first non-vanishing contribution is obtained from the first-order terms

$$
\begin{aligned}
|h_{M,2N}^{(\mathrm{pr,CD})}|^2 &= |h_{M,2N}^{(\mathrm{pr,l})}|^2(1) - |h_{M,2N}^{(\mathrm{pr,r})}|^2(1) \\
&= \frac{1}{2} \sum_{k,l,m,n} c_{kl}^{(2N)} c_{mn}^{(2N)} \Big(c_k^{(M)} c_m^{(M)} u_{ln} + c_k^{(M)} c_n^{(M)} u_{lm} \\
&+ c_l^{(M)} c_m^{(M)} u_{kn} + c_l^{(M)} c_n^{(M)} u_{km} \Big) , \qquad \text{(S81)}
\end{aligned}
$$

where

$$
\begin{aligned}
u_{mn} &= \Re(v_{mn}(\vec{e}_{\mathrm{pr}}^{\,(\mathrm{l})}) - v_{mn}(\vec{e}_{\mathrm{pr}}^{\,(\mathrm{r})})) \\
&= -i \frac{E_0^2}{4} (\vec{k}_{\mathrm{pr}} \cdot \vec{R}_n) \Big((\vec{e}_{\mathrm{pr}}^{\,(\mathrm{l})} \cdot \vec{\mu}_m)(\vec{e}_{\mathrm{pr}}^{\,(\mathrm{l})*} \cdot \vec{\mu}_n) - (\vec{e}_{\mathrm{pr}}^{\,(\mathrm{l})*} \cdot \vec{\mu}_m)(\vec{e}_{\mathrm{pr}}^{\,(\mathrm{l})} \cdot \vec{\mu}_n) \Big) \\
&= -i \frac{E_0^2}{4} \left\{ (\vec{k}_{\mathrm{pr}} \cdot \vec{R}_n)(\vec{e}_{\mathrm{pr}}^{\,(\mathrm{l})} \times \vec{e}_{\mathrm{pr}}^{\,(\mathrm{l})*}) \cdot (\vec{\mu}_m \times \vec{\mu}_n) + \frac{1}{2} (\vec{e}_{\mathrm{pr}}^{\,(\mathrm{l})} \times \vec{e}_{\mathrm{pr}}^{\,(\mathrm{l})*}) \cdot \Big(\vec{\mu}_m \times (\hat{Q}_n \vec{k}_{\mathrm{pr}}) \Big) \right\} \qquad \text{(S82)}
\end{aligned}
$$

and where the vector identity $(\vec{a} \cdot \vec{c})(\vec{b} \cdot \vec{d}) - (\vec{b} \cdot \vec{c})(\vec{a} \cdot \vec{d}) = (\vec{a} \times \vec{b}) \cdot (\vec{c} \times \vec{d})$ was used. Taking into account $\vec{e}_{\mathrm{pr}}^{\,(\mathrm{l})} \times \vec{e}_{\mathrm{pr}}^{\,(\mathrm{l})*} = -i \vec{n}_{\mathrm{pr}}$ we get

$$
u_{mn} = \frac{E_0^2}{4} \left\{ s_{mn} - \frac{1}{2} \vec{n}_{\mathrm{pr}} \cdot \Big(\vec{\mu}_m \times (\hat{Q}_n \vec{k}_{\mathrm{pr}}) \Big) \right\} \qquad \text{(S83)}
$$

with

$$s_{mn} = -(\vec{k}_{\mathrm{pr}} \cdot \vec{R}_n)(\vec{n}_{\mathrm{pr}} \cdot (\vec{\mu}_m \times \vec{\mu}_n)). \tag{S84}$$

Noting that it holds that

$$s_{mn} + s_{nm} = (\vec{k}_{\mathrm{pr}} \cdot \vec{R}_{mn})(\vec{n}_{\mathrm{pr}} \cdot (\vec{\mu}_m \times \vec{\mu}_n)), \tag{S85}$$

with the distance vector

$$\vec{R}_{mn} = \vec{R}_m - \vec{R}_n \,, \tag{S86}$$

we split the sums in eqn (S81) $\sum_{k,l,m,n} ... = \frac{1}{2}\left(\sum_{k,l,m,n} ... + \sum_{k,l,m,n} ...\right)$ and interchange summation indices in the second sums as needed to obtain

$$\begin{aligned} &\tfrac{1}{2}\textstyle\sum_{k,l,m,n} c_{kl}^{(2N)} c_{mn}^{(2N)} \left(c_k^{(M)} c_m^{(M)} s_{ln} + c_k^{(M)} c_n^{(M)} s_{lm} + c_l^{(M)} c_m^{(M)} s_{kn} + c_l^{(M)} c_n^{(M)} s_{km}\right) \\ &= \tfrac{1}{4}\textstyle\sum_{k,l,m,n} c_{kl}^{(2N)} c_{mn}^{(2N)} \left(c_k^{(M)} c_m^{(M)} h_{ln} + c_k^{(M)} c_n^{(M)} h_{lm} + c_l^{(M)} c_m^{(M)} h_{kn} + c_l^{(M)} c_n^{(M)} h_{km} \,,\right) \end{aligned} \tag{S87}$$

with

$$h_{mn} = s_{mn} + s_{nm} \,. \tag{S88}$$

Finally, we split the sums in eqn (S87) according to

$$\sum_{k,l,m,n} ... = \sum_{k>l,m>n} ... + \sum_{k>l,m<n} ... + \sum_{k<l,m>n} ... + \sum_{k<l,m<n} .... \tag{S89}$$

The expression in the sums is found to be invariant with respect to interchanges in summation indices $m \leftrightarrow n$ and $k \leftrightarrow l$. Therefore, the above sum can be expressed as $4\sum_{k>l,m>n} ...$ and, after including the quadrupole contributions (second term in eqn (S83)) we obtain the final matrix element in eqns (26) and (27) of the main text.

S8 Derivation of Eqn (36)

We define a symmetric kinetic matrix $\hat{\tilde{A}}$ by

$$\tilde{A}_{MN} = A_{MN}\sqrt{\frac{P_N^{(\mathrm{eq})}}{P_M^{(\mathrm{eq})}}}\,, \tag{S90}$$

where A_{MN} are the matrix elements of the original kinetic matrix (eqn (30)) and $P_K^{(\mathrm{eq})}$ is the (quasi)equilibrium population of one-exciton state $|K\rangle$ (eqn (35)). The original eigenvalue problem

$$\sum_N A_{MN} a_N^{(i)} = \lambda_i a_M^{(i)} \tag{S91}$$

in terms of the symmetrized matrix $\hat{\tilde{A}}$ becomes

$$\sum_N \tilde{A}_{MN}\sqrt{\frac{P_M^{(\mathrm{eq})}}{P_N^{(\mathrm{eq})}}}\, a_N^{(i)} = \lambda_i a_M^{(i)}\,, \tag{S92}$$

which may be rewritten as

$$\sum_N \tilde{A}_{MN}\tilde{a}_N^{(i)} = \lambda_i \tilde{a}_M^{(i)}\,, \tag{S93}$$

with

$$\tilde{a}_K^{(i)} = \frac{a_K^{(i)}}{\sqrt{P_K^{(\mathrm{eq})}}}. \tag{S94}$$

Hence, both kinetic matrices have the same eigenvalues λ_i and the eigenvectors are related by eqn (S94). Since $\hat{\tilde{A}}$ is symmetric, its eigenvectors $\tilde{\mathbf{a}}^{(i)}$ are orthogonal, that is,

$$\sum_M \tilde{a}_M^{(i)}\tilde{a}_M^{(j)} = \delta_{i,j}. \tag{S95}$$

From eqn (33) we obtain an equation for the initial condition $P_M(0)$,

$$P_M(0) = \sum_i d_i a_M^{(i)}. \tag{S96}$$

Multiplication by $a_M^{(j)}/P_M^{(\mathrm{eq})}$ and summation over M gives

$$\sum_M \frac{P_M(0)}{P_M^{(\mathrm{eq})}} a_M^{(j)} = \sum_i d_i \sum_M \frac{a_M^{(i)}}{a_M^{(j)}} P_M^{(\mathrm{eq})} = \sum_i d_i \sum_M \tilde{a}_M^{(i)}\tilde{a}_M^{(j)} = d_j \tag{S97}$$

where in the last step we have used the orthogonality of $\tilde{\mathbf{a}}^{(i)}$ (eqn (S95)).

S9 Analysis of Contributions to TRCD and TA

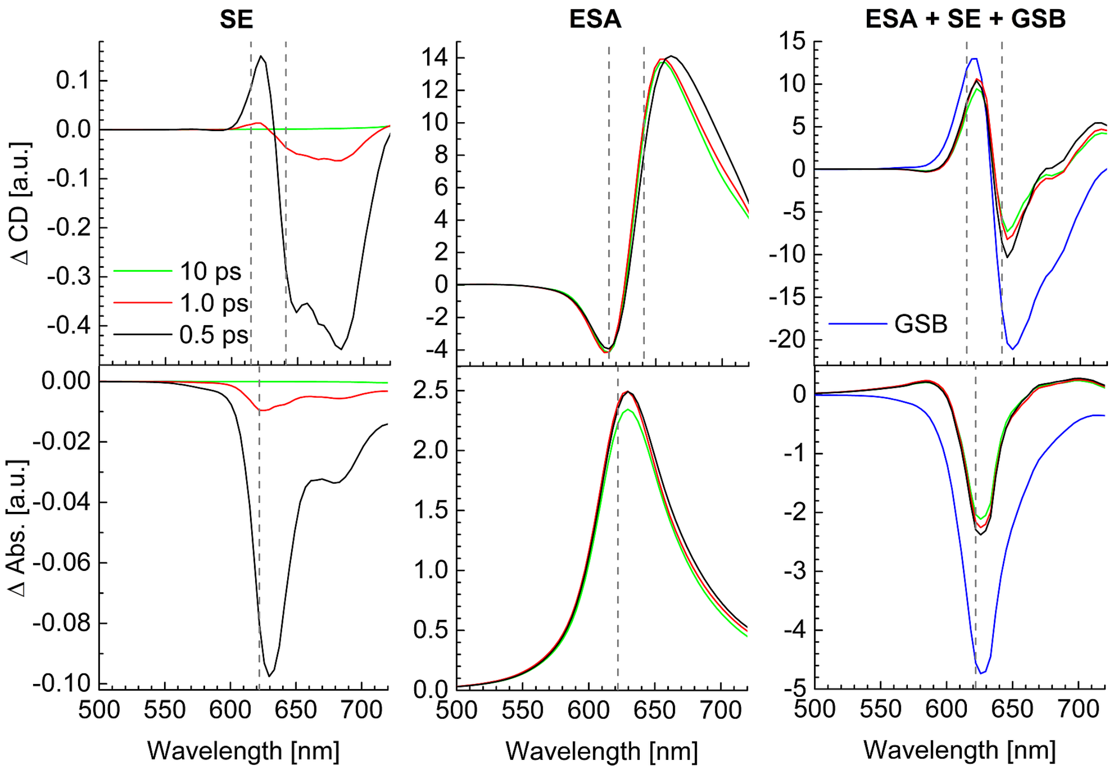

Figure S9: Analysis of the calculated TRCD (upper panels) and TA (lower panels) spectra for delay times 500 fs (black lines), 1 ps (red lines) and 10 ps (green lines), as indicated in the figure legend. The left column contains the SE contributions, the middle column the ESA contributions and the right column the GSB contribution (blue lines) and the sum of ESA, SE and GSB. Note that the GSB is practically identical for the first three delay times, investigated here, since the decay of excited states back to the ground state is much slower (140 ps). Dashed lines serve as a guide for the eye and indicate the positions of the main negative and positive peaks in the TRCD spectrum and of the main negative peak in the TA spectrum. Same dataset than in Fig. 10 but for a smaller wavelength region.

Figure S9 shows the contributions of GSB, ESA and SE to the TRCD and TA data within the wavelength region that is covered by our TRCD setup. More details are described in the main text in Section 4.3.

S10 Analysis of Exciton State Lifetimes of Random Coil and Mixed Helix/Zigzag Random Coil Configurations

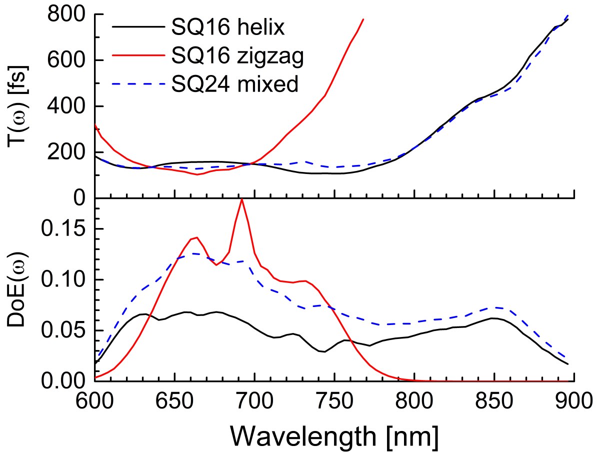

Figure S10: Comparison between the average frequency-resolved exciton state lifetime $T(\omega)$ (upper panel) and the density of exciton states DOE(ω) obtained for the squeezed helix in Fig. 9 of the main text with the corresponding quantities obtained for a zigzag random coil configuration of 16 squaraine chromophores (red lines) and a mixed configuration of 24 squaraine chromophores, with a squeezed helix configuration for the first 19 and a random coil configuration for the remaining 5 chromophores (dashed blue lines).

Figure S10 shows the frequency-resolved exciton state lifetimes for random coil as well as mixed helix and zigzag random coil configurations. More details are described in the main text in Section 4.3.

S11 Two Different Time Constants for the Non-Radiative Transition to the Electronic Ground State

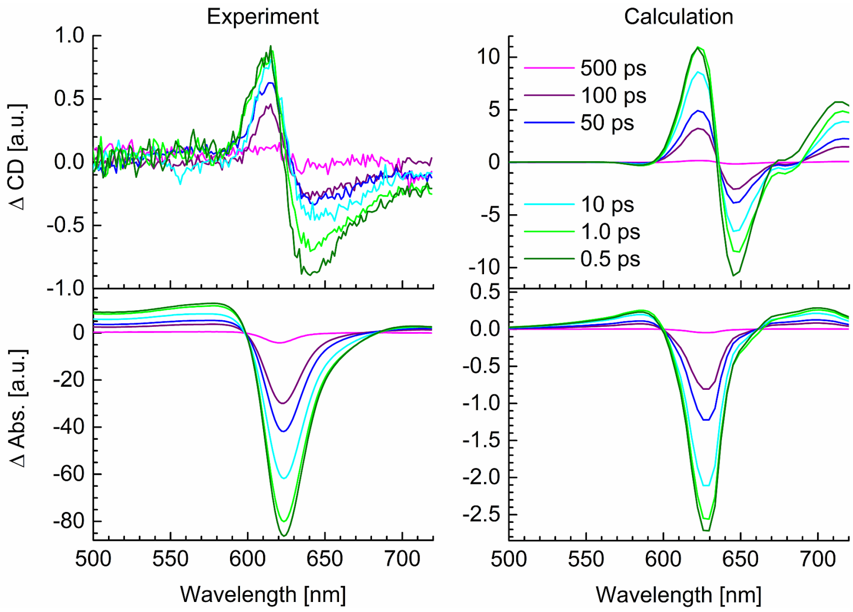

Figure S11: Same as in Fig. 11 but assuming that half of the helices in the sample are in a conformation that gives rise to a 140 ps time constant for the decay to the electronic ground state, as assumed before, and in the second half a conformation is realized with a 19.8 ps decay constant.

Figure S11 compares experimental TA and TRCD data with calculation data based on the assumption of two time constants: 140 ps and 19.8 ps.

S12 Comparison of TRCD and TA Spectra of Helix and Zigzag Random Coil Configurations

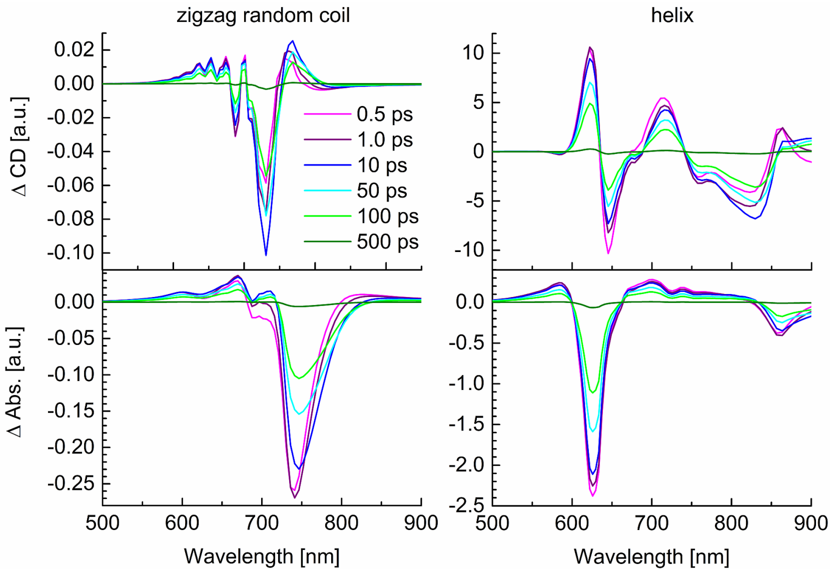

Figure S12: Comparison between TRCD (upper row) and TA (lower row) spectra, calculated for the zigzag random coil (left column) and the squeezed helix (right column), using the parameters in Table 2. We assumed an excitation by a 30 fs pump pulse centered at 633 nm. The delay times between pump and probe pulse are given in the figure legend. Note the different scaling of the y-axes.

Figure S12 shows the minor contribution of the zigzag random coil configuration and the major contribution of the helix configuration to the TRCD and TA data as explained in the main text in Section 4.3.

S13 Calculated Time-Resolved Data with Parameters of the Original Helix Model

Figure S13 includes calculated TA and TRCD data based on the original helix model. As shown in the main text in Fig. 8, the original helix model does not represent the linear absorption and CD spectra. Following this trend, the time-resolved data are not comparable with the experimental data in Fig. 5 and 10 in the main text. Whereas in the experiment the high-energy positive band in TRCD and the corresponding negative peak in TA occur around 625 nm, in the calculations in Fig. S13 they are shifted by 25 nm to longer wavelengths. In principle, this shift could be compensated in the calculations by a 25 nm blue shift of the site energy of the chromophores. Besides the problem that such a shift would be difficult to justify, it would bring the main negative band in the calculated TRCD spectrum from 725 nm to 700 nm, that is well into our experimental detection window. However, no negative peak at 700 nm is observed in the experimental TRCD spectrum, and thus the "original helix model" can be rejected as unsuitable.

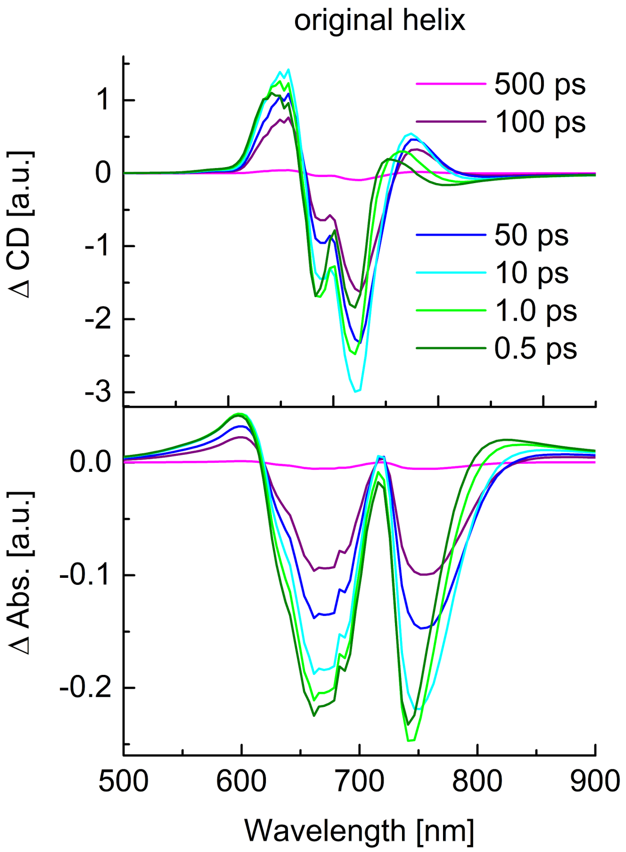

Figure S13: Calculated TRCD (top) and TA (bottom) spectra based on the parameters of the original helix model.

S14 Implicit Treatment of Charge Transfer States in Exciton Hamiltonian

In the following, we extend the exciton Hamiltonian in eqn (1) of the main text by including charge transfer (CT) states and motivate our implicit treatment of these states by a red-shift of the site energy of the chromophores and a rescaling of their excitonic couplings.

The one-exciton-CT Hamiltonian is introduced as

$$H^{(1)}_{\rm exc-CT} \;=\; H^{(1)}_{\rm exc,0} + \sum_k E^{\rm CT}_k |k^{\rm CT}\rangle\langle k^{\rm CT}| + \hat{V}^{\rm CT}, \tag{S98}$$

where $H^{(1)}_{\rm exc,0}$ is the one-exciton Hamiltonian of localized excited states $|m\rangle$

$$H^{(1)}_{\rm exc,0} = \sum_m E^{(0)}_m |m\rangle\langle m| + \sum_{m,n} V^{(0)}_{mn} |m\rangle\langle n|\,, \tag{S99}$$

and $\hat{V}^{\rm CT}$ contains the couplings between local excited and CT-states

$$\hat{V}^{\rm CT} = \sum_{k,m} (V^{\rm CT}_{km}\,|k^{\rm CT}\rangle\langle m| + {\rm h.c.})\,. \tag{S100}$$

In comparison to the one-exciton Hamiltonian $H^{(1)}_{\rm exc}$ in the main text (eqn (2)), we added a (super)script (0) to the energies and couplings to indicate that these energies do not yet include the influence of CT-states. $E^{\rm CT}_k$ is the energy of CT state $|k^{\rm CT}\rangle$, $V^{\rm CT}_{km}$ is the coupling between the latter and local excited state $|m\rangle$ and h.c. denotes the hermitian conjugate. In the following, the energies of the CT states $E^{\rm CT}_k$ are assumed to be large as compared to the energies $E^{(0)}_m$ of local excited states $|m\rangle$. In particular, it holds that $E^{\rm CT}_k - E_m >> V^{\rm CT}_{km}$. Hence, we can treat the coupling to CT states in perturbation theory and obtain the energies of local excited states as

$$E_m = E^{(0)}_m - \sum_k \frac{|V^{\rm CT}_{km}|^2}{E^{\rm CT}_k - E^{(0)}_m}\,. \tag{S101}$$

We see that the coupling to CT states lowers the site energies of the chromophores, if the CT state energies are larger than the energies of local excited states, as assumed here. This assumption is consistent with the red-shifted site energies in the squeezed helix, inferred from the fit of linear optical spectra (Fig. 8, Table 2).

Next, we consider the short-range contribution to the excitonic couplings V_{mn} between chromophores. For this purpose, we consider a dimer with equal local transition energies $E^{(0)}_m = E^{(0)}_n = E_0$. Without coupling to CT-states the eigenenergies of this dimer are

$$E^{(0)}_1 = E_0 + V^{(0)}_{mn}, \tag{S102}$$

and

$$E^{(0)}_2 = E_0 - V^{(0)}_{mn}, \tag{S103}$$

with the corresponding eigenstates

$$|\Psi_1\rangle = \frac{1}{\sqrt{2}}\,(|m\rangle + |n\rangle)\,, \tag{S104}$$

and

$$|\Psi_2\rangle = \frac{1}{\sqrt{2}}\,(|m\rangle - |n\rangle)\;. \tag{S105}$$

Taking into account the coupling to CT states in perturbation theory yields the following dimer energies

$$\begin{aligned} E_1 &= E_1^{(0)} - \sum_k \frac{|\langle \Psi_1 | V^{\mathrm{CT}} | k \rangle|^2}{E_k^{\mathrm{CT}} - E_1^{(0)}} \\ &= E_0 + V_{mn}^{(0)} - \frac{1}{2} \sum_k \frac{V_{mk}^{\mathrm{CT}} V_{km}^{\mathrm{CT}} + V_{nk}^{\mathrm{CT}} V_{kn}^{\mathrm{CT}} + V_{mk}^{\mathrm{CT}} V_{kn}^{\mathrm{CT}} + V_{nk}^{\mathrm{CT}} V_{km}^{\mathrm{CT}}}{E_k^{\mathrm{CT}} - E_0 - V_{mn}^{(0)}}, \end{aligned} \tag{S106}$$

and

$$\begin{aligned} E_2 &= E_2^{(0)} - \sum_k \frac{|\langle \Psi_2 | V^{\mathrm{CT}} | k \rangle|^2}{E_k^{\mathrm{CT}} - E_2^{(0)}} \\ &= E_0 - V_{mn}^{(0)} - \frac{1}{2} \sum_k \frac{V_{mk}^{\mathrm{CT}} V_{km}^{\mathrm{CT}} + V_{nk}^{\mathrm{CT}} V_{kn}^{\mathrm{CT}} - V_{mk}^{\mathrm{CT}} V_{kn}^{\mathrm{CT}} - V_{nk}^{\mathrm{CT}} V_{km}^{\mathrm{CT}}}{E_k^{\mathrm{CT}} - E_0 + V_{mn}^{(0)}}. \end{aligned} \tag{S107}$$

Taking into account that the coupling matrix elements are real, that is, $V_{mk}^{\mathrm{CT}} = V_{km}^{\mathrm{CT}}$ and that $E_k^{\mathrm{CT}} - E_0 >> V_{mn}^{(0)}$ we obtain

$$E_1 \approx E_0 + V_{mn}^{(0)} - \sum_k \frac{\left(V_{mk}^{\mathrm{CT}}\right)^2 \left(V_{kn}^{\mathrm{CT}}\right)^2 + V_{mk}^{\mathrm{CT}} V_{kn}^{\mathrm{CT}}}{E_k^{\mathrm{CT}} - E_0}, \tag{S108}$$

and

$$E_2 \approx E_0 - V_{mn}^{(0)} - \sum_k \frac{\left(V_{mk}^{\mathrm{CT}}\right)^2 \left(V_{kn}^{\mathrm{CT}}\right)^2 - V_{mk}^{\mathrm{CT}} V_{kn}^{\mathrm{CT}}}{E_k^{\mathrm{CT}} - E_0}. \tag{S109}$$

The effective excitonic coupling V_{mn} in the dimer is obtained as half the splitting between eigenstate energies giving

$$V_{mn} = \frac{E_1 - E_2}{2} = V_{mn}^{(0)} - \sum_k \frac{V_{mk}^{\mathrm{CT}} V_{kn}^{\mathrm{CT}}}{E_k^{\mathrm{CT}} - E_0}. \tag{S110}$$

The influence of CT states on the excitonic couplings is more difficult to estimate as that on the site energies in eqn (S101). As eqn (S110) shows, the second term containing the contribution of CT states can have the same as well as a different sign than the first term containing the coupling between local excited states $V_{mn}^{(0)}$. So, in principle, the second term could lead to a sign switch of the overall excitonic coupling. In this case, an H-aggregate could exhibit optical properties like a J-aggregate and vice versa.[7] In the present application to the squaraine helix, we find clear H-type aggregate behavior, since the low-energy transition around 850 nm has very little oscillator strength in the linear absorption spectrum (Fig. 8, bottom panels). Therefore, we can safely assume that the CT state couplings have the same sign as the excitonic couplings between localized excited states $V_{mn}^{(0)}$. For simplicity, we assume that the enhancement of the excitonic coupling by the CT states simply scales with $V_{mn}^{(0)}$, that is,

$$V_{mn} \approx f_{\mathrm{exc}} V_{mn}^{(0)}, \tag{S111}$$

where an enhancement factor $f_{\mathrm{exc}} = 2.4$ is inferred from a fit of the linear optical spectra (Fig. 8, Table 2).

We note the transition dipole moments of the chromophores $\vec{\mu}_m$ are not influenced by the CT-states since the latter are optically dark, that is $\vec{\mu}_{k^{\mathrm{CT}}} = \langle k^{\mathrm{CT}} | \hat{\mu} | 0 \rangle = 0$. Hence, the CD spectrum stays conservative in the low-energy region, despite the coupling of local excited states to high-energy CT-states.

The two-exciton Hamiltonian in eqn (6) is expressed in terms of site energies E_m and excitonic couplings V_{mn} that are interpreted in the same way, as above.

S15 Atomic Coordinates and Atomic Partial Charges

Table S2 includes the numerical values of atomic partial charges and coordinates of the squaraine monomer with a left-handed screw sense as described in the main text in Section 4.1. The atomic coordinates of the original helix, the squeezed helix, the average zig zag random coil structure and a mixed helix-random coil structure can be found in the accompanying files SQ16_original_helix_lh.pdb, SQ16_squeezed_helix.pdb, SQ16_zz_random_coil.pdb, and SQ24_mixed_sh_zz_rc.pdb, respectively.

Table S2: Atomic coordinates (in units of Å) and corresponding atomic partial charges (in units of the elementary charge e) of the geometry-optimized squaraine monomer. The CAM/B3LYP XC-functional and a 6-31G** basis set were used in the quantum chemical calculations using (time-dependent) density functional theory.

I	x	y	z	$q_I(0,1)$	$q_I(0,0)$	$q_I(1,1)$
1 C	-0.101961	0.202420	-0.387749	0.034605	0.094264	0.094693
2 C	-0.043240	1.587777	-0.280367	0.012907	0.005860	0.006077
3 C	1.178510	2.255022	-0.212469	0.034012	0.246703	0.243292
4 C	2.329749	1.484351	-0.256274	-0.053048	-0.527968	-0.531183
5 C	2.288291	0.098441	-0.362738	-0.053928	-0.933694	-0.914141
6 C	1.071321	-0.551763	-0.429040	0.027127	0.134386	0.129083
7 N	3.675658	1.891706	-0.207191	0.065902	0.432829	0.436280
8 C	4.523726	0.831510	-0.275400	0.017411	-0.666548	-0.807969
9 C	3.699079	-0.450652	-0.385440	0.070870	1.869384	1.854907
10 C	3.973468	-1.184186	-1.711225	0.012864	-0.241323	-0.244043
11 C	5.890091	1.047979	-0.239534	0.001577	0.264953	0.371423
12 C	6.983855	0.182570	-0.292866	0.069395	-1.188375	-1.443335
13 C	8.389763	0.514932	-0.251512	-0.009037	1.438060	1.445842
14 C	8.761950	-0.878237	-0.347539	-0.061690	-1.175189	-1.428070
15 C	7.334375	-1.246420	-0.395463	-0.002341	1.444355	1.598475
16 C	10.043923	-1.430066	-0.369693	0.003081	0.268227	0.373821
17 O	6.705225	-2.296184	-0.482713	0.000717	-0.855341	-0.784936
18 C	10.498575	-2.734768	-0.449286	-0.026208	-0.716523	-0.858991
19 N	11.835868	-2.979654	-0.447942	-0.060383	0.452677	0.457569
20 C	12.111871	-4.356682	-0.533904	0.051609	-0.552770	-0.557041
21 C	10.907183	-5.048919	-0.592829	0.056618	-0.929602	-0.908790
22 C	9.755644	-4.066952	-0.545850	-0.072935	1.964269	1.949426
23 C	13.336700	-5.005047	-0.561006	-0.034223	0.255203	0.251920
24 C	13.324382	-6.395764	-0.650609	-0.012818	-0.000364	-0.000195
25 C	12.128516	-7.103029	-0.709975	-0.034727	0.105587	0.106145
26 C	10.907879	-6.427483	-0.681387	-0.027722	0.115102	0.109415
27 C	12.852121	-1.946104	-0.371209	-0.033039	0.197886	0.192713
28 C	4.107901	3.273286	-0.100365	0.031821	0.199508	0.194531
29 C	9.100814	1.701964	-0.156224	0.005829	-1.216826	-0.899436
30 C	8.880515	-4.313552	0.696584	-0.012322	-0.263387	-0.266024
31 C	10.522009	1.705755	-0.134907	0.012530	0.710765	0.669634
32 N	11.682715	1.668323	-0.119881	0.006341	-0.570150	-0.501369
33 C	8.431438	2.953292	-0.075727	-0.016062	0.715243	0.674074
34 N	7.849301	3.956217	-0.011435	-0.005616	-0.572532	-0.503887
35 C	3.940487	-1.377941	0.819659	0.013095	-0.240992	-0.243615
36 C	8.920216	-4.150499	-1.836432	-0.012212	-0.263679	-0.266295

References

[1] C. Oh and M. J. Escuti, *Opt. Lett.*, 2008, **33**, 2287–2289.

[2] N. M. Kearns, R. D. Mehlenbacher, A. C. Jones and M. T. Zanni, *Opt. Express*, 2017, **25**, 7869–7883.

[3] C. Lambert, F. Koch, S. F. Völker, A. Schmiedel, M. Holzapfel, A. Humeniuk, M. I. S. Röhr, R. Mitric and T. Brixner, *J. Am. Chem. Soc.*, 2015, **137**, 7851–7861.

[4] S. S. Andrews, *J. Chem. Educ.*, 2004, **81**, 877–885.

[5] D. Lindorfer and T. Renger, *J. Phys. Chem. B*, 2018, **122**, 2747–2756.

[6] D. L. Andrews and T. Thirunamachandran, *J. Chem. Phys.*, 1977, **67**, 5026–5033.

[7] N. J. Hestand and F. C. Spano, *Chem. Rev.*, 2018, **118**, 7069–7163.
